# Supplementary material for: Reaction blueprints and logical control flow for parallelized chiral synthesis in the Chemputer
Source: Nat Commun. 2024 Nov 26;15:10261. doi: 10.1038/s41467-024-54238-6 (PMC11599859; doi:10.1038/s41467-024-54238-6)
Supplement: Supplementary file 1 — Supplementary Information [file 41467_2024_54238_MOESM1_ESM.pdf]

Supplementary Information for

Reaction Blueprints and Logical Control Flow for Parallelized Chiral Synthesis in the Chemputer

Mindaugas Šiaučiulis, Christian Knittl-Frank, S. Hessam M. Mehr, Emma Clarke, Leroy Cronin*

Corresponding author: lee.cronin@glasgow.ac.uk

The PDF file includes:

Supplementary Methods

Supplementary References

Contents

Supplementary Methods 3
Manual experimental procedures 6
SI-1 1-(4-iodophenyl)-N,N-dimethylmethanamine 6
SI-2 (E)-2-(2-nitrovinyl)furan 6
Automated experimental procedures 8
(S)-Cat-1 (S)-2-(bis(3,5-bis(trifluoromethyl)phenyl)((trimethylsilyl)oxy)methyl)pyrrolidine 8
(S)-Cat-2 (S)-2-(diphenyl((trimethylsilyl)oxy)methyl)pyrrolidine 13
(S)-Cat-3 (S)-1,1'-((pyrrolidin-2-yl((trimethylsilyl)oxy)methylene)bis(4,1-phenylene))bis(N,N-dimethylmethanamine) 18
1a (S)-2-isopropyl-5-oxohexanal 22
1b (R)-5-oxo-2-propylhexanal 25
2 (S)-2-chloro-3-phenylpropan-1-ol 28
4 ((2R,3R)-3-phenyloxiran-2-yl)methanol 32
5 Methyl (S)-3-phenyl-4-(pyridin-2-yl)butanoate 35
6 (S)-4-nitro-3-phenylbutanal 39
7a (1'S,2'R,3'R)-2'-nitro-1',2',3',6'-tetrahydro-[1,1':3',1''-terphenyl]-4'-carbaldehyde 42
7b (1'S,2'S,3'R)-2'-nitro-1',2',3',6'-tetrahydro-[1,1':3',1''-terphenyl]-4'-carbaldehyde 42
8 (R)-1-((1S,2S)-1-(benzylamino)-1,2-dihydronaphtho[2,1-b]furan-2-yl)pentan-1-ol 48
9a (R)-2-((S)-2-nitro-1-phenylethyl)pentanal 51
9a (R)-2-((S)-2-nitro-1-phenylethyl)pentanal 58
9b (2R,3S)-2-ethyl-4-nitro-3-phenylbutanal 58
9c (2R,3R)-2-ethyl-3-(furan-2-yl)-4-nitrobutanal 58
9d (R)-2-((R)-1-(furan-2-yl)-2-nitroethyl)pentanal 58
NMR spectra 70
SI-1 70
SI-2 71
(S)-Cat-1 72
(S)-Cat-2 73
(S)-Cat-3 74
1a 75
1b 76
2 77
4 78
5 79
6 80
7a 81
7b 82
8 83
9a 84
9b 85
9c 86
9d 87
Supplementary References 88

Supplementary Methods

Solvents and reagents were used as received from commercial suppliers unless otherwise stated. Dry tetrahydrofuran was purchased from commercial suppliers and used as received.

NMR measurements were performed with Bruker Avance III HD 600 spectrometer operating at 600.1 and 150.9 MHz for ^{1}H and ^{13}C, respectively. Spectra were collected at 298 K, chemical shifts are reported in ppm and were calibrated for the (residual) NMR solvent signal (multiplicities are given as s: singlet, d: doublet, t: triplet, q: quartet, m: multiplet, with coupling constants reported in Hz). The spectra were processed with MestreNova 14.0.0.

Chiral HPLC analysis was performed on a Thermo Scientific Dionex Ultimate 3000 equipped with an Ultimate 3000 pump, an Ultimate 3000 autosampler, an Ultimate 3000 column compartment and an Ultimate 3000 variable wavelength detector. 10 µL of each sample was injected onto a Daicel CHIRALPAK IB N-5 (5 µm, 4.6 mm × 150 mm), Daicel CHIRALPAK IC (5 µm, 4.6 mm × 250 mm), or Chiral-Art Cellulose-C (5 µm, 4.6 mm × 250 mm) column. Details of chromatographic conditions are indicated under each compound. Data was analysed using the Chromeloen v6.80 SR12.

Hardware build instructions for Chemputer platform have been extensively described before(*1*) and no modifications were required for this study.

Software stack needed to control Chemputer devices, compile and run XDL files has been described before.(*1*)

All XDL (.xdl), graph (.json), and the resulting compiled XDL (.xdlexe) files for all automated syntheses are provided as supplementary material “Data S1”. **Note:** compiled XDL files (containing full sequence of unit operations in chronological order) for protocols involving queues are not generated as scheduling is resolved during run-time and is not known *a priori*.

Execution of automated synthesis files

A full synthesis definition for the Chemputer system consists of a XDL procedure, a graph file, and a short Python execution script.

The XDL procedure was written using an editor of choice (e.g. Visual Studio Code, Atom) and saved in XML format as `.xdl` files.

The graphs for all syntheses were constructed through the ChemIDE web application GUI(*2*) and saved in JSON format as `.json` files.

A general Python script was used to execute all of the procedures outlined in Listing S1.

Listing S1: General Python script used to execute procedures.

```
from os import path

import ChemputerterAPI
from chempiler import Chempiler
from Chemputerterxdl import ChemputerterPlatform
from xdl import XDL

# Optionally, the procedure can be simulated without connection to the
physical devices
simulation = False
root = path.abspath(path.curdir)
output_dir = path.join(root, 'simulation_logs') if simulation else
path.join(root, 'logs')

# Definition of reaction XDL synthesis script and graph input files
experiment_name = 'experiment_name'
xdl_file = experiment_name+'.xdl'
graph_file = experiment_name+'.json'

# Loading of XDL synthesis script
x = XDL(xdl_file, platform=ChemputerterPlatform)
# Compilation of XDL synthesis script for a particular hardware configuration
x.prepare_for_execution(graph_file)

# Initialisation of the platform controller - connect to and reset the devices
c = Chempiler(experiment_name, graph_file, output_dir, simulation,
[ChemputerterAPI])

# Execution of the compiled XDL object using the platform controller
x.execute(c)
```

The above script can be saved as `.py` file and run as a stand-alone python script. Alternatively, the code can be run within an interactive python notebook (Jupyter). Running the code within a notebook allows for a convenient section-wise execution, e.g. for connecting to and initialising hardware devices without commencing the

chemical synthesis and/or controlling the devices through low-level commands for troubleshooting, cleaning, or other purposes. Running syntheses from an interactive notebook is highly recommended in most cases and was done for all automated syntheses described below.

Manual experimental procedures

SI-1 1-(4-iodophenyl)-N,N-dimethylmethanamine

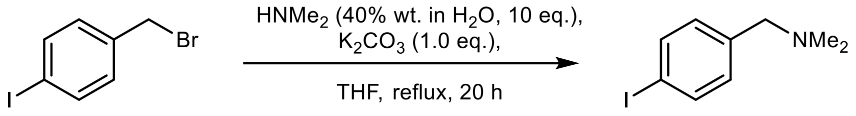

Procedure adapted from literature.(*3*)

To a flask containing 4-iodobenzyl bromide (50.0 g, 168.4 mmol) and potassium carbonate (23.2 g, 168.4 mmol) was added THF (300 mL) and the mixture was vigorously stirred. To the suspension was added dimethyl amine (40% wt. in H_2O, 213 mL, 1.68 mol) and the biphasic mixture was refluxed for 20 hours with vigorous stirring. The reaction was cooled to room temperature, and the mixture was extracted with chloroform (3 x 250 mL). The combined organic extracts were dried over $MgSO_4$, and the solvent was removed *in vacuo* to give a pale-yellow oil with white solid (overalkylation product – ammonium salt) suspended in the oil. To the mixture was added diethyl ether (50 mL), and the solids were removed by filtration. The filtrate was concentrated *in vacuo* to afford the title product as a pale-yellow oil, which may solidify on standing (42.9 g, 164 mmol, 98%).

^{1}H NMR (600 MHz, $CDCl_3$) δ 7.68 – 7.61 (m, 2H), 7.09 – 7.01 (m, 2H), 3.35 (s, 2H), 2.22 (s, 6H).

^{13}C NMR (151 MHz, $CDCl_3$) δ 138.9, 137.5, 131.2, 92.5, 64.0, 45.5.

The analytical data is in accordance with that previously reported in the literature.(*4*)

SI-2 (E)-2-(2-nitrovinyl)furan

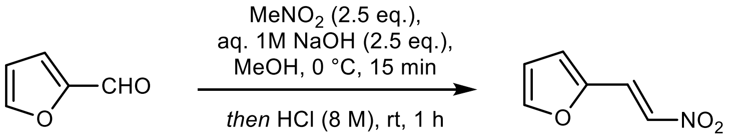

Procedure adapted from literature.(*5*)

A solution of furfural (16.6 mL, 200 mmol), nitromethane (26.8 mL, 500 mmol, 2.5 eq.) in methanol (40 mL) was cooled to 0 °C. Aqueous 1 M sodium hydroxide solution (20.8 g in 520 mL H_2O, 2.5 eq.) was added slowly over 10 minutes, then ice/water (*ca.* 100

mL) was added to the reaction mixture and the stirring was continued for 15 minutes. The ice-cold mixture was then poured slowly into vigorously stirred 8 M HCl (200 mL), resulting in an immediate heavy precipitation. The mixture was stirred at room temperature for 1 hour, after which time the solid was filtered, redissolved in CH_2Cl_2 (250 mL) and the organic solution was washed with brine (2 x 150 mL). The organic extract was dried over $MgSO_4$ and the solvent was removed *in vacuo* to afford the title product as an orange solid (19.4 g, 139 mmol, 70%).

^{1}H NMR (600 MHz, $CDCl_3$) δ 7.77 (d, *J* = 13.2 Hz, 1H), 7.59 (d, *J* = 1.7 Hz, 1H), 7.52 (d, *J* = 13.2 Hz, 1H), 6.89 (d, *J* = 3.5 Hz, 1H), 6.58 (dd, *J* = 3.6, 1.8 Hz, 1H).

^{13}C NMR (151 MHz, $CDCl_3$) δ 147.0, 146.9, 135.2, 125.6, 120.1, 113.5.

The analytical data is in accordance with that previously reported in the literature.(*5*)

Automated experimental procedures

(S)-Cat-1
(S)-2-(bis(3,5-bis(trifluoromethyl)phenyl)((trimethylsilyl)oxy)methyl)pyrrolidine

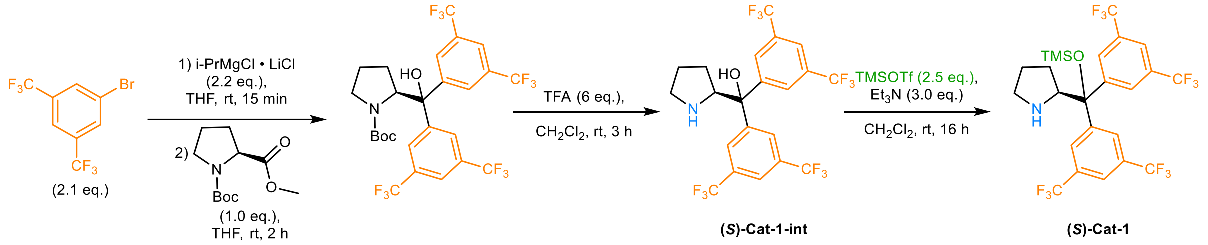

Manual preparations

The system was configured as specified in the graph file `(S)-Cat-1.json` for the reaction.

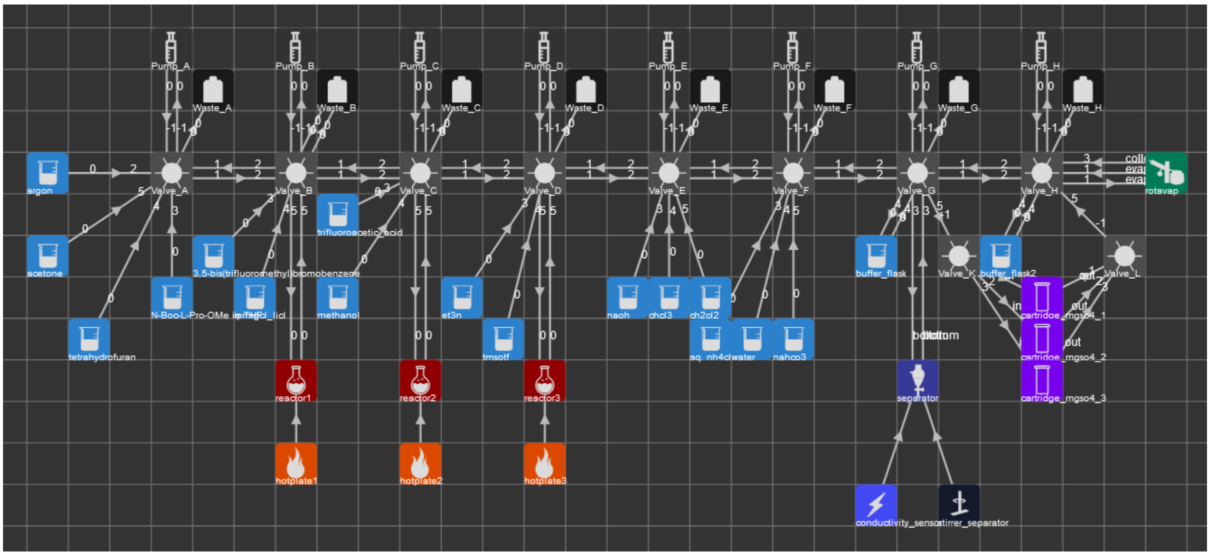

Figure S1: Graphical representation of `(S)-Cat-1.json`

All reagent flasks were connected to a passive (non-controllable) argon supply.

Automated synthesis

The synthesis was encoded into three independent Blueprints – one Blueprint per conventional synthetic step. The following is an automated human-readable print out of the encoded steps for each of the blueprints.

Note: The step list below includes the parameters and properties passed into the blueprints for this synthesis and is not representative of a general Blueprint.

Note: Execution of some steps is parallelized. Steps which occur in parallel to previous steps without waiting for them to finish are indicated with an asterisk (*) after the step index.

For general blueprints and full synthesis script refer to synthesis file **`(S)-Cat-1.xdl`**.

Blueprint *GrignardAddition:*

```
Step 1: Reset liquid handling apparatus with tetrahydrofuran (3 x 3 mL).
Step 2: Add 3,5-bis(trifluoromethyl)bromobenzene (2.1 eq.) directly to reactor1 at default speed without stirring. Flush liquid handling apparatus with tetrahydrofuran (5 mL) after addition.
Step 3: Add iprmgcl_licl (2.2 eq.) directly to reactor1 at default speed with stirring at 250 RPM. Flush liquid handling apparatus with tetrahydrofuran (3 mL) after addition.
Step 4: Stir reactor1 for 15 min at 250 RPM stopping stirring afterwards.
Step 5: Add N-Boc-L-Pro-OMe in THF (1.0 eq.) directly to reactor1 at default speed with stirring at 250 RPM. Flush liquid handling apparatus with tetrahydrofuran (5 mL) after addition.
Step 6: Stir reactor1 for 3 h at 250 RPM stopping stirring afterwards.
Step 7: Add aq. nh4cl (25 mL) directly to reactor1 at default speed with stirring at 400 RPM.
Step 8: Add water (15 mL) directly to reactor1 at default speed with stirring at 400 RPM.
Step 9: Transfer all from reactor1 directly to separator at default speed, flushing tubing after the transfer.
Step 10: Repeat 2 times:
    Add chcl3 (20 mL) directly to reactor1 at default speed with stirring at 350 RPM.
    Add water (10 mL) directly to reactor1 at default speed with stirring at 350 RPM.
    Transfer all from reactor1 directly to separator at default speed, flushing tubing after the transfer.
Step 11: Extract contents of separator with chcl3 (2 x 40 mL). Transfer waste phase (top) to waste, and product phase (bottom) directly to separator.
Step 12: Transfer all from separator directly to rotavap at default speed, rinsing separator with chcl3 (1 x 30 mL), without flushing tubing after the transfer.
Step 13: Reset liquid handling apparatus with chcl3 (3 x 3 mL).
Step 14: Evaporate contents of rotavap with default pressure control at temperature 40 °C for 60 min.
Step 15: Evaporate contents of rotavap with pressure 1 mbar at temperature 40 °C for 15 min.
```

Blueprint *BocDeprotection_tfa*:

```
Step 1: Repeat 2 times:
    Dissolve contents of rotavap in ch2cl2 (20 mL) over 5 min, stirring at 280 RPM.
    Transfer 30 mL from rotavap directly to reactor2 at default speed, flushing tubing after the transfer.
Step 2: Add trifluoroacetic_acid (6 eq.) directly to reactor2 at default speed with stirring at 250 RPM.
Step 3: Stir reactor2 for 3 h at 250 RPM stopping stirring afterwards.
Step 4*: Clean rotavap with methanol (1 × 60 mL) without temperature control, without drying, stirring for 60 s at 150 RPM.
Step 5*: Clean rotavap with ch2cl2 (1 × 30 mL) without temperature control, without drying, stirring for 60 s at 150 RPM.
Step 6: Add naoh (10 eq.) directly to reactor2 at default speed with stirring at 250 RPM.
```

Step 7: Add water (50 mL) directly to reactor2 at default speed with stirring at 250 RPM.
Step 8: Add ch2cl2 (30 mL) directly to reactor2 at default speed with stirring at 250 RPM.
Step 9: Stir reactor2 for 10 min at 600 RPM stopping stirring afterwards.
Step 10: Transfer all from reactor2 directly to separator at default speed, rinsing reactor2 with ch2cl2 (1 x 30 mL), without flushing tubing after the transfer.
Step 11: Extract contents of separator with ch2cl2 (2 x 40 mL). Transfer waste phase (top) to waste, and product phase (bottom) directly to separator.
Step 12: Transfer all from separator directly to rotavap at default speed, rinsing separator with ch2cl2 (1 x 30 mL), without flushing tubing after the transfer.
Step 13: Reset liquid handling apparatus with ch2cl2 (3 x 3 mL).
Step 14: Evaporate contents of rotavap with default pressure control at temperature 40 °C for 2 h.
Step 15: Evaporate contents of rotavap with pressure 1 mbar at temperature 45 °C for 30 min.

Blueprint *TmsProtection*:

Step 1: Repeat 2 times:
    Add ch2cl2 (20 mL) directly to rotavap at default speed with stirring at 250 RPM.
    Transfer all from rotavap directly to reactor3 at default speed, flushing tubing after the transfer.
Step 2: Add et3n (3.0 eq.) directly to reactor3 at default speed with stirring at 250 RPM. Flush liquid handling apparatus with ch2cl2 (3 mL) after addition.
Step 3: Add tmsotf (2.5 eq.) directly to reactor3 at default speed with stirring at 250 RPM. Flush liquid handling apparatus with ch2cl2 (3 mL) after addition.
Step 4: Stir reactor3 for 16 h at 250 RPM stopping stirring afterwards.
Step 5*: Clean rotavap with ch2cl2 (1 × 30 mL) without temperature control, without drying, stirring for 60 s at 150 RPM.
Step 6: Transfer all from reactor3 directly to separator at default speed, rinsing reactor3 with ch2cl2 (2 x 20 mL), without flushing tubing after the transfer.
Step 7: Wash contents of separator with nahco3 (2 x 80 mL). Transfer waste phase (top) to waste, and product phase (bottom) directly to separator.
Step 8: Transfer all from separator directly to rotavap at default speed, rinsing separator with ch2cl2 (1 x 30 mL), without flushing tubing after the transfer.
Step 9: Reset liquid handling apparatus with ch2cl2 (3 x 3 mL).
Step 10: Evaporate contents of rotavap with default pressure control at temperature 40 °C for 60 min.
Step 11: Evaporate contents of rotavap with pressure 1 mbar at temperature 45 °C for 30 min.

Purification

The crude product obtained from the automated synthesis was purified by column chromatography (25→50% Et_2O/PE) to afford the title compound as an off-white solid (3.49 g, 5.84 mmol, 58%).

Analytical data

^{1}H NMR (600 MHz, $CDCl_3$) δ 8.03 – 7.98 (m, 2H), 7.83 (d, *J* = 6.5 Hz, 2H), 7.77 (d, *J* = 1.6 Hz, 2H), 4.22 (t, *J* = 7.3 Hz, 1H), 2.93 (ddd, *J* = 10.2, 7.6, 6.4 Hz, 1H), 2.64 – 2.50 (m, 1H), 1.73 – 1.64 (m, 2H), 1.58 – 1.48 (m, 1H), 1.48 – 1.41 (m, 1H), 1.11 (ddq, *J* = 12.3, 8.3, 6.4 Hz, 1H), -0.08 (s, 9H).
^{13}C NMR (151 MHz, $CDCl_3$) δ 148.4, 146.6, 131.8 (q, *J* = 33.4 Hz), 131.0 (q, *J* = 33.3 Hz), 128.8 (d, *J* = 4.0 Hz), 128.3 (d, *J* = 3.9 Hz), 123.6 (q, *J* = 272.7 Hz), 123.4 (q, *J* = 272.9 Hz), 121.9 (hept, *J* = 3.8 Hz), 121.7 (hept, *J* = 3.8 Hz), 82.5, 64.5, 47.4, 27.7, 25.4, 2.1.

The analytical data is in accordance with that previously reported in the literature.(*6*)

Synthesis of racemate

The racemic catalyst was prepared by performing the Grignard addition/Deprotection/Silylation sequence manually using 1:1 mixture of N-Boc-D-Pro-OMe and N-Boc-L-Pro-OMe as the starting material. An alternative Boc deprotection procedure was used: the intermediate obtained after the Grignard addition step (ca. 15 mmol) was dissolved in ethanol (60 mL) and added to a flask containing NaOH (10 eq.). The mixture was refluxed for 3 hours, then cooled to room temperature, diluted with water (90 mL) and most of the ethanol was removed *in vacuo*. The remaining mixture was extracted with diethyl ether (3 x 90 mL), the combined extracts were dried over $MgSO_4$, and the solvent was removed *in vacuo* to afford the crude product. At this stage, the crude product was purified by redissolving the crude material in diethyl ether (30 mL) and adding 4M HCl in dioxane (4 mL, 16 mmol). The resulting precipitate was filtered and washed with hexane (2 x 30 mL) to afford ***rac*-Cat-1-int·HCl** as an off-white solid (5.21 g, 9.28 mmol, 62%). The free amine intermediate was obtained as follows: the salt was dissolved in a 1:1 mixture of CH_2Cl_2/sat. aq. $NaHCO_3$ (250 mL total). The layers were separated, and the aqueous layer was washed with CH_2Cl_2 (100 mL). The combined organics were dried over $MgSO_4$ and the solvent was removed *in vacuo* to afford ***rac*-Cat-1-int** as an orange oil (3.46 g, 6.59 mmol, 44% over two steps).* Silylation of ***rac*-Cat-1-int** afforded ***rac*-Cat-1** as an off-white solid (3.70 g, 6.20 mmol, 94%, 41% over 3 steps) and the racemic catalyst was used without further purification.

*The analogous automated two-step Grignard addition/Deprotection sequence (performed on 11 mmol scale) using the NaOH protocol without the intermediate purification afforded intermediate **(*S*)-Cat-1-int** (4.54 g, 8.65 mmol, 79% over two steps). The use of ethanol/NaOH deprotection protocol was observed to be somewhat inconsistent - the success of the automated conductivity-based aqueous workup was highly dependent on the amount of ethanol remaining in the reaction mixture, while evaporation of the reaction mixture often resulted in the formation of large quantities of solids in the rotavap which may lead to blockages. Additionally, the requirement to

load solid reagent (NaOH) prior to the reaction, prompted us to develop an improved process for automation, hence the use of acid-mediated deprotection in the final automated protocols. The racemate synthesis was not repeated using the final automation protocols.

tabl(S)-Cat-2
(S)-2-(diphenyl((trimethylsilyl)oxy)methyl)pyrrolidine

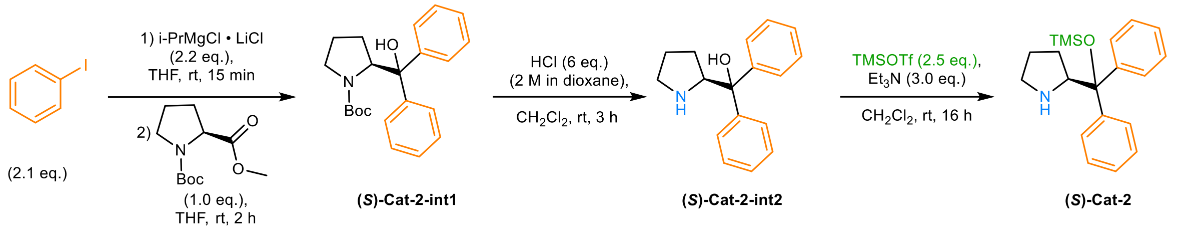

Optimisation

Initially, the blueprints used in the synthesis of **(*S*)-Cat-1** were used without any changes, with iodobenzene as the arylhalide. The process did not afford the desired product and instead oxazolidinone **SI-3** was isolated as the sole product in the reaction.

^{1}H NMR (600 MHz, $CDCl_3$) δ 7.52 (dd, *J* = 8.4, 1.3 Hz, 2H), 7.39 – 7.27 (m, 8H), 4.55 (dd, *J* = 10.4, 5.5 Hz, 1H), 3.73 (dt, *J* = 11.5, 8.1 Hz, 1H), 3.25 (ddd, *J* = 11.5, 9.6, 3.7 Hz, 1H), 2.04 – 1.95 (m, 1H), 1.87 (dddt, *J* = 13.1, 11.2, 9.7, 7.4 Hz, 1H), 1.72 (dddd, *J* = 12.7, 7.3, 5.5, 2.0 Hz, 1H), 1.13 (dtd, *J* = 12.8, 10.8, 8.7 Hz, 1H).

^{13}C NMR (151 MHz, $CDCl_3$) δ 160.6, 143.5, 140.5, 128.8, 128.5, 128.5, 127.9, 126.2, 125.7, 86.1, 69.5, 46.2, 29.2, 25.1.

The analytical data is in accordance with that previously reported in the literature.(*7*)

Although it has been reported that the reaction of *N*-Boc-Pro-OMe with excess Grignard reagents at elevated temperatures can lead to the formation of oxazolidinones,(*8*) it was considered unlikely given the room-temperature conditions and the well-behaved reactivity in the synthesis of **(*S*)-Cat-1**. Instead, it was reasoned that the oxazolidinone might arise from an undesired side-reaction during the acidic Boc-deprotection step. To verify this, the Grignard addition step was performed separately following the standard protocol and afforded the expected intermediate **(*S*)-Cat-int1** both manually and in an automated run. The intermediate was then subjected to the TFA-mediated deprotection conditions and was cleanly converted to oxazolidinone **SI-3**. Therefore, an alternative deprotection protocol was sought.

A short screen of acids for the deprotection step was performed:

Table S1: Screening of acids for the Boc deprotection

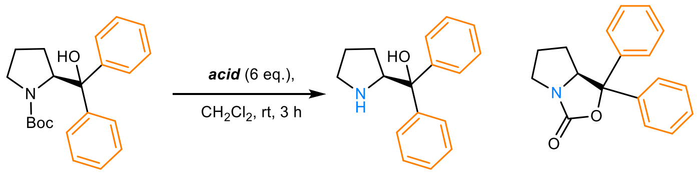

(*S*)-Cat-2-int1 (*S*)-Cat-2-int2 SI-3

Acid	Conversion*	(*S*)-Cat-2-int2 : SI-3
TFA	>99%	0 : 100
Formic acid	0%	N/A
MSA	>99%	41 : 59
p-TSA·H_2O	>99%	66 : 34
10-CSA	29%	48 : 52
4M HCl in dioxane	>99%	70 : 30
2M HCl in dioxane**	>99%	94 : 6

*Conversion measured as the ratio of **(*S*)-Cat-2-int1** converted to **(*S*)-Cat-2-int2** and **SI-3** by ^{1}H NMR of the crude product.
**2 M HCl in dioxane was obtained by diluting commercial 4 M HCl in dioxane with additional dioxane in a ratio of 1:1 v/v.
MSA = methanesulfonic acid, *p*-TSA = *para*-Toluenesulfonic acid, 10-CSA = 10-camphorsulfonic acid

As a result, 2M HCl solution in dioxane was used as the acid reagent for the deprotection step in the automated synthesis. Note that no changes to the blueprints are required between synthesis of **(*S*)-Cat-2** and **(*S*)-Cat-3** (which used 4M HCl solution) – the reagent properties were defined in the main *Reagent* section, and all quantities were calculated automatically based on the defined molarity.

Manual preparations

The system was configured as specified in the graph file `(S)-Cat-2.json` for the reaction.

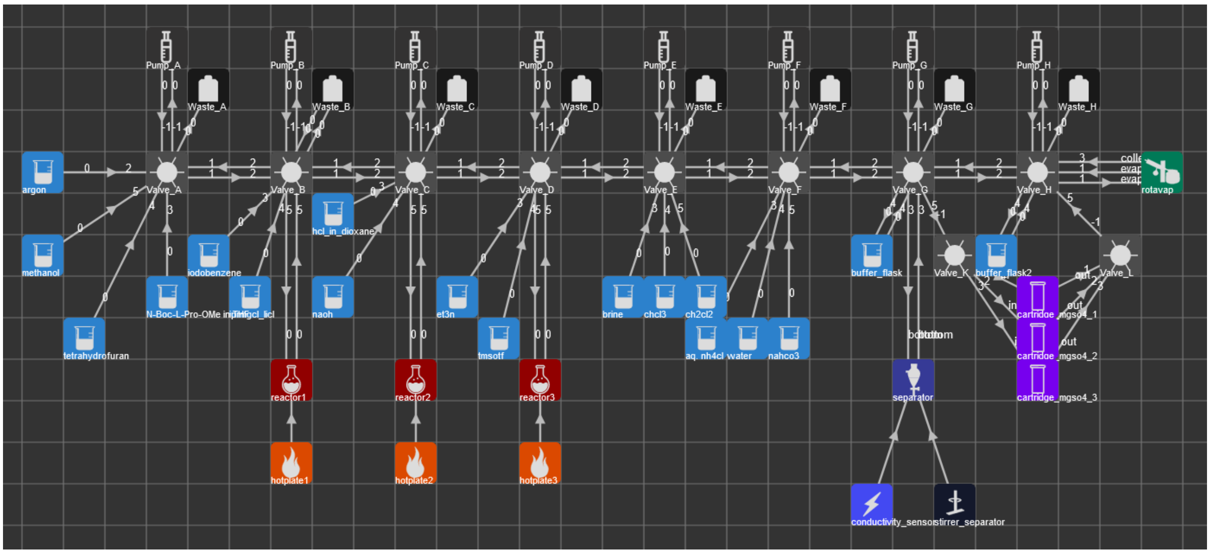

Figure S2: Graphical representation of `(S)-Cat-2.json`

All reagent flasks were connected to a passive (non-controllable) argon supply.

Automated synthesis

The synthesis was encoded into three independent Blueprints – one Blueprint per conventional synthetic step. The following is an automated human-readable print out of the encoded steps for each of the blueprints.

Note: The step list below includes the parameters and properties passed into the blueprints for this synthesis and is not representative of a general Blueprint.

Note: Execution of some steps is parallelized. Steps which occur in parallel to previous steps without waiting for them to finish are indicated with an asterisk (*) after the step index.

For general blueprints and full synthesis script refer to synthesis file `(S)-Cat-2.xdl`

Blueprint *GrignardAddition:*

```
Step 1: Reset liquid handling apparatus with tetrahydrofuran (3 x 3 mL).
Step 2: Add iodobenzene (2.1 eq.) directly to reactor1 at default speed without stirring. Flush liquid handling apparatus with tetrahydrofuran (5 mL) after addition.
Step 3: Add iprmgcl_licl (2.2 eq.) directly to reactor1 over 5 min with stirring at 250 RPM. Flush liquid handling apparatus with tetrahydrofuran (3 mL) after addition.
Step 4: Stir reactor1 for 15 min at 250 RPM stopping stirring afterwards.
Step 5: Add N-Boc-L-Pro-OMe in THF (1.0 eq.) directly to reactor1 at default speed with stirring at 250 RPM. Flush liquid handling apparatus with tetrahydrofuran (5 mL) after addition.
Step 6: Stir reactor1 for 3 h at 250 RPM stopping stirring afterwards.
```

Step 7: Add aq. nh4cl (25 mL) directly to reactor1 at default speed with stirring at 400 RPM.
Step 8: Add water (15 mL) directly to reactor1 at default speed with stirring at 400 RPM.
Step 9: Transfer all from reactor1 directly to separator at default speed, flushing tubing after the transfer.
Step 10: Repeat 2 times:
    Add chcl3 (20 mL) directly to reactor1 at default speed with stirring at 350 RPM.
    Add water (10 mL) directly to reactor1 at default speed with stirring at 350 RPM.
    Transfer all from reactor1 directly to separator at default speed, flushing tubing after the transfer.
Step 11: Extract contents of separator with chcl3 (2 x 40 mL). Transfer waste phase (top) to waste, and product phase (bottom) directly to separator.
Step 12: Transfer all from separator directly to rotavap at default speed, rinsing separator with chcl3 (1 x 30 mL), without flushing tubing after the transfer.
Step 13: Reset liquid handling apparatus with chcl3 (3 x 3 mL).
Step 14: Evaporate contents of rotavap with default pressure control at temperature 40 °C for 60 min.
Step 15: Evaporate contents of rotavap with pressure 1 mbar at temperature 40 °C for 15 min.

Blueprint *BocDeprotection:*

Step 1: Repeat 2 times:
    Dissolve contents of rotavap in ch2cl2 (15 mL) over 5 min, stirring at 280 RPM.
    Transfer 30 mL from rotavap directly to reactor2 at default speed, flushing tubing after the transfer.
Step 2: Add hcl_in_dioxane (6 eq.) directly to reactor2 at default speed with stirring at 250 RPM.
Step 3: Stir reactor2 for 3 h at 250 RPM stopping stirring afterwards.
Step 4*: Clean rotavap with ch2cl2 (1 × 30 mL) without temperature control, without drying, stirring for 60 s at 150 RPM.
Step 5: Add naoh (10 eq.) directly to reactor2 at default speed with stirring at 250 RPM.
Step 6: Add water (50 mL) directly to reactor2 at default speed with stirring at 250 RPM.
Step 7: Add ch2cl2 (30 mL) directly to reactor2 at default speed with stirring at 250 RPM.
Step 8: Stir reactor2 for 10 min at 600 RPM stopping stirring afterwards.
Step 9: Transfer all from reactor2 directly to separator at default speed, rinsing reactor2 with ch2cl2 (1 x 30 mL), without flushing tubing after the transfer.
Step 10: Extract contents of separator with ch2cl2 (2 x 40 mL). Transfer waste phase (top) to waste, and product phase (bottom) directly to separator.
Step 11: Wash contents of separator with brine (2 x 40 mL). Transfer waste phase (top) to waste, and product phase (bottom) directly to separator.
Step 12: Transfer all from separator directly to rotavap at default speed, rinsing separator with ch2cl2 (1 x 30 mL), without flushing tubing after the transfer.
Step 13: Reset liquid handling apparatus with methanol (3 x 10 mL).
Step 14: Reset liquid handling apparatus with ch2cl2 (3 x 10 mL).
Step 15: Evaporate contents of rotavap with default pressure control at temperature 40 °C for 60 min.

```
Step 16: Evaporate contents of rotavap with pressure 1 mbar at temperature
50 °C for 60 min.
```

Blueprint *TmsProtection*:

```
Step 1: Repeat 2 times:
    Add ch2cl2 (20 mL) directly to rotavap at default speed with stirring at
250 RPM.
    Transfer all from rotavap directly to reactor3 at default speed, flushing
tubing after the transfer.
Step 2: Add et3n (3.0 eq.) directly to reactor3 over 5 min with stirring at
250 RPM. Flush liquid handling apparatus with ch2cl2 (3 mL) after addition.
Step 3: Add tmsotf (2.5 eq.) directly to reactor3 over 5 min with stirring
at 250 RPM. Flush liquid handling apparatus with ch2cl2 (3 mL) after addition.
Step 4: Stir reactor3 for 16 h at 250 RPM stopping stirring afterwards.
Step 5*: Clean rotavap with ch2cl2 (1 × 30 mL) without temperature control,
without drying, stirring for 60 s at 150 RPM.
Step 6: Transfer all from reactor3 directly to separator at default speed,
rinsing reactor3 with ch2cl2 (2 x 20 mL), without flushing tubing after the
transfer.
Step 7: Wash contents of separator with nahco3 (2 x 80 mL). Transfer waste
phase (top) to waste, and product phase (bottom) directly to separator.
Step 8: Transfer all from separator directly to rotavap at default speed,
rinsing separator with ch2cl2 (1 x 30 mL), without flushing tubing after the
transfer.
Step 9: Reset liquid handling apparatus with ch2cl2 (3 x 3 mL).
Step 10: Evaporate contents of rotavap with default pressure control at
temperature 40 °C for 60 min.
Step 11: Evaporate contents of rotavap with pressure 1 mbar at temperature
45 °C for 30 min.
```

Purification

The crude product obtained from the automated synthesis was purified by column chromatography (25→100% Et_2O/PE) to afford the title compound as an off-white solid (2.52 g, 7.74 mmol, 77%).

Analytical data

^{1}H NMR (600 MHz, $CDCl_3$) δ 7.49 – 7.43 (m, 2H), 7.37 – 7.33 (m, 2H), 7.32 – 7.17 (m, 5H), 4.03 (t, *J* = 7.2 Hz, 1H), 2.89 – 2.83 (m, 1H), 2.82 – 2.75 (m, 1H), 1.63 – 1.49 (m, 3H), 1.42 – 1.32 (m, 1H), -0.09 (s, 9H).

^{13}C NMR (151 MHz, $CDCl_3$) δ 147.0, 146.0, 128.6, 127.8, 127.8, 127.7, 127.1, 126.9, 83.4, 65.6, 47.4, 27.7, 25.2, 2.4.

The analytical data is in accordance with that previously reported in the literature.(*9*)

Synthesis of racemate

The racemic catalyst was prepared by performing the Grignard addition/Deprotection/Silylation sequence described above manually using 1:1 mixture of *N*-Boc-D-Pro-OMe and *N*-Boc-L-Pro-OMe as the starting material. The product ***rac*-Cat-2** was obtained as an off-white solid (2.70 g, 8.31 mmol, 83%).

(S)-Cat-3
(S)-1,1'-((pyrrolidin-2-yl((trimethylsilyl)oxy)methylene)bis(4,1-phenylene))bis(N,N-dimethylmethanamine)

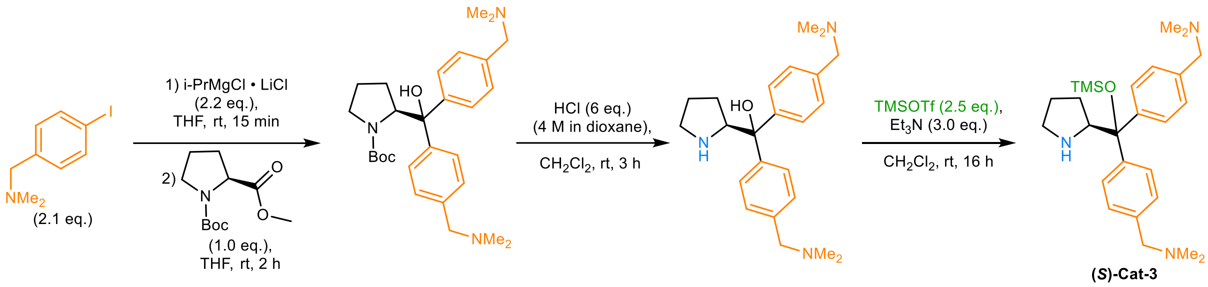

Manual preparations

The system was configured as specified in the graph file `(S)-Cat-3.json` for the reaction.

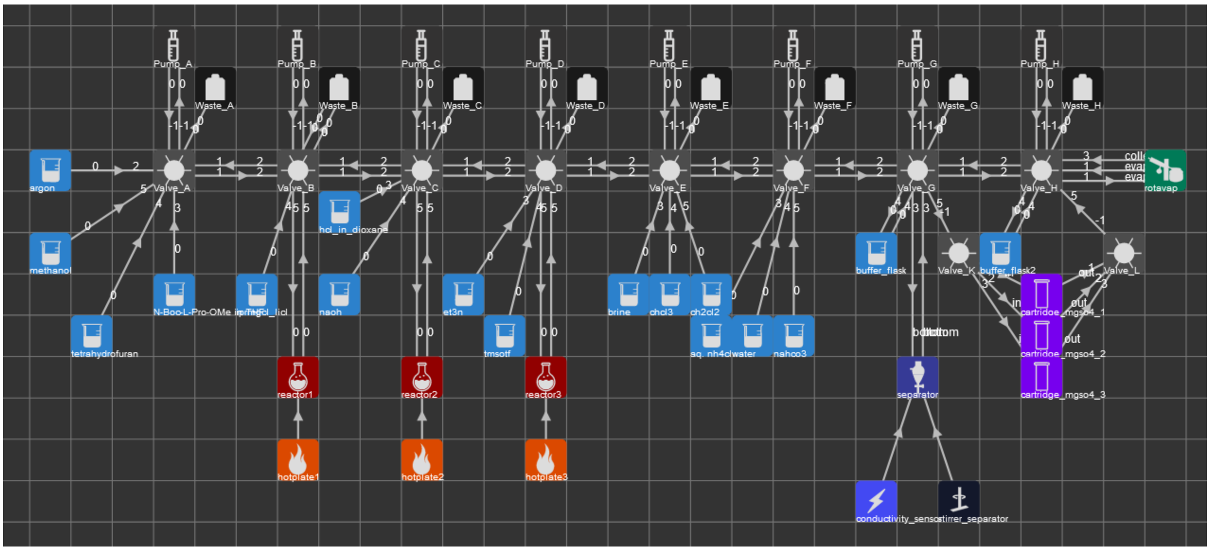

Figure S3: Graphical representation of `(S)-Cat-3.json`

All reagent flasks were connected to a passive (non-controllable) argon supply.

Iodoarene starting material (a low melting solid) was weighed and added into the corresponding reactor flask (reactor1)

Automated synthesis

The synthesis was encoded into three independent Blueprints – one Blueprint per conventional synthetic step. The following is an automated human-readable print out of the encoded steps for each of the blueprints.

Note: The step list below includes the parameters and properties passed into the blueprints for this synthesis and is not representative of a general Blueprint.

Note: Execution of some steps is parallelized. Steps which occur in parallel to previous steps without waiting for them to finish are indicated with an asterisk (*) after the step index.

For general blueprints and full synthesis script refer to synthesis file `(S)-Cat-3.xdl`.

Blueprint *GrignardAddition:*

```
Step 1: Reset liquid handling apparatus with tetrahydrofuran (3 x 3 mL).
Step 2: Add 4-iodobenzyldimethylamine (2.1 eq.) directly to reactor1 at
default speed without stirring. Flush liquid handling apparatus with
tetrahydrofuran (5 mL) after addition.
Step 3: Add iprmgcl_licl (2.2 eq.) directly to reactor1 over 5 min with
stirring at 250 RPM. Flush liquid handling apparatus with tetrahydrofuran (3
mL) after addition.
Step 4: Stir reactor1 for 15 min at 250 RPM stopping stirring afterwards.
Step 5: Add N-Boc-L-Pro-OMe in THF (1.0 eq.) directly to reactor1 at default
speed with stirring at 250 RPM. Flush liquid handling apparatus with
tetrahydrofuran (5 mL) after addition.
Step 6: Stir reactor1 for 3 h at 250 RPM stopping stirring afterwards.
Step 7: Add aq. nh4cl (25 mL) directly to reactor1 at default speed with
stirring at 400 RPM.
Step 8: Add water (15 mL) directly to reactor1 at default speed with stirring
at 400 RPM.
Step 9: Transfer all from reactor1 directly to separator at default speed,
flushing tubing after the transfer.
Step 10: Repeat 2 times:
    Add chcl3 (20 mL) directly to reactor1 at default speed with stirring at
350 RPM.
    Add water (10 mL) directly to reactor1 at default speed with stirring at
350 RPM.
    Transfer all from reactor1 directly to separator at default speed,
flushing tubing after the transfer.
Step 11: Extract contents of separator with chcl3 (2 x 40 mL). Transfer waste
phase (top) to waste, and product phase (bottom) directly to separator.
Step 12: Transfer all from separator directly to rotavap at default speed,
rinsing separator with chcl3 (1 x 30 mL), without flushing tubing after the
transfer.
Step 13: Reset liquid handling apparatus with chcl3 (3 x 3 mL).
Step 14: Evaporate contents of rotavap with default pressure control at
temperature 40 °C for 60 min.
Step 15: Evaporate contents of rotavap with pressure 1 mbar at temperature
40 °C for 15 min.
```

Blueprint *BocDeprotection:*

```
Step 1: Repeat 2 times:
    Dissolve contents of rotavap in ch2cl2 (15 mL) over 5 min, stirring at
280 RPM.
    Transfer 30 mL from rotavap directly to reactor2 at default speed,
flushing tubing after the transfer.
Step 2: Add hcl_in_dioxane (6 eq.) directly to reactor2 at default speed
with stirring at 250 RPM.
Step 3: Stir reactor2 for 3 h at 250 RPM stopping stirring afterwards.
Step 4*: Clean rotavap with ch2cl2 (1 × 30 mL) without temperature control,
without drying, stirring for 60 s at 150 RPM.
```

Step 5: Add naoh (10 eq.) directly to reactor2 at default speed with stirring at 250 RPM.
Step 6: Add water (50 mL) directly to reactor2 at default speed with stirring at 250 RPM.
Step 7: Add ch2cl2 (30 mL) directly to reactor2 at default speed with stirring at 250 RPM.
Step 8: Stir reactor2 for 10 min at 600 RPM stopping stirring afterwards.
Step 9: Transfer all from reactor2 directly to separator at default speed, rinsing reactor2 with ch2cl2 (1 x 30 mL), without flushing tubing after the transfer.
Step 10: Extract contents of separator with ch2cl2 (2 x 40 mL). Transfer waste phase (top) to waste, and product phase (bottom) directly to separator.
Step 11: Wash contents of separator with brine (2 x 40 mL). Transfer waste phase (top) to waste, and product phase (bottom) directly to separator.
Step 12: Transfer all from separator directly to rotavap at default speed, rinsing separator with ch2cl2 (1 x 30 mL), without flushing tubing after the transfer.
Step 13: Reset liquid handling apparatus with methanol (3 x 10 mL).
Step 14: Reset liquid handling apparatus with ch2cl2 (3 x 10 mL).
Step 15: Evaporate contents of rotavap with default pressure control at temperature 40 °C for 2 h.
Step 16: Evaporate contents of rotavap with pressure 1 mbar at temperature 50 °C for 60 min.

Blueprint *TmsProtection*:

Step 1: Repeat 2 times:
    Add ch2cl2 (20 mL) directly to rotavap at default speed with stirring at 250 RPM.
    Transfer all from rotavap directly to reactor3 at default speed, flushing tubing after the transfer.
Step 2: Add et3n (3.0 eq.) directly to reactor3 over 5 min with stirring at 250 RPM. Flush liquid handling apparatus with ch2cl2 (3 mL) after addition.
Step 3: Add tmsotf (2.5 eq.) directly to reactor3 over 5 min with stirring at 250 RPM. Flush liquid handling apparatus with ch2cl2 (3 mL) after addition.
Step 4: Stir reactor3 for 16 h at 250 RPM stopping stirring afterwards.
Step 5*: Clean rotavap with ch2cl2 (1 × 30 mL) without temperature control, without drying, stirring for 60 s at 150 RPM.
Step 6: Transfer all from reactor3 directly to separator at default speed, rinsing reactor3 with ch2cl2 (2 x 20 mL), without flushing tubing after the transfer.
Step 7: Wash contents of separator with nahco3 (2 x 80 mL). Transfer waste phase (top) to waste, and product phase (bottom) directly to separator.
Step 8: Transfer all from separator directly to rotavap at default speed, rinsing separator with ch2cl2 (1 x 30 mL), without flushing tubing after the transfer.
Step 9: Reset liquid handling apparatus with ch2cl2 (3 x 3 mL).
Step 10: Evaporate contents of rotavap with default pressure control at temperature 40 °C for 60 min.
Step 11: Evaporate contents of rotavap with pressure 1 mbar at temperature 45 °C for 30 min.

Purification

The crude product obtained from the automated synthesis was purified by column chromatography (Biotage® Sfär KP-Amino D, 30% Et_2O/PE) to afford the title compound as a highly viscous orange oil (2.1 g, 4.78 mmol, 48%).

Analytical data

^{1}H NMR (600 MHz, $CDCl_3$) δ 7.39 (d, *J* = 8.0 Hz, 2H), 7.29 (d, *J* = 8.0 Hz, 2H), 7.22 – 7.15 (m, 4H), 4.02 (t, *J* = 6.8 Hz, 1H), 3.43 – 3.32 (m, 4H), 2.83 (dd, *J* = 10.0, 6.9 Hz, 1H), 2.73 (td, *J* = 8.2, 6.4, 3.4 Hz, 1H), 2.22 (d, *J* = 2.9 Hz, 12H), 1.69 – 1.61 (m, 1H), 1.60 – 1.49 (m, 3H), 1.37 – 1.26 (m, 1H), -0.12 (s, 9H).
^{13}C NMR (151 MHz, $CDCl_3$) δ 145.7, 144.7, 137.6, 137.5, 128.6, 128.6, 128.5, 127.8, 83.2, 65.6, 64.3, 64.3, 47.3, 45.6, 45.6, 27.7, 25.3, 2.3.

The analytical data is in accordance with that previously reported in the literature.(*10*)

1a (S)-2-isopropyl-5-oxohexanal

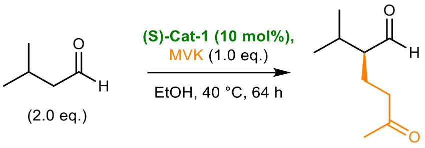

1a

Manual preparations

The system was configured as specified in the graph file `1a.json` for the reaction.

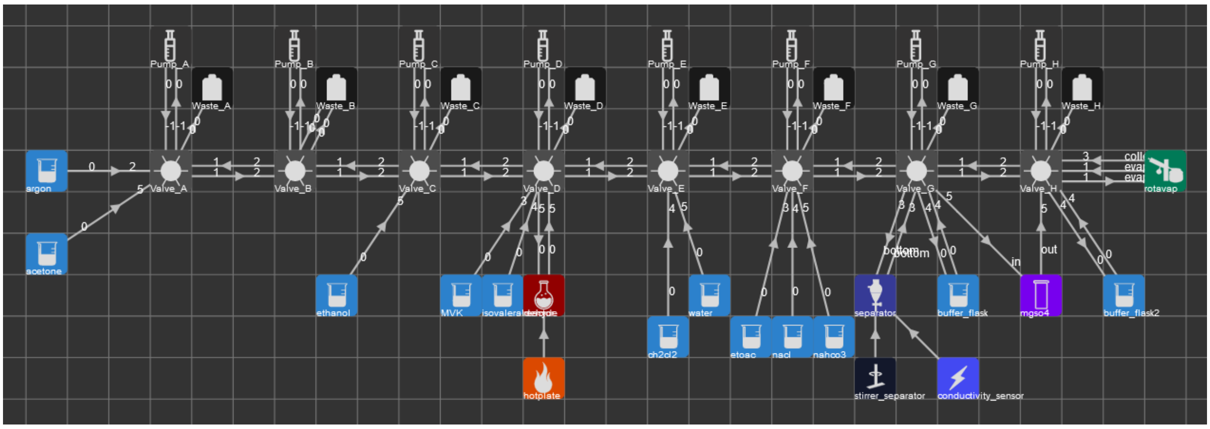

Figure S4: Graphical representation of `1a.json`

Catalyst **(*S*)-Cat-1** was weighed and added into the corresponding reactor flask (reactor).

Automated synthesis

The following is an automated human-readable print out of the encoded steps. For full synthesis script refer to synthesis file **`1a.xdl`**.

```
Step 1: Reset liquid handling apparatus with ethanol (3 x 3 mL).
Step 2: Add (S)-a,a-Bis[3,5-bis(trifluoromethyl)phenyl]-2-pyrrolidinemethanol (1 mmol) directly to reactor at default speed without stirring.
Step 3: Add isovaleraldehyde (20 mmol) directly to reactor at default speed with stirring at 250 RPM. Flush liquid handling apparatus with ethanol (2.5 mL) after addition.
Step 4: Add MVK (10 mmol) directly to reactor at default speed with stirring at 250 RPM. Flush liquid handling apparatus with ethanol (2.5 mL) after addition.
Step 5: Heat/Chill reactor to 45 °C for 64 h with stirring at 250 RPM.
Step 6: Transfer all from reactor directly to rotavap at default speed, rinsing reactor with ethanol (2 x 10 mL), flushing tubing after the transfer.
Step 7: Evaporate contents of rotavap with pressure 50 mbar at temperature 40 °C for 60 min.
Step 8: Shut down the platform.
```

Purification

The crude product obtained from the automated synthesis was purified by column chromatography (0→15% EtOAc/PE) to afford the title compound as a pale-yellow oil (726 mg, 4.65 mmol, 47%, >96:4 er).

Analytical data

^{1}H NMR (600 MHz, $CDCl_3$) δ 9.61 (d, *J* = 2.8 Hz, 1H), 2.50 (ddd, *J* = 17.9, 8.8, 5.7 Hz, 1H), 2.37 (ddd, *J* = 17.8, 8.4, 6.7 Hz, 1H), 2.12 (s, 3H), 2.10 – 1.99 (m, 2H), 1.90 – 1.80 (m, 1H), 1.79 – 1.72 (m, 1H), 1.00 (d, *J* = 6.6 Hz, 3H), 0.97 (d, *J* = 6.7 Hz, 3H).

^{13}C NMR (151 MHz, $CDCl_3$) δ 208.2, 205.5, 57.8, 41.5, 30.2, 28.6, 20.5, 19.7, 19.6.

Enantiomeric ratio was determined by the method of Gellman.(*11*) A small amount (*ca.* 10 µL) of **1a** was added to an NMR tube with 0.7 mL of CD_3CN. To the NMR tube was then added *ca.* 20 µL L-Valine methyl ester*, the tube was rapidly shaken and a ^{1}H NMR spectrum was obtained immediately (number of scans = 4, D1 = 24 s). The enantiomeric ratio was obtained by integration of the imine protons of the two resulting diastereoisomers. δ_H (major) 7.50 (d, *J* = 6.4 Hz), δ_H (minor) 7.46 (d, *J* = 6.7 Hz).

*L-Valine methyl ester was prepared from commercially available L-Valine methyl ester hydrochloride by dissolving the salt in a mixture of CH_2Cl_2/sat. aq. $NaHCO_3$ (1:1), separating the organic extract, drying over $MgSO_4$ and removing the solvent *in vacuo*. The resulting oil was stored in a -20 °C freezer.

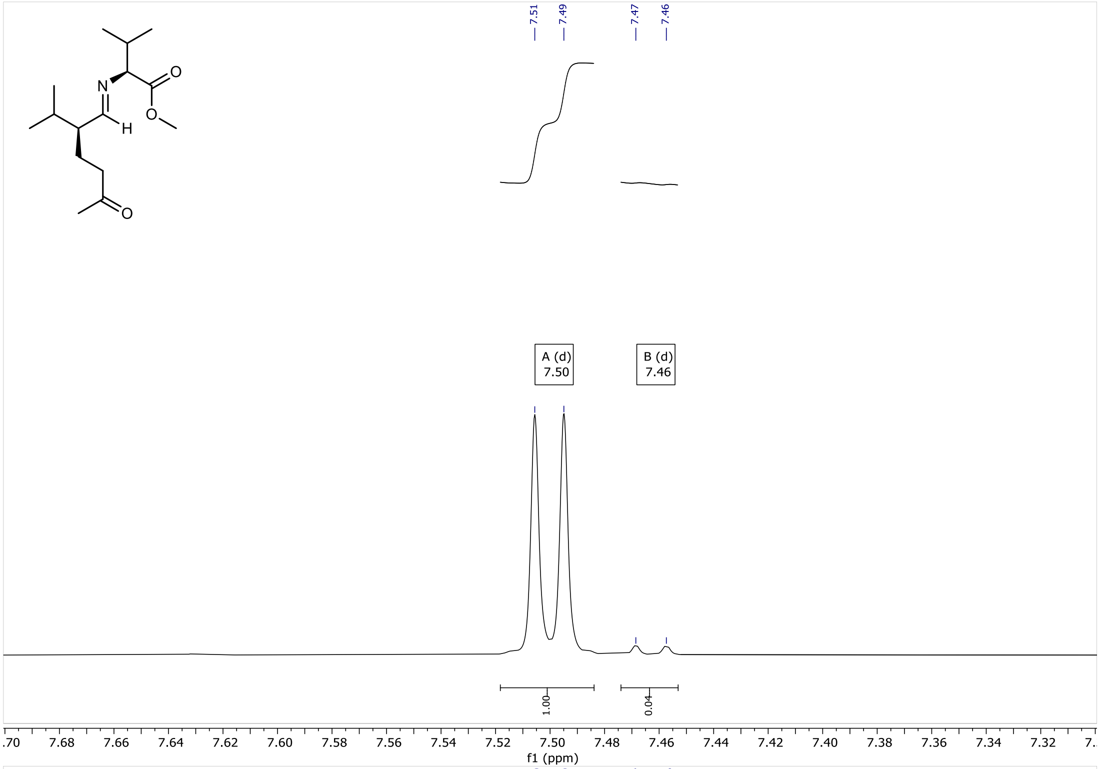

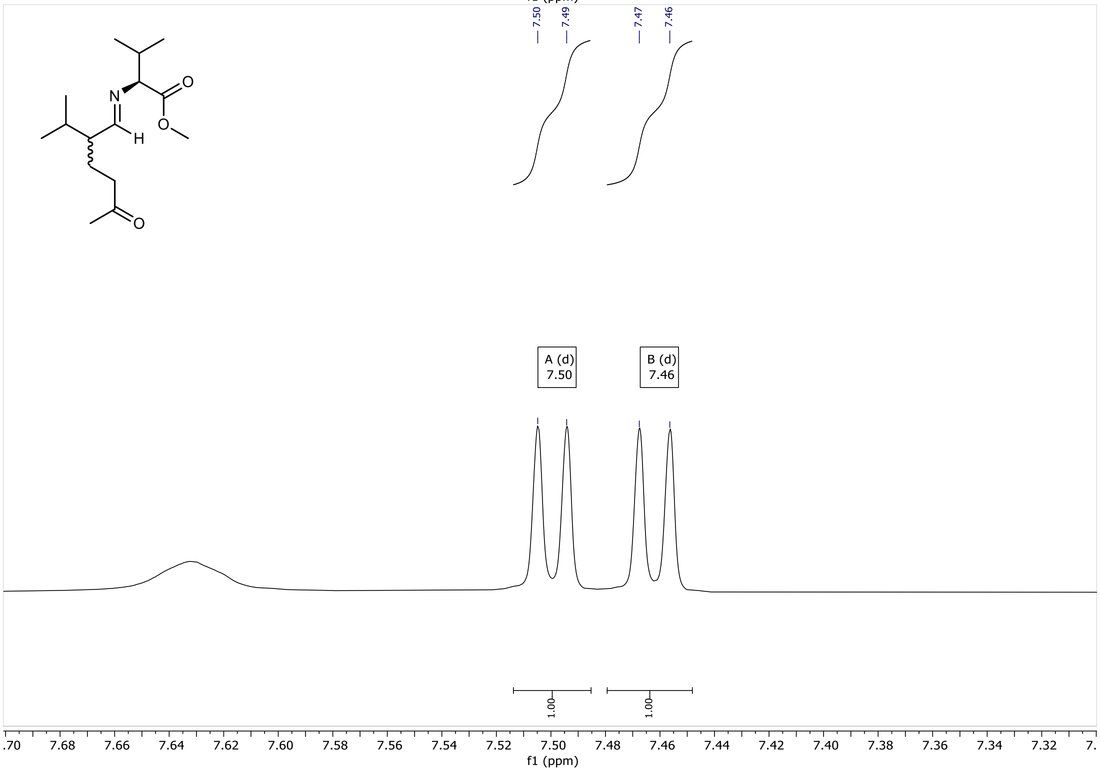

Synthesis of racemate

A racemic sample was prepared by performing the above procedure on 5.0 mmol scale manually using ***rac*-Cat-1** as the catalyst. The product was obtained as a pale-yellow oil (390 mg, 2.50 mmol, 50%).

1b (R)-5-oxo-2-propylhexanal

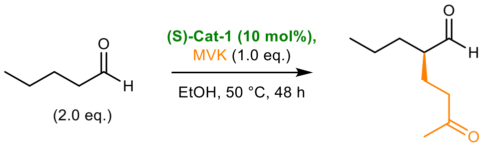

1b

Manual preparations

The system was configured as specified in the graph file `1b.json` for the reaction.

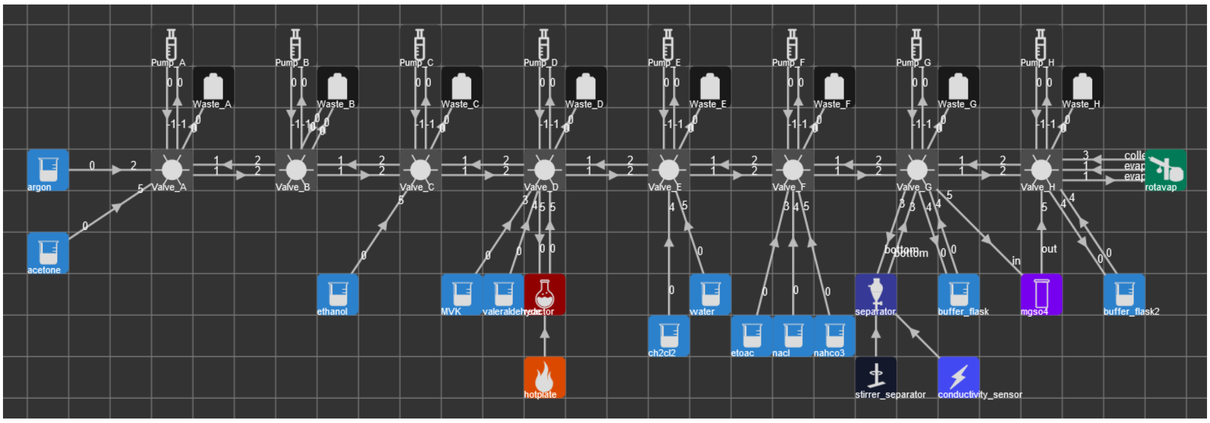

Figure S5: Graphical representation of `1b.json`

Catalyst **(*S*)-Cat-1** was weighed and added into the corresponding reactor flask (reactor).

Automated synthesis

The following is an automated human-readable print out of the encoded steps. For full synthesis script refer to synthesis file `1b.xdl`

```
Step 1: Reset liquid handling apparatus with ethanol (3 x 3 mL).
Step 2: Add (S)-a,a-Bis[3,5-bis(trifluoromethyl)phenyl]-2-pyrrolidinemethanol (1 mmol) directly to reactor at default speed without stirring.
Step 3: Add ethanol (15 mL) directly to reactor at default speed without stirring.
Step 4: Add valeraldehyde (20 mmol) directly to reactor at default speed with stirring at 250 RPM. Flush liquid handling apparatus with ethanol (2.5 mL) after addition.
Step 5: Add MVK (10 mmol) directly to reactor at default speed with stirring at 250 RPM. Flush liquid handling apparatus with ethanol (2.5 mL) after addition.
Step 6: Heat/Chill reactor to 50 °C for 48 h with stirring at 250 RPM.
Step 7: Transfer all from reactor directly to rotavap at default speed, rinsing reactor with ethanol (3 x 10 mL), flushing tubing after the transfer.
Step 8: Evaporate contents of rotavap with pressure 50 mbar at temperature 40 °C for 60 min.
Step 9: Shut down the platform.
```

Purification

The crude product obtained from the automated synthesis was purified by column chromatography (0→15% EtOAc/PE) to afford the title compound as a pale-yellow oil (658 mg, 4.22 mmol, 42%, 96:4 er).

Analytical data

^{1}H NMR (600 MHz, $CDCl_3$) δ 9.56 (d, *J* = 2.8 Hz, 1H), 2.52 – 2.33 (m, 2H), 2.26 (ddtd, *J* = 10.2, 7.3, 5.1, 2.5 Hz, 1H), 2.13 (s, 3H), 1.92 – 1.82 (m, 1H), 1.82 – 1.70 (m, 1H), 1.69 – 1.59 (m, 1H), 1.47 – 1.30 (m, 3H), 0.92 (t, *J* = 7.3 Hz, 3H).

^{13}C NMR (151 MHz, $CDCl_3$) δ 208.1, 205.0, 51.2, 40.9, 31.4, 30.2, 22.5, 20.4, 14.3.

Enantiomeric ratio was determined by the method of Gellman.(*11*) A small amount (*ca.* 10 µL) of **1b** was added to an NMR tube with 0.7 mL of CD_3CN. To the NMR tube was then added *ca.* 20 µL L-Valine methyl ester*, the tube rapidly shaken and a ^{1}H NMR spectrum was obtained immediately (number of scans = 4, D1 = 24 s). The enantiomeric ratio was obtained by integration of the imine protons of the two resulting diastereoisomers. δ_H (major) 7.43 (d, *J* = 6.1 Hz), δ_H (minor) 7.38 (d, *J* = 6.5 Hz).

*L-Valine methyl ester was prepared from commercially available L-Valine methyl ester hydrochloride by dissolving the salt in a mixture of CH_2Cl_2/sat. aq. $NaHCO_3$ (1:1), separating the organic extract, drying over $MgSO_4$ and removing the solvent *in vacuo*. The resulting oil was stored in a -20 °C freezer.

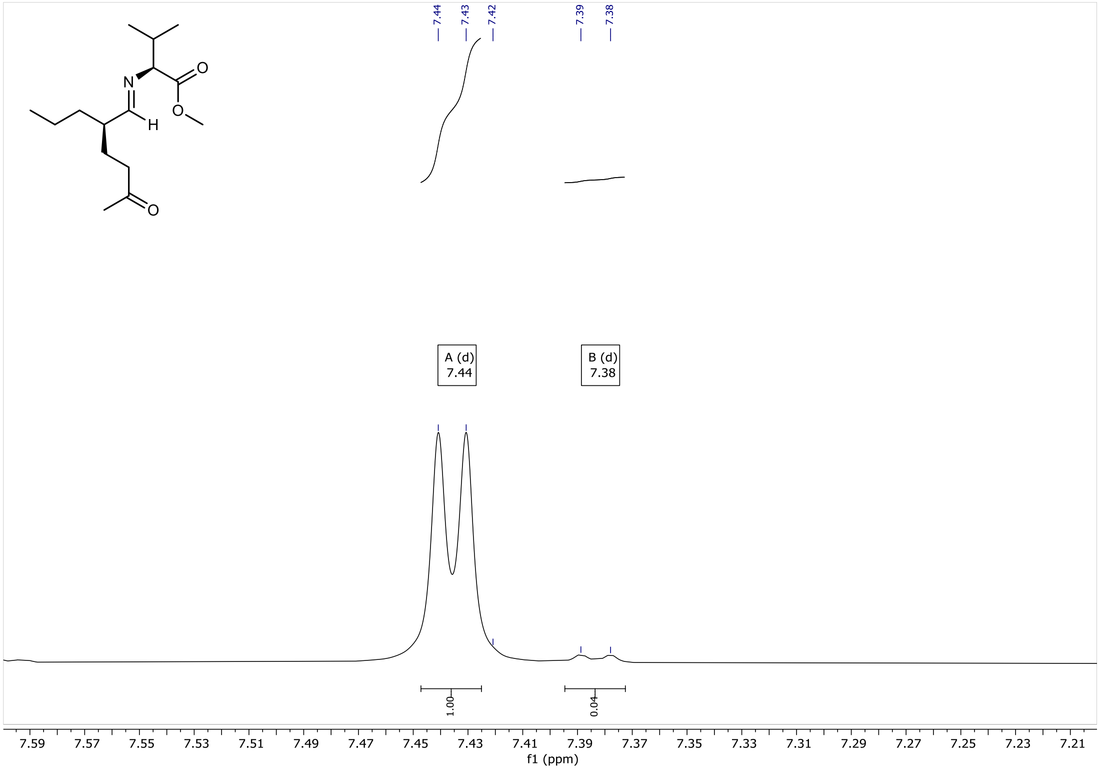

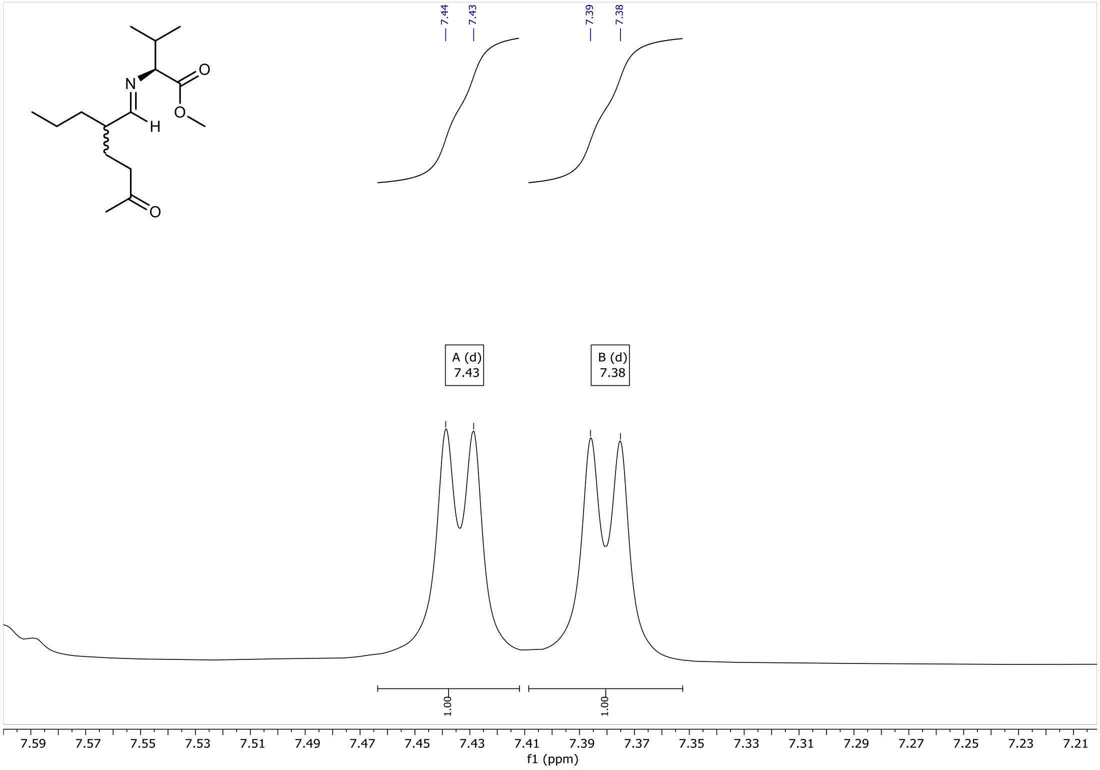

Synthesis of racemate

A racemic sample was prepared by performing the above procedure on 5.0 mmol scale manually using ***rac*-Cat-1** as the catalyst. The product was obtained as a pale-yellow oil (430 mg, 2.76 mmol, 55%).

2 (S)-2-chloro-3-phenylpropan-1-ol

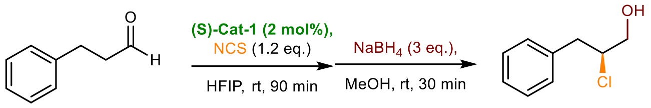

2

Manual preparations

The system was configured as specified in the graph file `2.json` for the reaction.

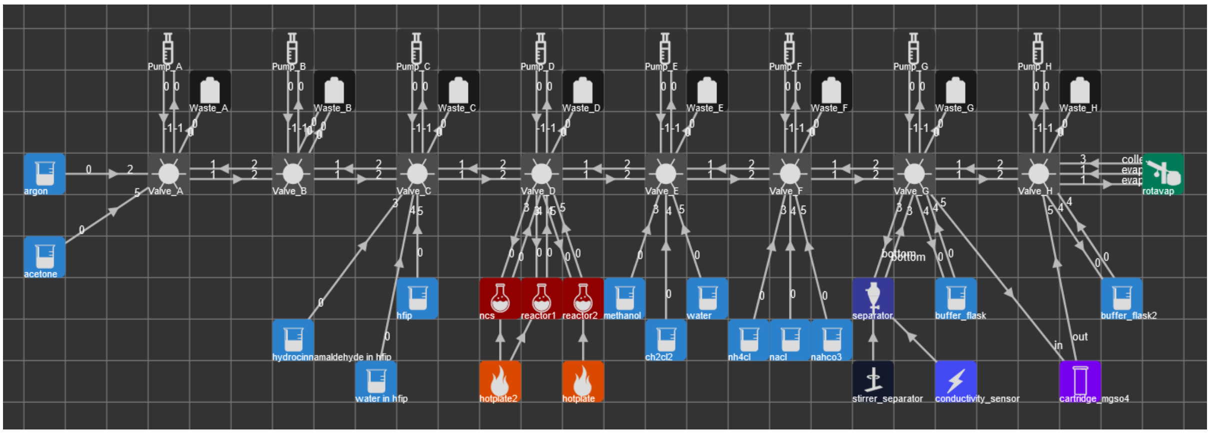

Figure S6: Graphical representation of `2.json`

Catalyst **(*S*)-Cat-1** was weighed and added into the corresponding reactor flask (reactor1).

N-chlorosuccinimide was weighed and added into the corresponding reactor flask (ncs).

Sodium borohydride was weighed and added into the corresponding reactor flask (reactor2).

Automated synthesis

The following is an automated human-readable print out of the encoded steps. For full synthesis script refer to synthesis file `2.xdl`

```
Step 1: Add (S)-a,a-Bis[3,5-bis(trifluoromethyl)phenyl]-2-pyrrolidinemethanol (0.2 mmol) directly to reactor1 at default speed without stirring.
Step 2: Add hydrocinnamaldehyde in hfip (10 mmol) directly to reactor1 at default speed with stirring at 250 RPM. Flush liquid handling apparatus with hfip (1.5 mL) after addition.
Step 3: Add water in hfip (10 mmol) directly to reactor1 at default speed with stirring at 250 RPM. Flush liquid handling apparatus with hfip (1.5 mL) after addition.
Step 4: Add n-chlorosuccinimide (12 mmol) directly to ncs at default speed without stirring.
Step 5: Add hfip (12.5 mL) directly to ncs at default speed with stirring at 250 RPM.
Step 6: Transfer 17.5 mL from ncs directly to reactor1 over 2.5 h, without flushing tubing after the transfer.
```

```
Step 7: Add methanol (30 mL) directly to reactor1 at default speed with stirring at 250 RPM.
Step 8: Add sodium borohydride (40 mmol) directly to reactor2 at default speed without stirring.
Step 9: Transfer all from reactor1 directly to reactor2 at default speed, rinsing reactor1 with methanol (1 x 10 mL), flushing tubing after the transfer.
Step 10: Stir reactor2 for 30 min at 250 RPM stopping stirring afterwards.
Step 11: Add nh4cl (30 mL) directly to reactor2 at default speed with stirring at 250 RPM.
Step 12: Add water (30 mL) directly to reactor2 at default speed with stirring at 250 RPM.
Step 13: Add ch2cl2 (30 mL) directly to reactor2 at default speed with stirring at 250 RPM.
Step 14: Transfer all from reactor2 directly to separator at default speed, rinsing reactor2 with ch2cl2 (2 x 20 mL), flushing tubing after the transfer.
Step 15: Add nacl (40 mL) directly to separator at default speed with stirring at 250 RPM.
Step 16: Extract contents of separator with ch2cl2 (2 x 50 mL). Transfer waste phase (top) to waste, and product phase (bottom) directly to separator.
Step 17: Wash contents of separator with nacl (1 x 50 mL). Transfer waste phase (top) to waste, and product phase (bottom) directly to separator.
Step 18: Transfer all from separator through mgso4 to rotavap at default speed, rinsing separator with ch2cl2 (1 x 40 mL), without flushing tubing after the transfer.
Step 19: Reset liquid handling apparatus with ch2cl2 (3 x 3 mL).
Step 20: Evaporate contents of rotavap with pressure 50 mbar at temperature 40 °C for 60 min.
Step 21: Evaporate contents of rotavap with pressure 1 mbar at temperature 45 °C for 15 min.
Step 22: Shut down the platform.
```

Purification

The crude product obtained from the automated synthesis was purified by column chromatography (10→25% EtOAc/PE) to afford the title compound as a yellow oil (1.17 g, 6.90 mmol, 69%, 84:16 *er*).

Analytical data

^{1}H NMR (600 MHz, $CDCl_3$) δ 7.33 (tt, *J* = 7.8, 1.3 Hz, 2H), 7.30 – 7.19 (m, 3H), 4.23 (tdd, *J* = 7.3, 6.2, 3.6 Hz, 1H), 3.81 (ddd, *J* = 12.1, 7.3, 3.6 Hz, 1H), 3.69 (dt, *J* = 12.2, 6.2 Hz, 1H), 3.14 (dd, *J* = 14.1, 7.0 Hz, 1H), 3.06 (dd, *J* = 14.1, 7.4 Hz, 1H), 1.98 (dd, *J* = 7.3, 6.3 Hz, 1H).

^{13}C NMR (151 MHz, $CDCl_3$) δ 137.3, 129.5, 128.8, 127.2, 66.1, 65.1, 65.1, 40.9.

The analytical data is in accordance with that previously reported in the literature.(*12*)

Enantiomeric ratio was determined by chiral HPLC: Chiral Art Cellulose-C column, hexane:isopropanol = 98:02, 0.7 mL/min flow rate, 208 nm detection wavelength. t_R (major) = 46.7 min, t_R (minor) = 49.7 min.

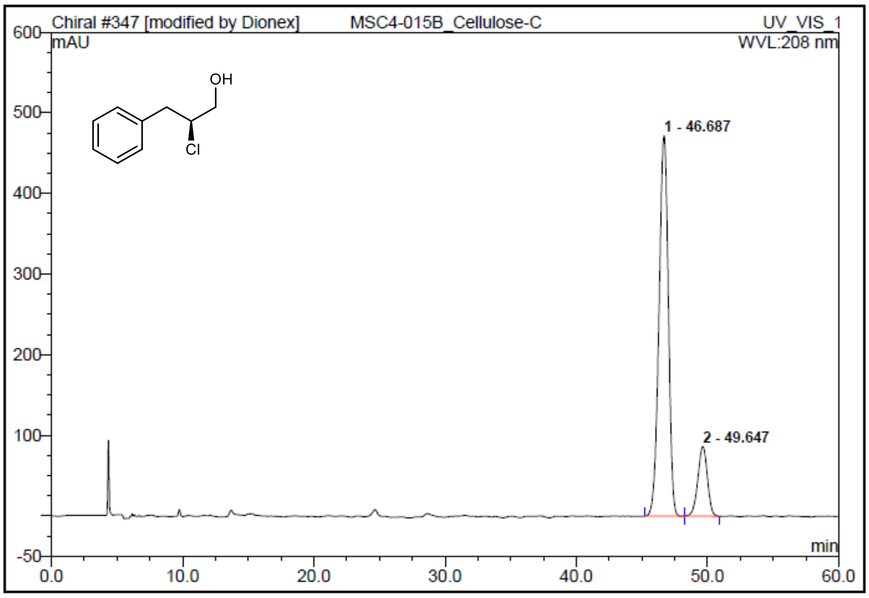

No.	Ret.Time min	Peak Name	Height mAU	Area mAU*min	Rel.Area %	Amount	Type
1	46.69	n.a.	471.428	387.435	84.21	n.a.	BMb*
2	49.65	n.a.	86.291	72.645	15.79	n.a.	bMB*
Total:			557.719	460.080	100.00	0.000	

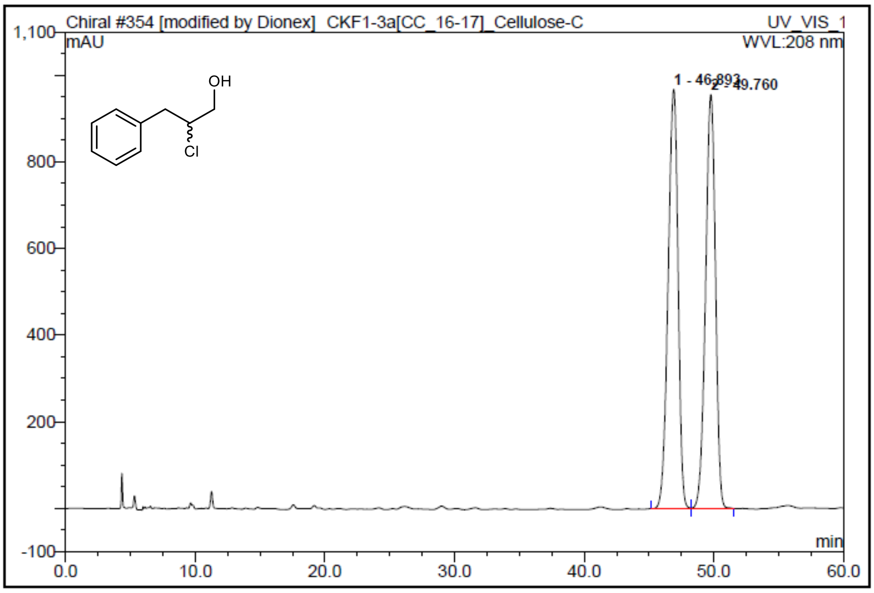

No.	Ret.Time min	Peak Name	Height mAU	Area mAU*min	Rel.Area %	Amount	Type
1	46.89	n.a.	968.143	859.878	50.38	n.a.	BM *
2	49.76	n.a.	955.644	846.812	49.62	n.a.	MB*
Total:			1923.786	1706.690	100.00	0.000	

Synthesis of racemate

A racemic sample was prepared by performing the above procedure on 2.0 mmol scale manually using ***rac*-Cat-1** as the catalyst. The product was obtained as a colourless oil (128 mg, 0.75 mmol, 37%).

4 ((2R,3R)-3-phenyloxiran-2-yl)methanol

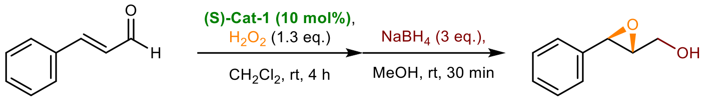

4

Manual preparations

The system was configured as specified in the graph file **4.json** for the reaction.

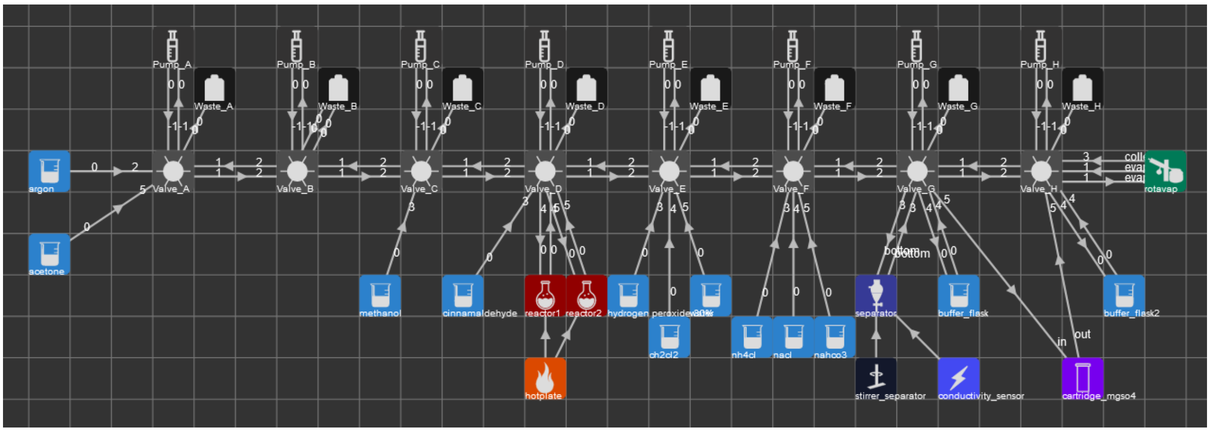

Figure S7: Graphical representation of **4.json**

Catalyst **(*S*)-Cat-1** was weighed and added into the corresponding reactor flask (reactor1).
Sodium borohydride was weighed into the corresponding reactor flask (reactor2).

Automated synthesis

The following is an automated human-readable print out of the encoded steps. For full synthesis script refer to synthesis file **4.xdl**

```
Step 1: Reset liquid handling apparatus with ch2cl2 (3 x 3 mL).
Step 2: Add (S)-a,a-Bis[3,5-bis(trifluoromethyl)phenyl]-2-pyrrolidinemethanol (598 mg) directly to reactor1 at default speed without stirring.
Step 3: Add cinnamaldehyde (1.26 mL) directly to reactor1 at default speed with stirring at 250 RPM. Flush liquid handling apparatus with ch2cl2 (5 mL) after addition.
Step 4: Add ch2cl2 (15 mL) directly to reactor1 at default speed with stirring at 250 RPM.
Step 5: Add hydrogen peroxide 30% (1.33 mL) directly to reactor1 at default speed with stirring at 250 RPM. Flush liquid handling apparatus with water (2 mL) after addition.
Step 6: Stir reactor1 for 4 h at 250 RPM stopping stirring afterwards.
Step 7: Add sodium borohydride (1.52 g) directly to reactor2 at default speed without stirring.
Step 8: Add methanol (15 mL) directly to reactor1 at default speed with stirring at 250 RPM.
```

```
Step 9: Transfer all from reactor1 directly to reactor2 at default speed,
rinsing reactor1 with methanol (1 x 10 mL), flushing tubing after the
transfer.
Step 10: Stir reactor2 for 30 min at 250 RPM stopping stirring afterwards.
Step 11: Transfer all from reactor2 directly to separator at default speed,
rinsing reactor2 with ch2cl2 (2 x 15 mL), flushing tubing after the transfer.
Step 12: Add nh4cl (20 mL) directly to separator at default speed with
stirring at 250 RPM.
Step 13: Add water (50 mL) directly to separator at default speed with
stirring at 250 RPM.
Step 14: Stir separator for 5 min at 250 RPM stopping stirring afterwards.
Step 15: Extract contents of separator with ch2cl2 (2 x 40 mL). Transfer
waste phase (top) to waste, and product phase (bottom) directly to separator.
Step 16: Wash contents of separator with nacl (1 x 40 mL). Transfer waste
phase (top) to waste, and product phase (bottom) through mgso4 to rotavap.
Step 17: Transfer all from separator through mgso4 to rotavap at default
speed, rinsing separator with ch2cl2 (1 x 40 mL), without flushing tubing
after the transfer.
Step 18: Evaporate contents of rotavap with pressure 50 mbar at temperature
40 °C for 60 min.
Step 19: Shut down the platform.
```

Purification

The crude product obtained from the automated synthesis was purified by column chromatography (15→30% EtOAc/PE) to afford the title compound as a white solid (804 mg, 5.67 mmol, 57%, 98.5:1.5 *er*, 91:9 *dr*).

Analytical data

Anti (major)

^{1}H NMR (600 MHz, $CDCl_3$) δ 7.39 – 7.27 (m, 5H), 4.05 (ddd, *J* = 12.8, 5.2, 2.4 Hz, 1H), 3.93 (d, *J* = 2.1 Hz, 1H), 3.81 (ddd, *J* = 12.7, 7.8, 3.8 Hz, 1H), 3.23 (dt, *J* = 4.2, 2.3 Hz, 1H), 1.78 (dd, *J* = 7.8, 5.3 Hz, 1H),

^{13}C NMR (151 MHz, $CDCl_3$) δ 136.9, 128.7, 128.5, 125.9, 62.5, 61.4, 55.7.

Syn (minor)

^{1}H NMR (600 MHz, $CDCl_3$) δ 7.39 – 7.27 (m, 5H), 4.20 (d, *J* = 3.9 Hz, 1H), 3.61 – 3.53 (m, 1H), 3.51 – 3.42 (m, 2H), 1.45 (dd, *J* = 7.3, 4.7 Hz, 1H).

^{13}C NMR (151 MHz, $CDCl_3$) δ 134.9, 128.5, 128.1, 126.4, 60.8, 58.7, 57.3.

The analytical data is in accordance with that previously reported in the literature.(*13*)

Enantiomeric ratio was determined by chiral HPLC: Daicel Chiralpak IB N-5 column, hexane:isopropanol = 95:05, 1 mL/min flow rate, 215 nm detection wavelength. t_R (major, *anti*) = 16.0 min, t_R (minor, *anti*) = 14.8 min, t_R (*syn*) = 9.5 min, 12.1 min.

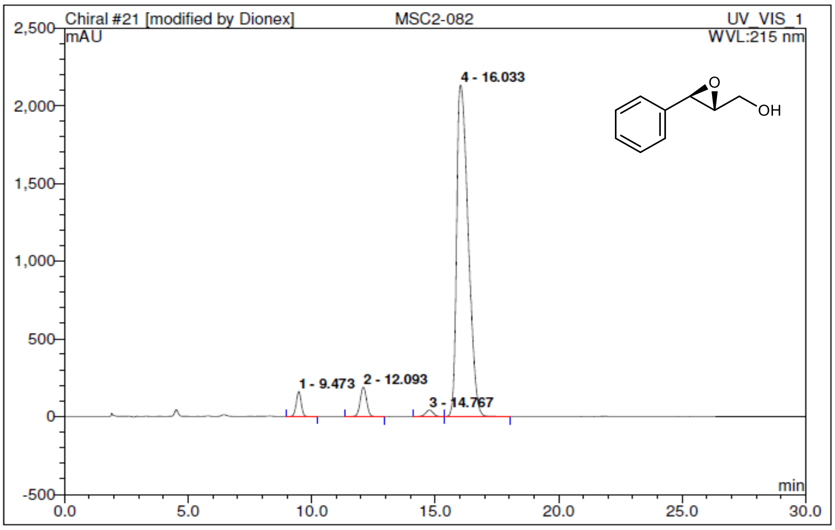

No.	Ret.Time min	Peak Name	Height mAU	Area mAU*min	Rel.Area %	Amount	Type
1	9.47	n.a.	160.795	37.901	3.01	n.a.	BMB
2	12.09	n.a.	188.482	56.520	4.48	n.a.	BMB
3	14.77	n.a.	41.924	14.889	1.18	n.a.	BMb*
4	16.03	n.a.	2132.564	1151.598	91.33	n.a.	bMB*
Total:			2523.765	1260.909	100.00	0.000	

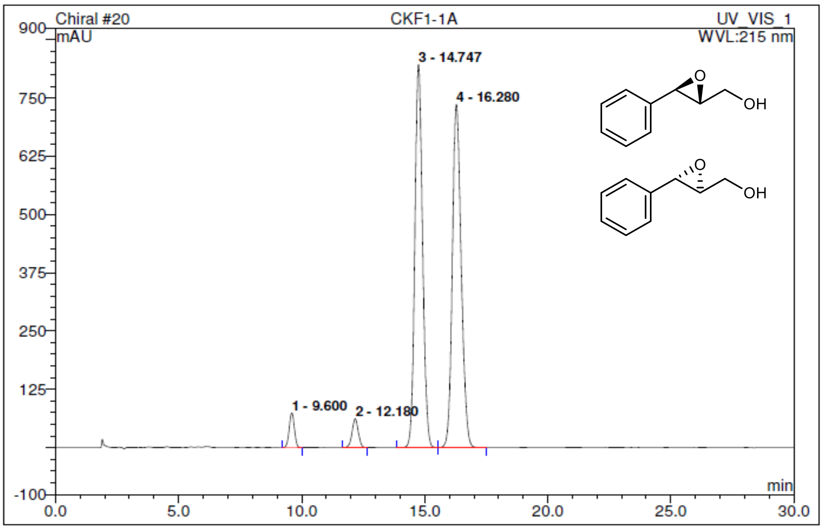

No.	Ret.Time min	Peak Name	Height mAU	Area mAU*min	Rel.Area %	Amount	Type
1	9.60	n.a.	74.304	18.010	2.80	n.a.	BMB
2	12.18	n.a.	61.639	18.191	2.83	n.a.	BMB
3	14.75	n.a.	821.689	299.247	46.59	n.a.	BM
4	16.28	n.a.	736.222	306.782	47.77	n.a.	MB
Total:			1693.854	642.230	100.00	0.000	

Synthesis of racemate

A racemic sample was prepared by performing the above procedure on 2.0 mmol scale manually using ***rac*-Cat-1** as the catalyst. The product was obtained as a pale-yellow oil (199 mg, 1.33 mmol, 66%, 94:6 *dr*).

5 Methyl (S)-3-phenyl-4-(pyridin-2-yl)butanoate

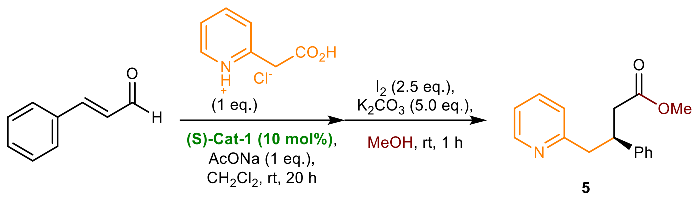

Manual preparations

The system was configured as specified in the graph file `5.json` for the reaction.

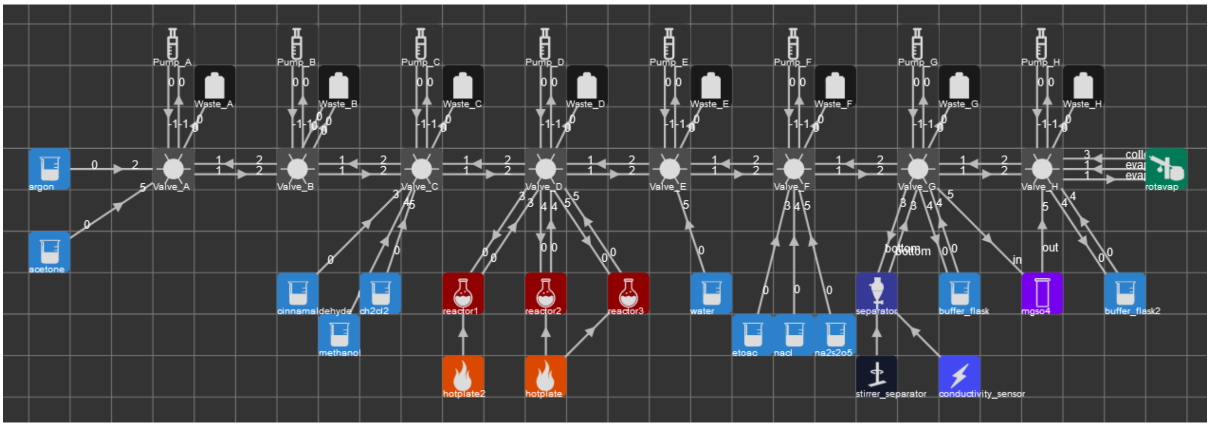

Figure S8: Graphical representation of `5.json`

Iodine was weighed and added into the corresponding reactor flask (reactor1).

Catalyst **(*S*)-Cat-1,** sodium acetate, 2-pyridylacetic acid hydrochloride were weighed and added into the corresponding reactor flask (reactor2).

Potassium carbonate was weighed and added into the corresponding reactor flask (reactor3).

Automated synthesis

The following is an automated human-readable print out of the encoded steps. For full synthesis script refer to synthesis file `5.xdl`

```
Step 1: Reset liquid handling apparatus with ch2cl2 (3 x 3 mL).
Step 2: Add (S)-a,a-Bis[3,5-bis(trifluoromethyl)phenyl]-2-pyrrolidinemethanol (0.5 mmol) directly to reactor2 at default speed without stirring.
Step 3: Add 2-pyridylacetic acid hydrochloride (5.0 mmol) directly to reactor2 at default speed without stirring.
Step 4: Add sodium acetate (5.0 mmol) directly to reactor2 at default speed without stirring.
Step 5: Add k2co3 (25.0 mmol) directly to reactor3 at default speed without stirring.
Step 6: Add iodine (12.5 mmol) directly to reactor1 at default speed without stirring.
Step 7: Add ch2cl2 (5 mL) directly to reactor2 at default speed with stirring at 250 RPM.
```

```
Step 8: Add cinnamaldehyde (5.0 mmol) directly to reactor2 at default speed
with stirring at 250 RPM. Flush liquid handling apparatus with ch2cl2 (5 mL)
after addition.
Step 9: Stir reactor2 for 20 h at 250 RPM stopping stirring afterwards.
Step 10: Add methanol (20 mL) directly to reactor1 at default speed with
stirring at 250 RPM.
Step 11: Stir reactor1 for 5 min at 250 RPM stopping stirring afterwards.
Step 12: Transfer all from reactor2 directly to reactor3 at default speed,
rinsing reactor2 with methanol (1 x 10 mL), without flushing tubing after
the transfer.
Step 13: Transfer all from reactor1 directly to reactor3 at default speed,
rinsing reactor1 with methanol (1 x 5 mL), flushing tubing after the transfer.
Step 14: Stir reactor3 for 60 min at 250 RPM stopping stirring afterwards.
Step 15: Transfer all from reactor3 directly to separator at default speed,
without flushing tubing after the transfer.
Step 16: Add water (20 mL) directly to reactor3 at default speed without
stirring.
Step 17: Add etoac (20 mL) directly to reactor3 at default speed without
stirring.
Step 18: Transfer all from reactor3 directly to separator at default speed,
without flushing tubing after the transfer.
Step 19: Add na2s2o5 (50 mL) directly to separator at default speed without
stirring.
Step 20: Extract contents of separator with etoac (3 x 50 mL). Transfer waste
phase (bottom) to waste, and product phase (top) directly to separator.
Step 21: Wash contents of separator with water (1 x 50 mL). Transfer waste
phase (bottom) to waste, and product phase (top) directly to separator.
Step 22: Wash contents of separator with nacl (1 x 50 mL). Transfer waste
phase (bottom) to waste, and product phase (top) through mgso4 to rotavap.
Step 23: Evaporate contents of rotavap with pressure 20 mbar at temperature
40 °C for 60 min.
Step 24: Shut down the platform.
```

Purification

The crude product obtained from the automated synthesis was purified by column chromatography (30→40% EtOAc/PE) to afford the title compound as an orange oil (1.07 g, 4.20 mmol, 84%, 98.5:1.5 *er*).

Analytical data

^{1}H NMR (600 MHz, $CDCl_3$) δ 8.52 (ddd, *J* = 4.9, 1.9, 1.0 Hz, 1H), 7.49 (td, *J* = 7.6, 1.9 Hz, 1H), 7.25 – 7.23 (m, 2H), 7.19 – 7.15 (m, 3H), 7.07 (ddd, *J* = 7.6, 4.9, 1.2 Hz, 1H), 6.96 (dt, *J* = 7.7, 1.1 Hz, 1H), 3.73 – 3.65 (m, 1H), 3.51 (s, 3H), 3.16 – 3.11 (m, 1H), 3.11 – 3.07 (m, 1H), 2.73 (dd, *J* = 15.5, 6.2 Hz, 1H), 2.68 (dd, *J* = 15.5, 8.8 Hz, 1H).

^{13}C NMR (151 MHz, $CDCl_3$) δ 172.7, 159.8, 149.5, 143.5, 136.3, 128.6, 127.6, 126.8, 123.9, 121.5, 51.6, 45.2, 42.6, 40.5.

The analytical data is in accordance with that previously reported in the literature.(*14*)

Enantiomeric ratio was determined by chiral HPLC: Daicel Chiralpak IB N-5 column, hexane:isopropanol = 95:05, 1 mL/min flow rate, 215 nm detection wavelength. t_R (major) = 53.6 min, t_R (minor) = 60.5 min.

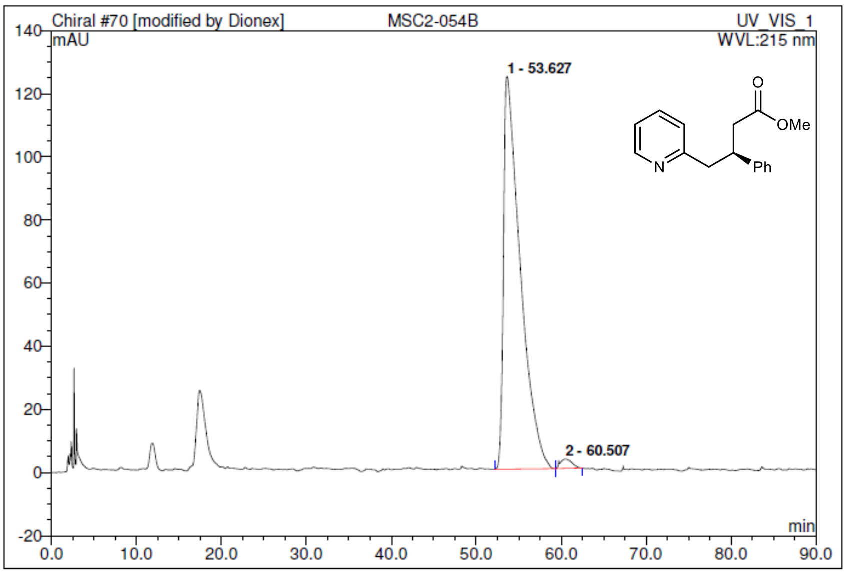

No.	Ret.Time min	Peak Name	Height mAU	Area mAU*min	Rel.Area %	Amount	Type
1	53.63	n.a.	124.450	284.073	98.34	n.a.	BMB
2	60.51	n.a.	3.016	4.784	1.66	n.a.	BMB*
Total:			127.466	288.858	100.00	0.000	

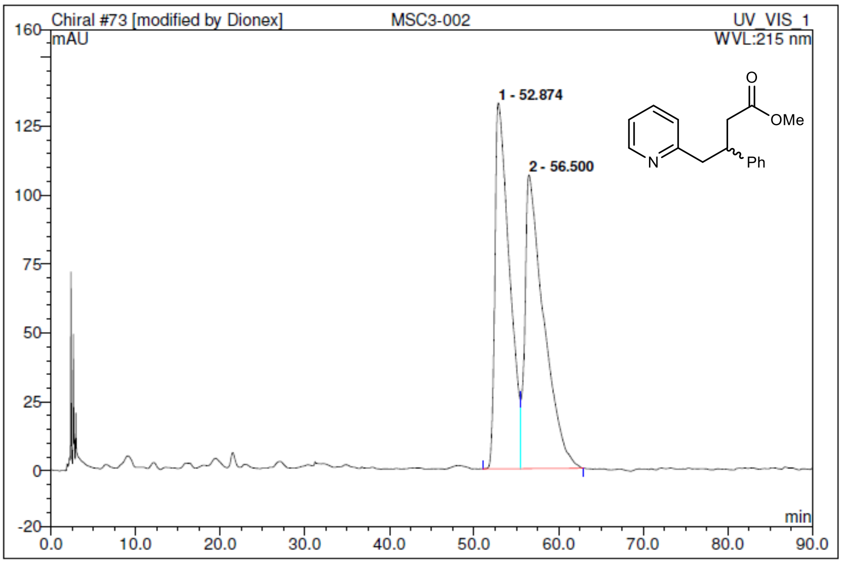

No.	Ret.Time min	Peak Name	Height mAU	Area mAU*min	Rel.Area %	Amount	Type
1	52.87	n.a.	132.707	261.775	48.66	n.a.	BM
2	56.50	n.a.	106.496	276.202	51.34	n.a.	MB
Total:			239.203	537.977	100.00	0.000	

Synthesis of racemate

A racemic sample was prepared by performing the above procedure on 5.0 mmol scale manually using ***rac*-Cat-1** as the catalyst. The product was obtained as an orange oil (1.19 g, 4.65 mmol, 93%).

6 (S)-4-nitro-3-phenylbutanal

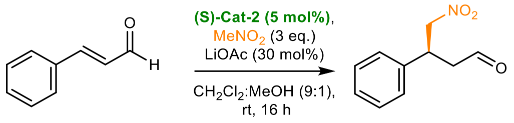

6

Manual preparations

The system was configured as specified in the graph file `6.json` for the reaction.

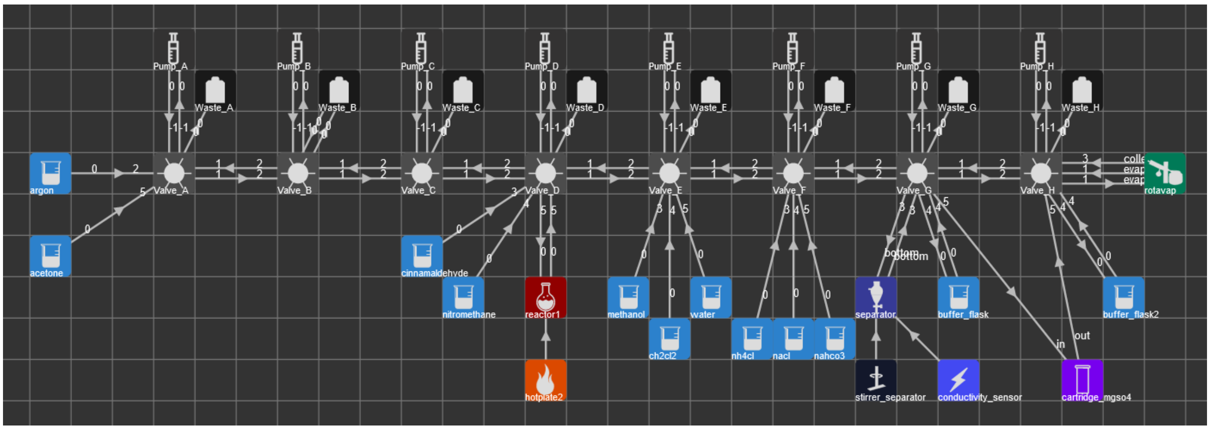

Figure S9: Graphical representation of `6.json`

Catalyst **(*S*)-Cat-2** and benzoic acid were weighed and added into the corresponding reactor flask (reactor).

Automated synthesis

The following is an automated human-readable print out of the encoded steps. For full synthesis script refer to synthesis file `6.xdl`.

```
Step 1: Add (S)-a,a-Bisphenyl-2-pyrrolidinemethanol (0.5 mmol) directly to reactor1 at default speed without stirring.
Step 2: Add lithium acetate (3 mmol) directly to reactor1 at default speed without stirring.
Step 3: Add methanol (2 mL) directly to reactor1 at default speed with stirring at 250 RPM.
Step 4: Add ch2cl2 (10 mL) directly to reactor1 at default speed with stirring at 250 RPM.
Step 5: Add cinnamaldehyde (10 mmol) directly to reactor1 at default speed with stirring at 250 RPM. Flush liquid handling apparatus with ch2cl2 (4 mL) after addition.
Step 6: Add nitromethane (30 mmol) directly to reactor1 at default speed with stirring at 250 RPM. Flush liquid handling apparatus with ch2cl2 (4 mL) after addition.
Step 7: Stir reactor1 for 16 h at 250 RPM stopping stirring afterwards.
Step 8: Add water (50 mL) directly to reactor1 at default speed with stirring at 250 RPM.
Step 9: Transfer all from reactor1 directly to separator at default speed, rinsing reactor1 with ch2cl2 (1 x 20 mL), flushing tubing after the transfer.
Step 10: Extract contents of separator with ch2cl2 (2 x 40 mL). Transfer waste phase (top) to waste, and product phase (bottom) directly to separator.
```

```
Step 11: Transfer all from separator through mgso4 to rotavap at default
speed, rinsing separator with ch2cl2 (1 x 40 mL), without flushing tubing
after the transfer.
Step 12: Reset liquid handling apparatus with ch2cl2 (3 x 3 mL).
Step 13: Evaporate contents of rotavap with pressure 50 mbar at temperature
40 °C for 60 min.
Step 14: Evaporate contents of rotavap with pressure 1 mbar at temperature
40 °C for 15 min.
Step 15: Shut down the platform.
```

Purification

The crude product obtained from the automated synthesis was purified by column chromatography (10→25% EtOAc/PE) to afford the title compound as a yellow oil, which solidified in a -20 °C freezer (1.55 g, 8.05 mmol, 81%, >98.5:1.5 er).

Analytical data

^{1}H NMR (600 MHz, $CDCl_3$) δ 9.71 (t, *J* = 1.1 Hz, 1H), 7.38 – 7.32 (m, 2H), 7.32 – 7.28 (m, 1H), 7.25 – 7.22 (m, 2H), 4.68 (dd, *J* = 12.5, 7.2 Hz, 1H), 4.62 (dd, *J* = 12.6, 7.6 Hz, 1H), 4.08 (p, *J* = 7.2 Hz, 1H), 3.00 – 2.90 (m, 2H).

^{13}C NMR (151 MHz, $CDCl_3$) δ 198.9, 138.3, 129.4, 128.3, 127.6, 79.6, 46.6, 38.2.

The analytical data is in accordance with that previously reported in the literature.(*15*)

Enantiomeric ratio was determined by chiral HPLC: Daicel Chiralpak IB N-5 column, hexane:isopropanol = 90:10, 1 mL/min flow rate, 215 nm detection wavelength. t_R (major) = 24.8 min, t_R (minor) = 26.6 min.

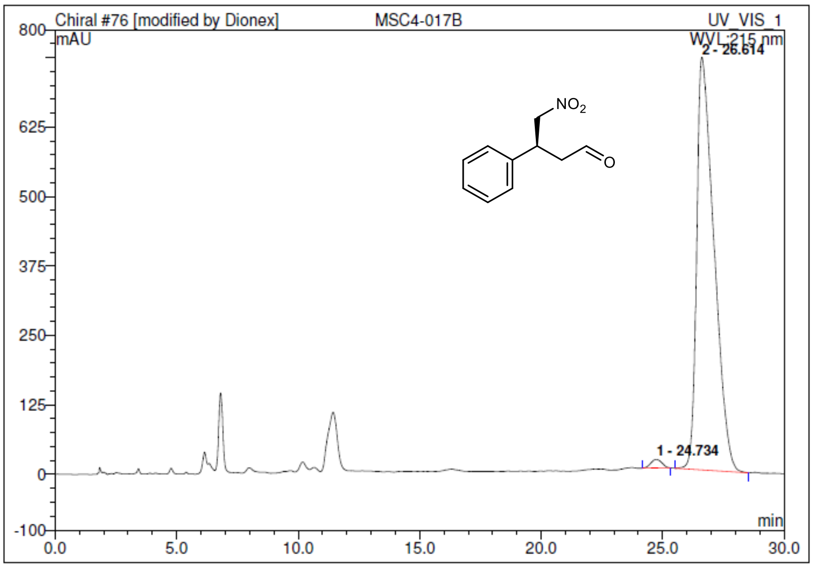

No.	Ret.Time min	Peak Name	Height mAU	Area mAU*min	Rel.Area %	Amount	Type
1	24.73	n.a.	15.242	8.042	1.29	n.a.	BMB*
2	26.61	n.a.	742.752	613.579	98.71	n.a.	BMB
Total:			757.995	621.621	100.00	0.000	

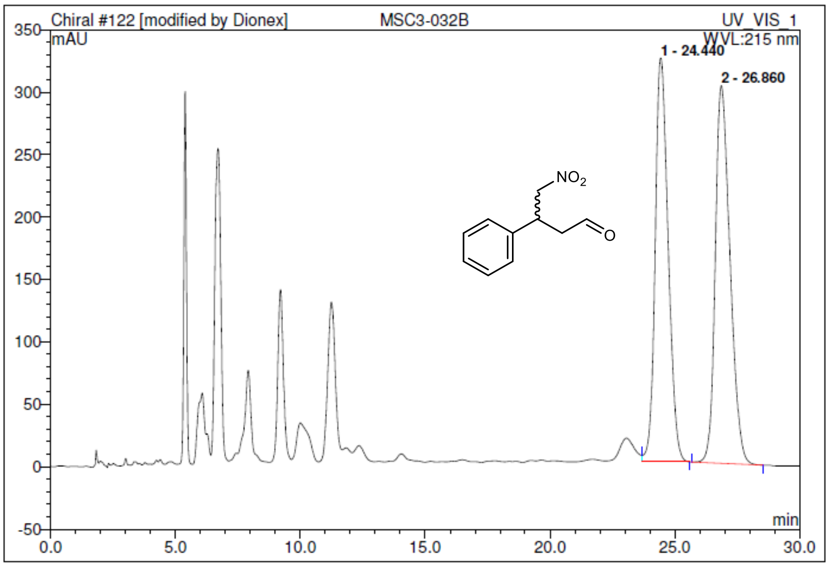

No.	Ret.Time min	Peak Name	Height mAU	Area mAU*min	Rel.Area %	Amount	Type
1	24.44	n.a.	323.540	194.775	48.46	n.a.	MB*
2	26.86	n.a.	302.747	207.116	51.54	n.a.	BMB
Total:			626.288	401.890	100.00	0.000	

Synthesis of racemate

A racemic sample was prepared by performing the above procedure on 5.0 mmol scale manually using ***rac*-Cat-2** as the catalyst. The product was obtained as a yellow oil (491 mg, 2.54 mmol, 51%).

7a ***(1'S,2'R,3'R)-2'-nitro-1',2',3',6'-tetrahydro-[1,1':3',1''-terphenyl]-4'-carbaldehyde***

7b ***(1'S,2'S,3'R)-2'-nitro-1',2',3',6'-tetrahydro-[1,1':3',1''-terphenyl]-4'-carbaldehyde***

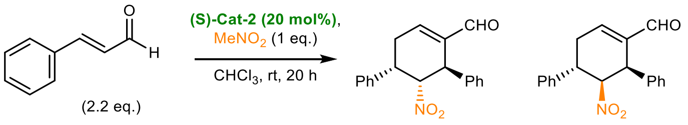

7a **7b**

Manual preparations

The system was configured as specified in the graph file `7.json` for the reaction.

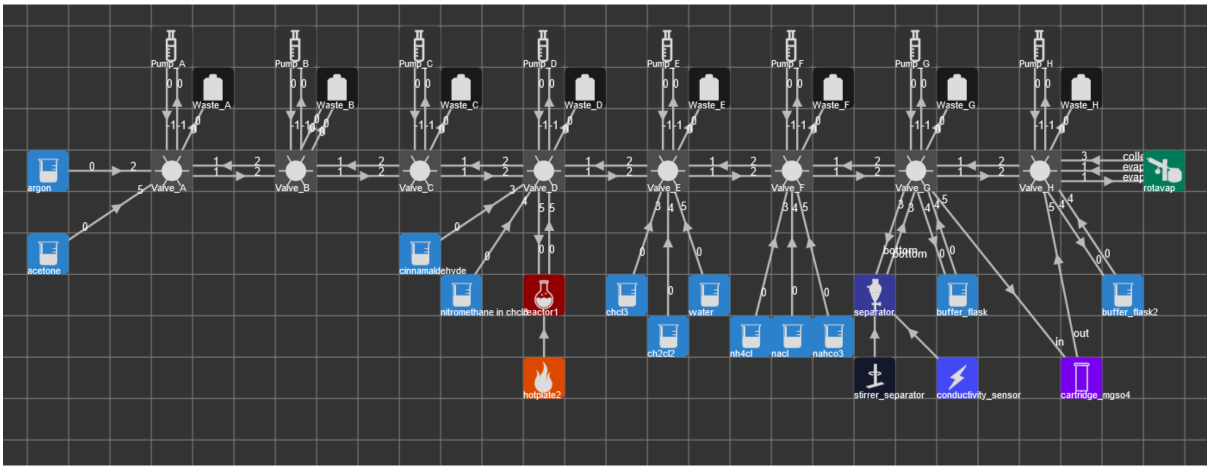

Figure S10: Graphical representation of `7.json`

Catalyst **(*S*)-Cat-2** was weighed and added into a reactor flask (reactor).

Automated synthesis

The following is an automated human-readable print out of the encoded steps. For full synthesis script refer to synthesis file `7.xdl`

```
Step 1: Add (S)-a,a-Bisphenyl-2-pyrrolidinemethanol (2 mmol) directly to reactor1 at default speed without stirring.
Step 2: Add cinnamaldehyde (22 mmol) directly to reactor1 at default speed with stirring at 250 RPM. Flush liquid handling apparatus with chcl3 (3 mL) after addition.
Step 3: Add nitromethane in chcl3 (10 mmol) directly to reactor1 at default speed with stirring at 250 RPM.
Step 4: Stir reactor1 for 20 h at 250 RPM stopping stirring afterwards.
Step 5: Add water (50 mL) directly to reactor1 at default speed with stirring at 250 RPM.
Step 6: Transfer all from reactor1 directly to separator at default speed, rinsing reactor1 with ch2cl2 (1 x 20 mL), flushing tubing after the transfer.
```

```
Step 7: Extract contents of separator with ch2cl2 (2 x 40 mL). Transfer waste
phase (top) to waste, and product phase (bottom) directly to separator.
Step 8: Transfer all from separator through mgso4 to rotavap at default
speed, rinsing separator with ch2cl2 (1 x 40 mL), without flushing tubing
after the transfer.
Step 9: Reset liquid handling apparatus with ch2cl2 (3 x 3 mL).
Step 10: Evaporate contents of rotavap with pressure 50 mbar at temperature
40 °C for 60 min.
Step 11: Evaporate contents of rotavap with pressure 1 mbar at temperature
40 °C for 15 min.
Step 12: Shut down the platform.
```

Purification

The crude product (*ca.* 1.4:1 dr) obtained from the automated synthesis was purified by column chromatography (25→50% Et_2O/PE) to afford the two major diastereoisomers of the title compound as off-white solids; **7a**: (1.51 g, 4.91 mmol, 49%, >99:1 er), **7b**: (1.10 g, 3.60 mmol, 36%, >99:1 er)

Analytical data

7a:

^{1}H NMR (600 MHz, $CDCl_3$) δ 9.60 (s, 1H), 7.42 – 7.35 (m, 3H), 7.35 – 7.18 (m, 6H), 7.08 – 7.03 (m, 2H), 4.97 (dd, *J* = 3.4, 1.8 Hz, 1H), 4.54 (s, 1H), 3.39 (ddd, *J* = 11.2, 6.0, 3.3 Hz, 1H), 3.28 (ddt, *J* = 20.1, 11.2, 2.4 Hz, 1H), 2.91 (dt, *J* = 20.1, 5.4 Hz, 1H).

^{13}C NMR (151 MHz, $CDCl_3$) δ 191.9, 150.5, 139.0, 138.3, 138.1, 129.4, 129.1, 128.2, 128.2, 127.5, 91.4, 43.3, 37.4, 28.1.

The analytical data is in accordance with that previously reported in the literature.(*16*)

Enantiomeric ratio was determined by chiral HPLC: Daicel Chiralpak IB N-5 column, hexane:isopropanol = 80:20, 1 mL/min flow rate, 215 nm detection wavelength. t_R (major) = 7.3 min, t_R (minor) = 14.6 min.

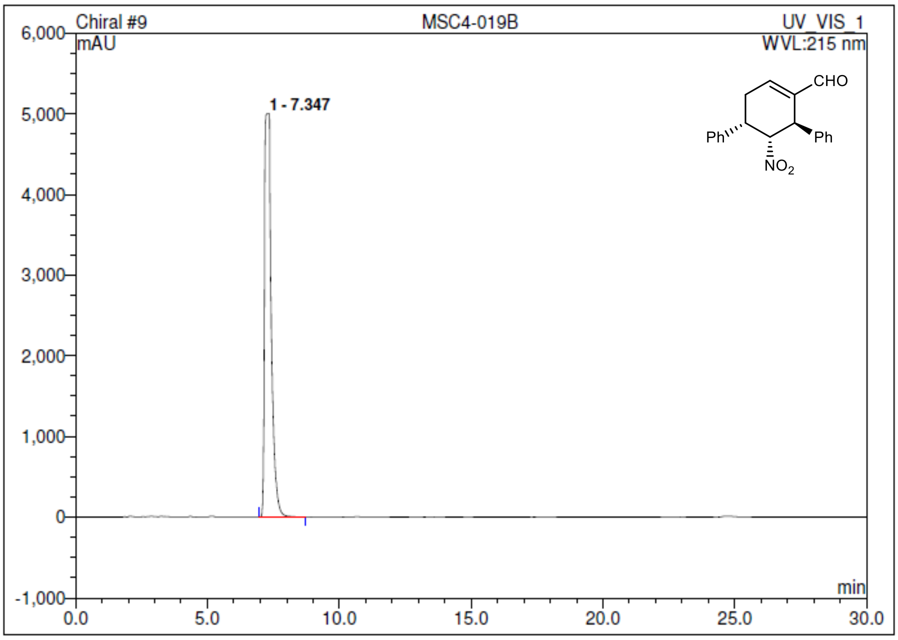

No.	Ret.Time min	Peak Name	Height mAU	Area mAU*min	Rel.Area %	Amount	Type
1	7.35	n.a.	4998.601	1481.784	100.00	n.a.	BMB
Total:			4998.601	1481.784	100.00	0.000	

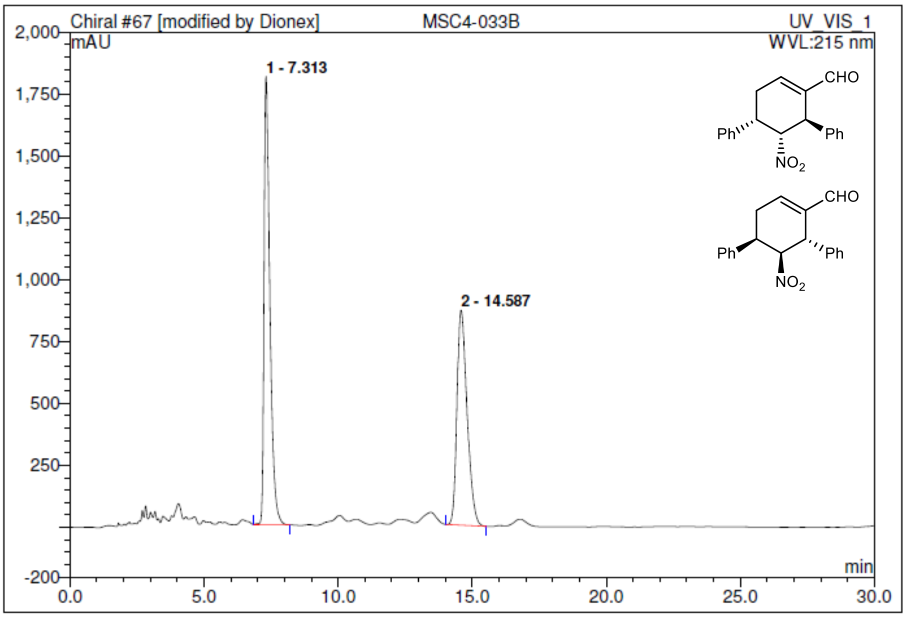

No.	Ret.Time min	Peak Name	Height mAU	Area mAU*min	Rel.Area %	Amount	Type
1	7.31	n.a.	1811.048	453.015	54.33	n.a.	BMB
2	14.59	n.a.	869.060	380.839	45.67	n.a.	BMB
Total:			2680.109	833.854	100.00	0.000	

7b:

^{1}H NMR (600 MHz, $CDCl_3$) δ 9.53 (s, 1H), 7.36 – 7.27 (m, 5H), 7.25 – 7.21 (m, 1H), 7.19 (dd, *J* = 7.1, 1.8 Hz, 2H), 7.11 (ddd, *J* = 9.7, 4.9, 2.5 Hz, 3H), 5.26 (dd, *J* = 12.4, 5.8 Hz, 1H), 4.71 (dd, *J* = 5.9, 1.7 Hz, 1H), 3.61 (ddd, *J* = 12.5, 10.7, 6.6 Hz, 1H), 3.12 (ddd, *J* = 21.0, 6.6, 4.6 Hz, 1H), 2.66 (ddt, *J* = 21.0, 10.7, 2.4 Hz, 1H).

^{13}C NMR (151 MHz, $CDCl_3$) δ 190.9, 148.1, 140.3, 140.2, 135.3, 129.2, 128.9, 128.8, 128.6, 127.9, 127.4, 89.4, 42.8, 38.0, 35.6.

The analytical data is in accordance with that previously reported in the literature.(*17*)

Enantiomeric ratio was determined by chiral HPLC: Daicel Chiralpak IC column, hexane:isopropanol = 80:20, 1 mL/min flow rate, 215 nm detection wavelength. t_R (major) = 27.5 min, t_R (minor) = 25.8 min.

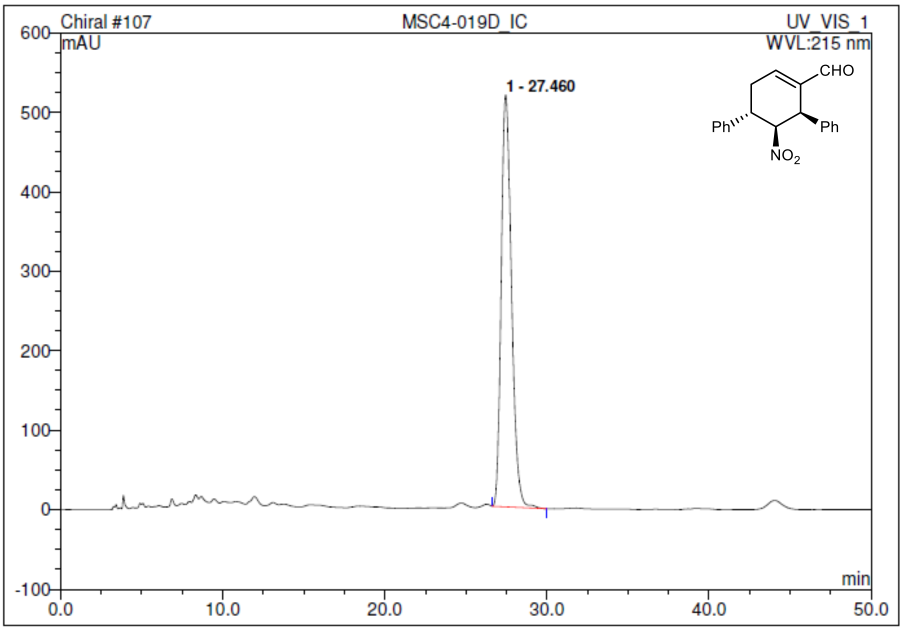

No.	Ret.Time min	Peak Name	Height mAU	Area mAU*min	Rel.Area %	Amount	Type
1	27.46	n.a.	518.402	381.395	100.00	n.a.	BMB
Total:			518.402	381.395	100.00	0.000	

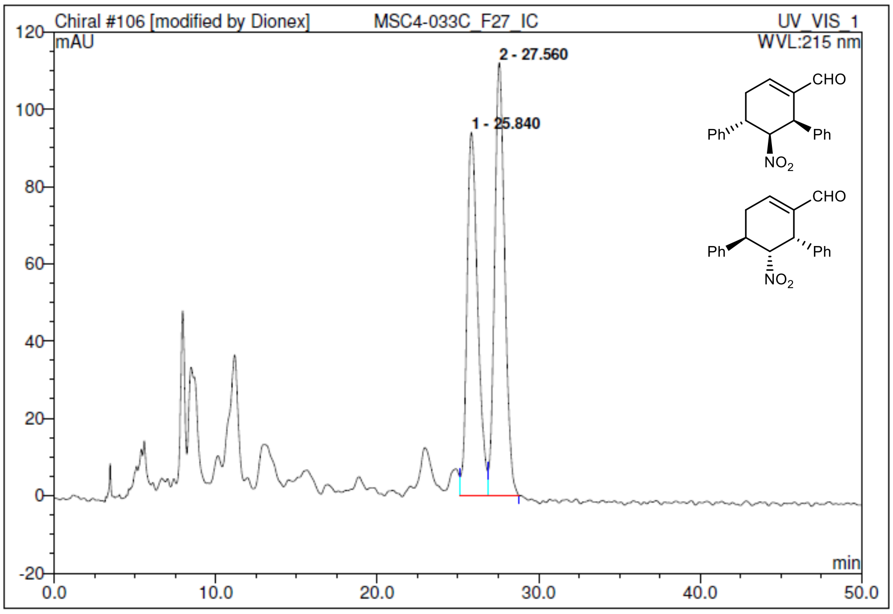

No.	Ret.Time min	Peak Name	Height mAU	Area mAU*min	Rel.Area %	Amount	Type
1	25.84	n.a.	93.994	72.630	47.03	n.a.	M *
2	27.56	n.a.	112.027	81.820	52.97	n.a.	MB*
Total:			206.020	154.450	100.00	0.000	

Synthesis of racemates

A racemic sample was prepared by performing the above procedure on 5.0 mmol scale manually using ***rac*-Cat-2** as the catalyst. The complex crude mixture, which contained the ***rac*-7a** and ***rac*-7b** as the major constituents, was purified by column chromatography to obtain analytical samples. ***Rac*-7b** could only be isolated as a major component in a complex mixture.

8 (R)-1-((1S,2S)-1-(benzylamino)-1,2-dihydronaphtho[2,1-b]furan-2-yl)pentan-1-ol

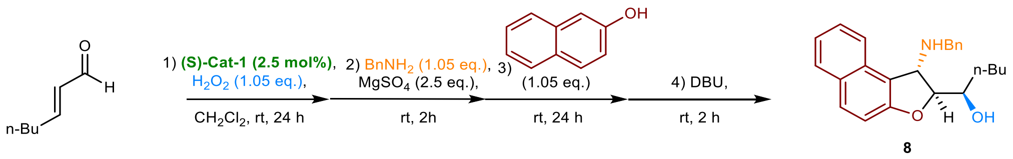

Manual preparations

The system was configured as specified in the graph file `8.json` for the reaction.

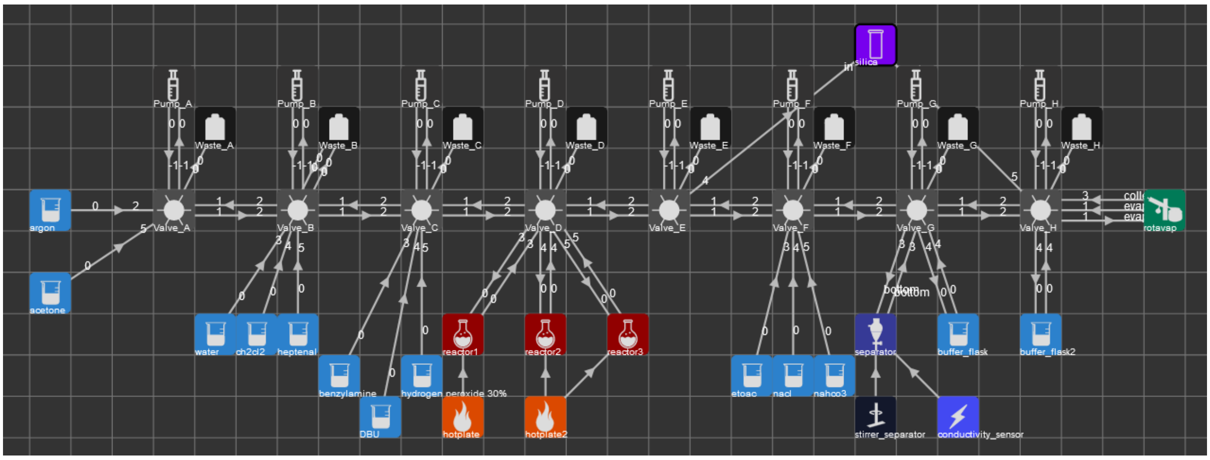

Figure S11: Graphical representation of `8.json`

Catalyst **(*S*)-Cat-1** was weighed and added into the corresponding reactor flask (reactor1).

Magnesium sulfate was weighed and added into the corresponding reactor flask (reactor2).

2-naphthol was weighed and added into the corresponding reactor flask (reactor3).

Automated synthesis

The following is an automated human-readable print out of the encoded steps. For full synthesis script refer to synthesis file `8.xdl`

```
Step 1: Reset liquid handling apparatus with ch2cl2 (3 x 3 mL).
Step 2: Add (S)-a,a-Bis[3,5-bis(trifluoromethyl)phenyl]-2-pyrrolidinemethanol (149.5 mg) directly to reactor1 at default speed without stirring.
Step 3: Add ch2cl2 (10 mL) directly to reactor1 at default speed with stirring at 250 RPM.
Step 4: Add heptenal (1.31 mL) directly to reactor1 at default speed with stirring at 250 RPM. Flush liquid handling apparatus with ch2cl2 (5 mL) after addition.
Step 5: Add hydrogen peroxide 30% (1.07 mL) directly to reactor1 at default speed with stirring at 250 RPM. Flush liquid handling apparatus with water (1 mL) after addition.
Step 6: Stir reactor1 for 24 h at 250 RPM stopping stirring afterwards.
```

```
Step 7: Add magnesium sulfate (3 g) directly to reactor2 at default speed without stirring.
Step 8: Transfer all from reactor1 directly to reactor2 at default speed, rinsing reactor1 with ch2cl2 (1 x 5 mL), without flushing tubing after the transfer.
Step 9: Add benzylamine (1.14 mL) directly to reactor2 at default speed with stirring at 250 RPM. Flush liquid handling apparatus with ch2cl2 (3 mL) after addition.
Step 10: Stir reactor2 for 2 h at 400 RPM stopping stirring afterwards.
Step 11: Add 2-naphthol (1.51 g) directly to reactor3 at default speed without stirring.
Step 12: Transfer all from reactor2 directly to reactor3 at default speed, rinsing reactor2 with ch2cl2 (1 x 10 mL), without flushing tubing after the transfer.
Step 13: Stir reactor3 for 24 h at 250 RPM stopping stirring afterwards.
Step 14: Add DBU (1.49 mL) directly to reactor3 at default speed with stirring at 250 RPM. Flush liquid handling apparatus with ch2cl2 (3 mL) after addition.
Step 15: Stir reactor3 for 2 h at 250 RPM stopping stirring afterwards.
Step 16: Transfer all from reactor3 through silica to rotavap at default speed, rinsing reactor3 with ch2cl2 (2 x 40 mL), without flushing tubing after the transfer.
Step 17: Evaporate contents of rotavap with pressure 50 mbar at temperature 40 °C for 60 min.
Step 18: Shut down the platform.
```

Purification

The crude product obtained from the automated synthesis was purified by column chromatography (5→15% EtOAc/PE) to afford the title compound as a viscous brown oil (2.10 g, 5.83 mmol, 58%, 98.5:1.5 *er*, >20:1 *dr*).

Analytical data

^{1}H NMR (600 MHz, $CDCl_3$) δ 7.87 (dd, *J* = 8.3, 1.1 Hz, 1H), 7.81 (d, *J* = 8.2 Hz, 1H), 7.74 (d, *J* = 8.8 Hz, 1H), 7.48 (ddd, *J* = 8.3, 6.8, 1.3 Hz, 1H), 7.38 – 7.35 (m, 2H), 7.35 – 7.29 (m, 3H), 7.24 (d, *J* = 7.3 Hz, 1H), 7.11 (d, *J* = 8.8 Hz, 1H), 5.01 (d, *J* = 3.5 Hz, 1H), 4.68 (dd, *J* = 5.4, 3.6 Hz, 1H), 3.87 (d, *J* = 13.1 Hz, 1H), 3.79 (d, *J* = 13.0 Hz, 1H), 3.77 (td, *J* = 5.5, 2.8 Hz, 1H), 1.75 (s, 1H), 1.73 – 1.67 (m, 1H), 1.61 – 1.51 (m, 2H), 1.45 – 1.31 (m, 3H), 0.92 (t, *J* = 7.1 Hz, 3H).

^{13}C NMR (151 MHz, $CDCl_3$) δ 157.9, 140.5, 131.2, 131.0, 129.7, 129.0, 128.6, 128.5, 127.3, 127.2, 123.3, 122.5, 119.0, 112.3, 91.7, 72.7, 60.8, 50.1, 32.3, 27.9, 22.8, 14.2.

HRMS (ESI) calculated for $C_{24}H_{28}NO_2$ $(M+H)^+$: 362.2115, found: 362.2083.

Absolute and relative chemistry has been assigned in analogy to the literature reported examples.(*18*)

Enantiomeric ratio was determined by chiral HPLC: Daicel Chiralpak IB N-5 column, hexane:isopropanol = 99:01, 1 mL/min flow rate, 215 nm detection wavelength. t_R (major) = 13.5 min, t_R (minor) = 12.8 min.

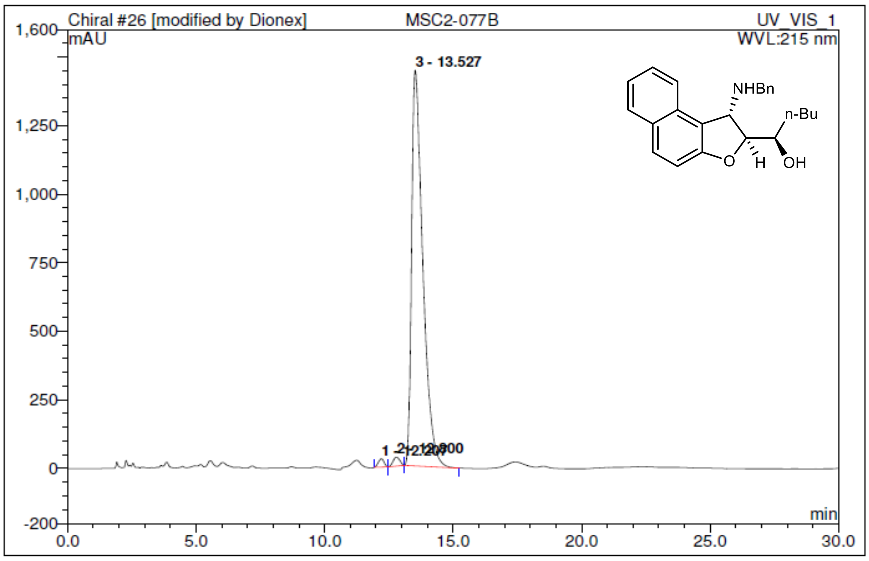

No.	Ret.Time min	Peak Name	Height mAU	Area mAU*min	Rel.Area %	Amount	Type
1	12.21	n.a.	30.096	7.637	1.02	n.a.	BMb*
2	12.80	n.a.	32.543	9.839	1.32	n.a.	bMB*
3	13.53	n.a.	1443.441	730.566	97.66	n.a.	BMB
Total:			1506.079	748.042	100.00	0.000	

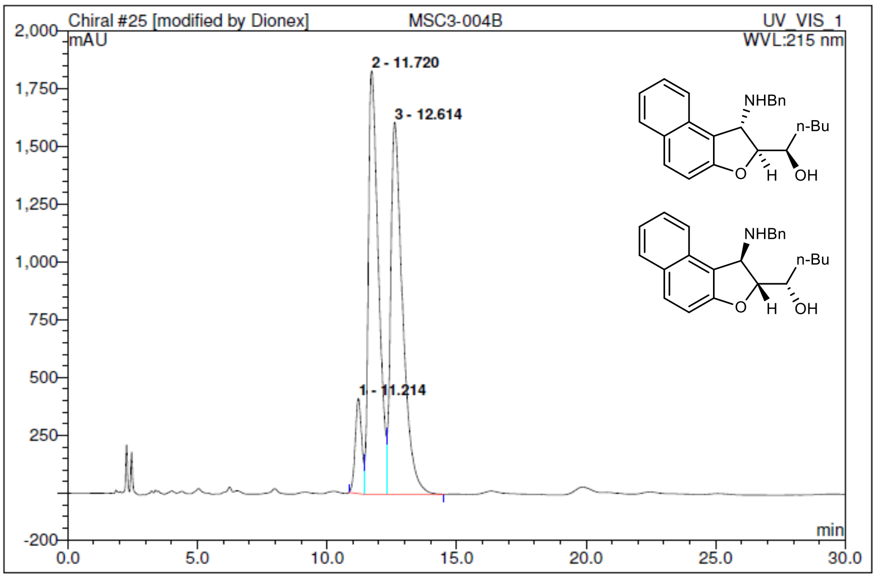

No.	Ret.Time min	Peak Name	Height mAU	Area mAU*min	Rel.Area %	Amount	Type
1	11.21	n.a.	411.164	119.204	6.62	n.a.	BM *
2	11.72	n.a.	1830.646	816.145	45.34	n.a.	M *
3	12.61	n.a.	1608.333	864.905	48.04	n.a.	MB*
Total:			3850.143	1800.254	100.00	0.000	

Synthesis of racemate

A racemic sample was prepared by performing the above procedure on 5.0 mmol scale manually using ***rac*-Cat-1** as the catalyst. The product was obtained as a brown oil (500 mg, 1.39 mmol, 29%).

9a (R)-2-((S)-2-nitro-1-phenylethyl)pentanal

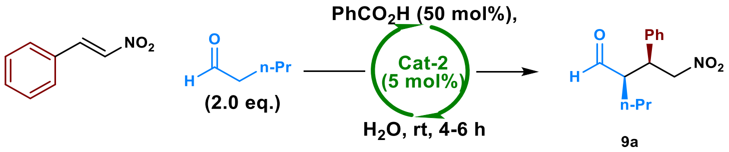

Modifications from literature conditions

Initially, the protocol described below was performed with previously reported literature conditions (5 h reaction time for each cycle). Unfortunately, in our hands this led to poorer diastereoselectivity in the initial cycles, and poorer conversion/yield in the later cycles. For example, below are the results of two separate automated runs using 5 h reaction times for each cycle:

Table S2: Catalyst recycling with 5 h reaction time for each cycle. Conversions and diastereomeric ratios were determined by ^{1}H NMR analysis of the crude products obtained from automated synthesis runs. Conversion was measured as a ratio between the product and the nitrostyrene starting material.

	Run 1	Run 2
**Cycle 1**	>99% conv, 91:9 dr	>99% conv, 91:9 dr
**Cycle 2**	>99% conv, 94:6 dr	97% conv, 93:7 dr
**Cycle 3**	95% conv, 95:5 dr	92% conv, 95:5 dr
**Cycle 4**	86% conv, 95:5 dr	76% conv, 96:4 dr

We reasoned that the inverse relationship between conversion and diastereoselectivity must indicate that once the reaction has reached high conversion, a reversible iminium-enamine-iminum formation of between the catalyst and the product scrambles the α-stereo center. Additionally, cumulative catalyst decomposition decreases the conversions observed in the later cycles. Therefore, we attempted to shorten the reaction time for the first cycle (from 5 hours to 4 hours) and prolong the fourth cycle (from 5 hours to 6 hours). The reaction times were conveniently encoded through the use of *Parameters* in the main XDL section. These conditions were not optimised further as they provided satisfactory results.

Manual preparations

The system was configured as specified in the graph file `9a.json` for the reaction.

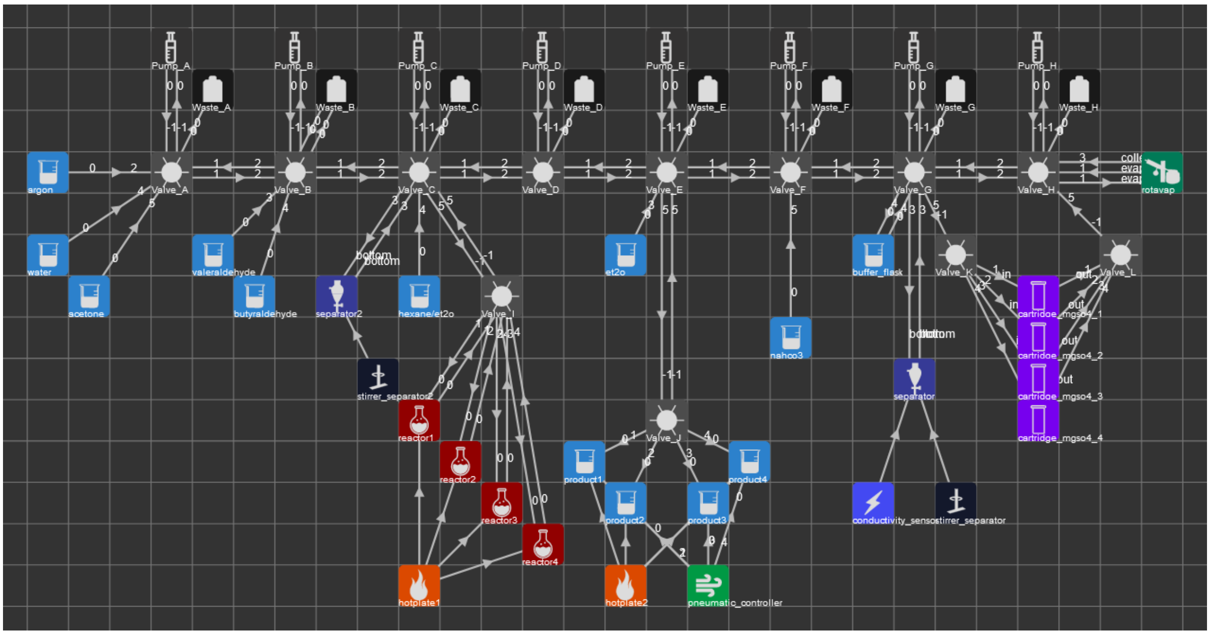

Figure S12: Graphical representation of `9a.json`

Catalyst **(*S*)-Cat-3** was weighed and added into the corresponding reactor flask (reactor1).

4 portions of trans-*β*-nitrostyrene and benzoic acid were weighed and added into the corresponding reactor flasks (reactor1, reactor2, reactor3, reactor4).

Automated synthesis

The synthesis was encoded with the help of four blueprints, each corresponding to a different stage of the reaction: reaction setup, catalyst recycling, product isolation, and glassware cleaning. The following is an automated human-readable print out of the encoded steps for the synthesis.

Note: The step list below includes the parameters and properties passed into the blueprints for this synthesis and is not representative of a general Blueprint.

Note: Execution of some steps belonging to different blueprints/iterations is parallelized. For illustration and explanation see main-text Figure 6 and the accompanying discussion.

For general blueprints and full synthesis script refer to synthesis file **`9a.xdl`**.

```
Step 1: Add catalyst (0.5 mmol) directly to reactor1 at default speed without
stirring.
Step 2: Add water (15 mL) directly to separator2 at default speed without
stirring.
```

```
Step 3: Repeat whilst iterating over type = reactor, type = cartridge, type = flask, type = time:
    RAMA_reaction
    RAMA_recycling
    RAMA_workup
    Cleaning
Step 4: Shut down the platform.
```

The following is an automated human-readable print out of the encoded steps for each of the blueprints. For brevity, only the first cycle is shown. The following cycles are identical except for the reaction times and the vessels used.

Blueprint *RAMA_reaction:*

```
Step 1: Add trans-nitrostyrene (1 eq.) directly to reactor1 at default speed without stirring.
Step 2: Add benzoic acid (0.5 eq.) directly to reactor1 at default speed without stirring.
Step 3: Transfer all from separator2 directly to reactor1 at default speed, flushing tubing after the transfer.
Step 4: Add valeraldehyde (2 eq.) directly to reactor1 at default speed with stirring at 250 RPM. Flush liquid handling apparatus with water (1 mL) after addition.
Step 5: Stir reactor1 for 4 h at 250 RPM stopping stirring afterwards.
```

Blueprint *RAMA_recycling:*

```
Step 1: Repeat 2 times:
    Transfer all from reactor1 directly to separator2 at default speed, rinsing reactor1 with hexane/et2o (3 x 15 mL), flushing tubing after the transfer.
    Stir separator2 for 2 min at 600 RPM stopping stirring afterwards.
    Wait for 2 min.
    Transfer 2.5 mL from separator2 directly to reactor1 at default speed, without flushing tubing after the transfer.
    Transfer 15 mL from separator2 directly to reactor1 at default speed, flushing tubing after the transfer.
    Transfer all from separator2 directly to separator at default speed, flushing tubing after the transfer.

Step 2: Transfer all from reactor1 directly to separator2 at default speed, flushing tubing after the transfer.
```

Blueprint *RAMA_workup:*

```
Step 1: Wait for 10 min.
Step 2: Wash contents of separator with nahco3 (2 x 50 mL). Transfer waste phase (bottom) to waste, and product phase (top) directly to separator.
Step 3: Transfer all from separator directly to rotavap at default speed, rinsing separator with et2o (1 x 30 mL), flushing tubing after the transfer.
Step 4: Reset liquid handling apparatus with et2o (3 x 3 mL).
Step 5: Evaporate contents of rotavap with default pressure control at temperature 40 °C for 30 min.
Step 6: Evaporate contents of rotavap with pressure 1 mbar at temperature 40 °C for 10 min.
Step 7: Repeat 2 times:
```

```
    Add et2o (7 mL) directly to rotavap at default speed with stirring at
250 RPM.
    Transfer 15 mL from rotavap directly to product1 at default speed,
flushing tubing after the transfer.
Step 8: Reset liquid handling apparatus with et2o (3 x 10 mL).
Step 9: Heat/Chill product1 to 35 °C without stirring. Temperature control
is continued after the temperature has been reached.
Step 10: Purge product1 with inert gas for 30 min.
Step 11: Stop heating/chilling product1.
```

Blueprint *Cleaning:*

```
Step 1: Clean separator2 with acetone (1 × 30 mL) without temperature control,
with drying if possible, stirring for 60 s at 500 RPM.
Step 2: Clean separator2 with water (1 × 30 mL) without temperature control,
with drying if possible, stirring for 60 s at 500 RPM.
Step 3: Clean separator with acetone (1 × 30 mL) without temperature control,
with drying if possible, stirring for 60 s at 500 RPM.
Step 4: Clean separator with water (1 × 30 mL) without temperature control,
with drying if possible, stirring for 60 s at 500 RPM.
Step 5: Reset liquid handling apparatus with acetone (3 x 10 mL).
Step 6: Reset liquid handling apparatus with et2o (3 x 10 mL).
Step 7: Clean rotavap with et2o (1 × 30 mL) without temperature control,
without drying, stirring for 60 s at 150 RPM.
```

Purification

Each of the products collected in product flasks (product1, product2, product3, product4) was purified by column chromatography (0→20% EtOAc/PE) to afford four batches of the title compound.

Batch 1: pale yellow oil (2.28 g, 9.68 mmol, 97%, *dr* 93.5:6.5, *er* 99.5:0.5)

Batch 2: pale yellow oil (2.28 g, 9.68 mmol, 97%, *dr* 95:5, *er* 99.5:0.5)

Batch 3: pale yellow oil (2.13 g, 9.04 mmol, 90%, *dr* 95:5, *er* 99.5:0.5)

Batch 4: pale yellow oil (2.08 g, 8.84 mmol, 88%, *dr* 96:4, *er* 99.5:0.5)

Analytical data

^{1}H NMR (600 MHz, $CDCl_3$) δ 9.71 (d, *J* = 2.8 Hz, 1H), 7.37 – 7.33 (m, 2H), 7.32 – 7.28 (m, 1H), 7.20 – 7.16 (m, 2H), 4.70 (dd, *J* = 12.8, 5.2 Hz, 1H), 4.65 (dd, *J* = 12.8, 9.6 Hz, 1H), 3.78 (td, *J* = 9.6, 5.2 Hz, 1H), 2.70 (tt, *J* = 9.4, 3.2 Hz, 1H), 1.53 – 1.44 (m, 1H), 1.41 – 1.28 (m, 2H), 1.23 – 1.13 (m, 1H), 0.80 (t, *J* = 7.1 Hz, 3H).

^{13}C NMR (151 MHz, $CDCl_3$) δ 203.4, 137.0, 129.3, 128.3, 128.2, 78.6, 54.0, 43.4, 29.7, 20.0, 14.1.

The analytical data is in accordance with that previously reported in the literature.(*10*)

Enantiomeric ratio was determined by chiral HPLC: Daicel Chiralpak IB N-5 column, hexane:isopropanol = 80:20, 1 mL/min flow rate, 215 nm detection wavelength. t_R (major, syn) = 9.8 min, t_R (minor, syn) = 7.7 min, t_R (anti) = 8.5 min, 13.5 min.

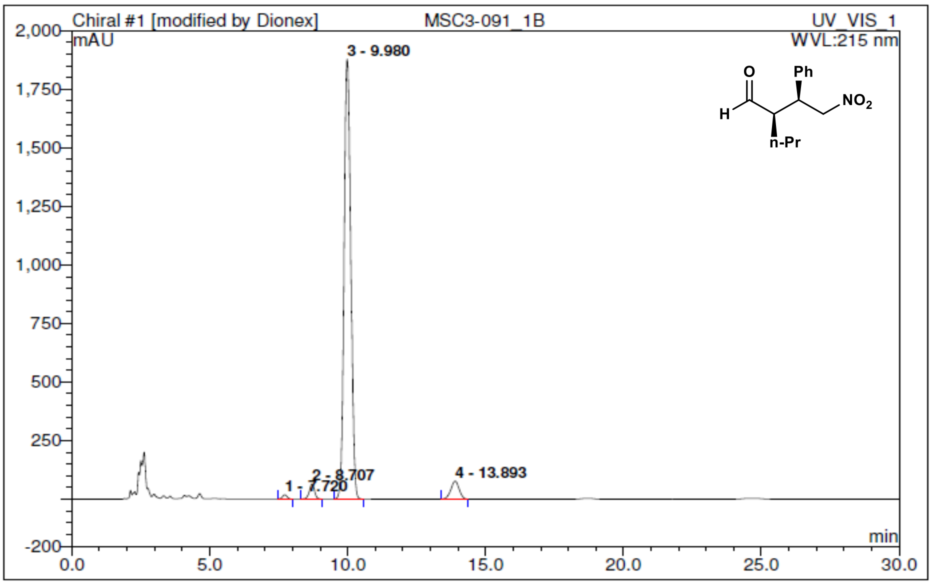

No.	Ret.Time min	Peak Name	Height mAU	Area mAU*min	Rel.Area %	Amount	Type
1	7.72	n.a.	17.636	3.277	0.57	n.a.	BMB*
2	8.71	n.a.	63.915	13.976	2.42	n.a.	BMB*
3	9.98	n.a.	1877.975	532.443	92.38	n.a.	BMB*
4	13.89	n.a.	76.594	26.641	4.62	n.a.	BMB*
Total:			2036.120	576.338	100.00	0.000	

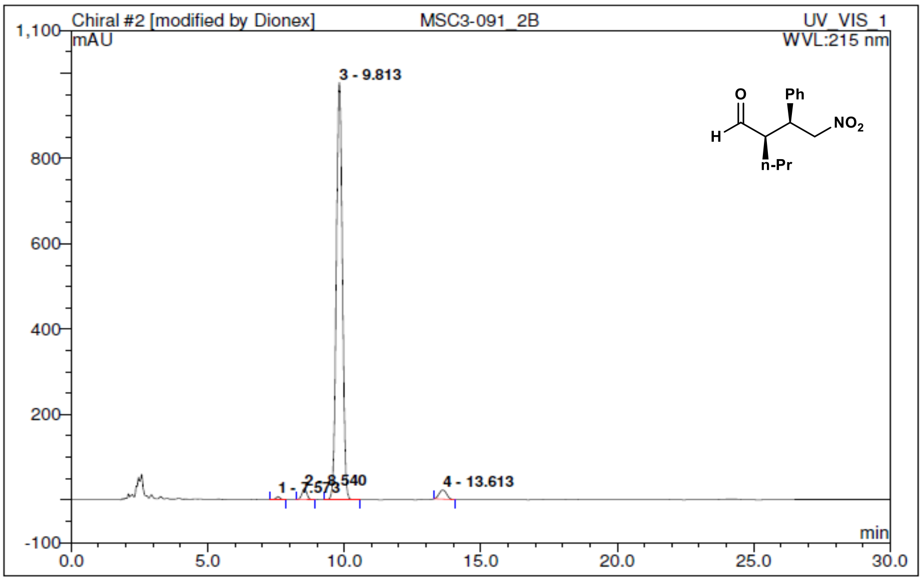

No.	Ret.Time min	Peak Name	Height mAU	Area mAU*min	Rel.Area %	Amount	Type
1	7.57	n.a.	6.539	1.192	0.45	n.a.	BMB*
2	8.54	n.a.	25.350	5.277	1.99	n.a.	BMB*
3	9.81	n.a.	977.879	251.386	95.00	n.a.	BMB
4	13.61	n.a.	21.347	6.748	2.55	n.a.	BMB*
Total:			1031.115	264.603	100.00	0.000	

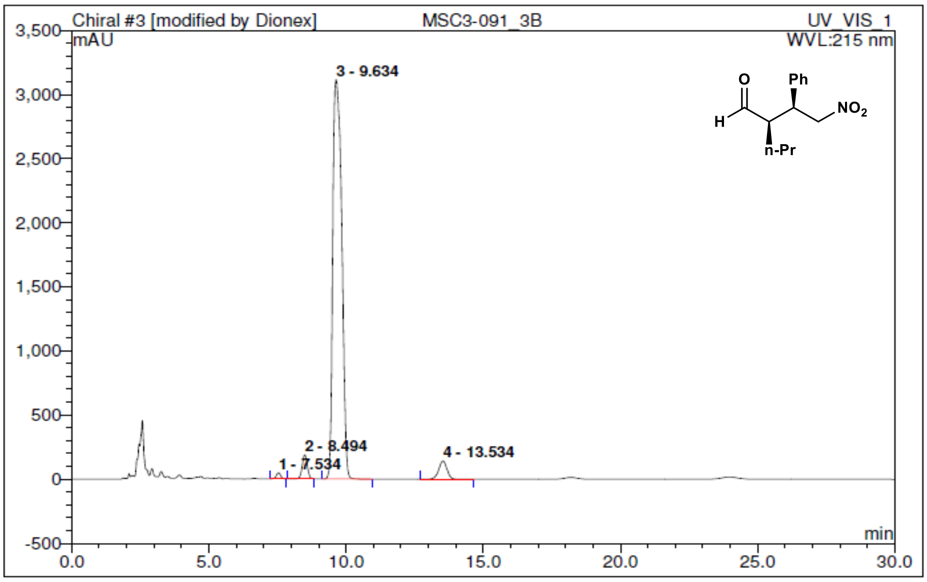

No.	Ret.Time min	Peak Name	Height mAU	Area mAU*min	Rel.Area %	Amount	Type
1	7.53	n.a.	47.343	8.579	0.67	n.a.	BMB*
2	8.49	n.a.	187.627	40.053	3.15	n.a.	BMB
3	9.63	n.a.	3116.474	1175.833	92.36	n.a.	BMB
4	13.53	n.a.	141.586	48.697	3.82	n.a.	BMB
Total:			3493.029	1273.163	100.00	0.000	

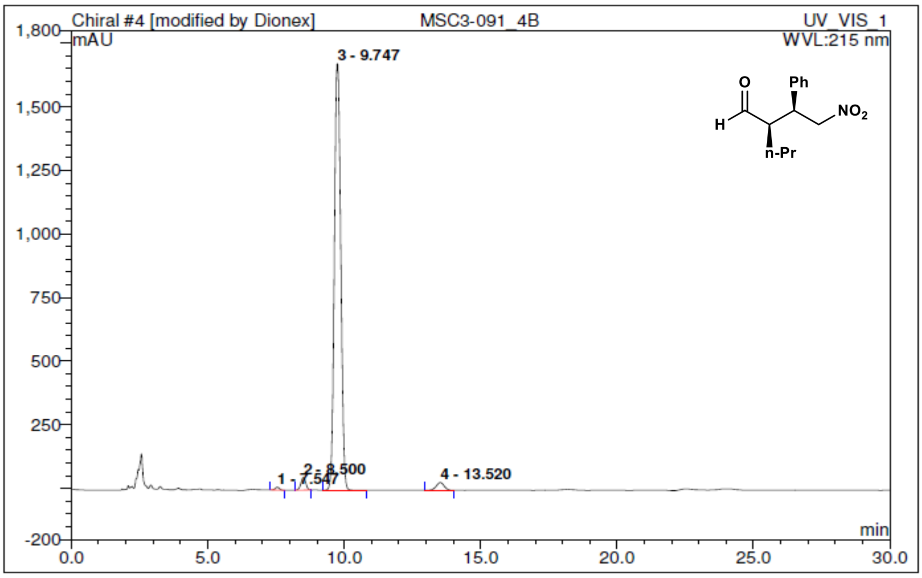

No.	Ret.Time min	Peak Name	Height mAU	Area mAU*min	Rel.Area %	Amount	Type
1	7.55	n.a.	11.249	2.011	0.43	n.a.	BMB*
2	8.50	n.a.	48.990	10.084	2.13	n.a.	BMB*
3	9.75	n.a.	1676.773	450.551	95.30	n.a.	BMB
4	13.52	n.a.	30.171	10.136	2.14	n.a.	BMB*
Total:			1767.183	472.782	100.00	0.000	

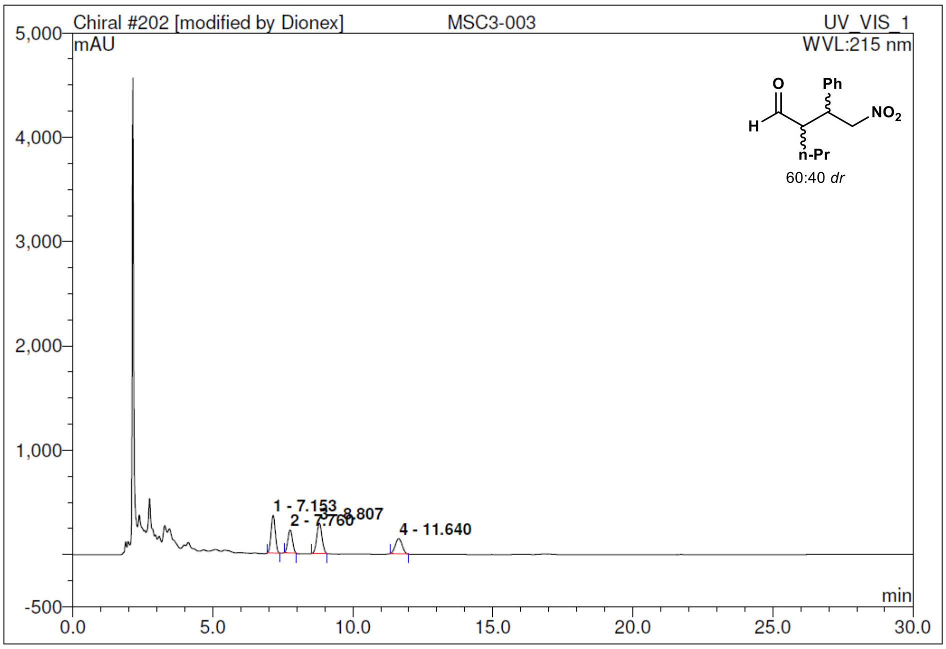

No.	Ret.Time min	Peak Name	Height mAU	Area mAU*min	Rel.Area %	Amount	Type
1	7.15	n.a.	360.112	63.389	30.37	n.a.	BMB*
2	7.76	n.a.	218.929	41.016	19.65	n.a.	BMB*
3	8.81	n.a.	291.155	63.317	30.33	n.a.	BMB*
4	11.64	n.a.	145.455	41.023	19.65	n.a.	BMB*
Total:			1015.652	208.745	100.00	0.000	

Synthesis of racemate

A racemic sample was prepared as follows:

To a solution of trans-β-nitrostyrene (745 mg, 5.0 mmol) in CH_2Cl_2 (5.0 mL) was added valeraldehyde (1.06 mL, 10.0 mmol, 2.0 eq.), and pyrrolidine (82 µL, 1 mmol, 0.2 eq.), and the mixture was stirred at room temperature for 16 hours. The solvent was removed *in vacuo* and the residue was purified by column chromatography (10→20% EtOAc/PE) to afford ***rac*-9a** as a mixture of diastereoisomers (60:40 *dr*). The sample also contained a considerable amount of homoaldol condensation product which co-eluted with the product.

9a (R)-2-((S)-2-nitro-1-phenylethyl)pentanal

9b (2R,3S)-2-ethyl-4-nitro-3-phenylbutanal

9c (2R,3R)-2-ethyl-3-(furan-2-yl)-4-nitrobutanal

9d (R)-2-((R)-1-(furan-2-yl)-2-nitroethyl)pentanal

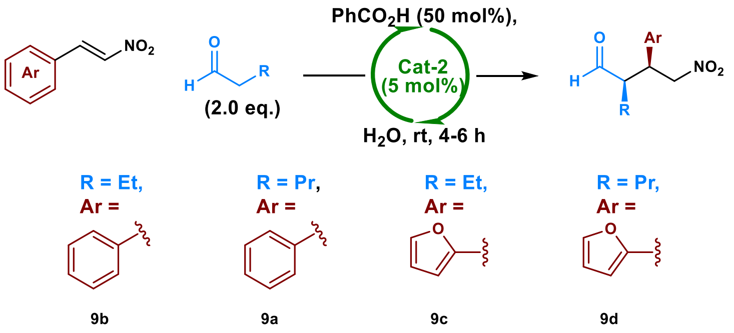

Manual preparations

The system was configured as specified in the graph file `9a-d.json` for the reaction.

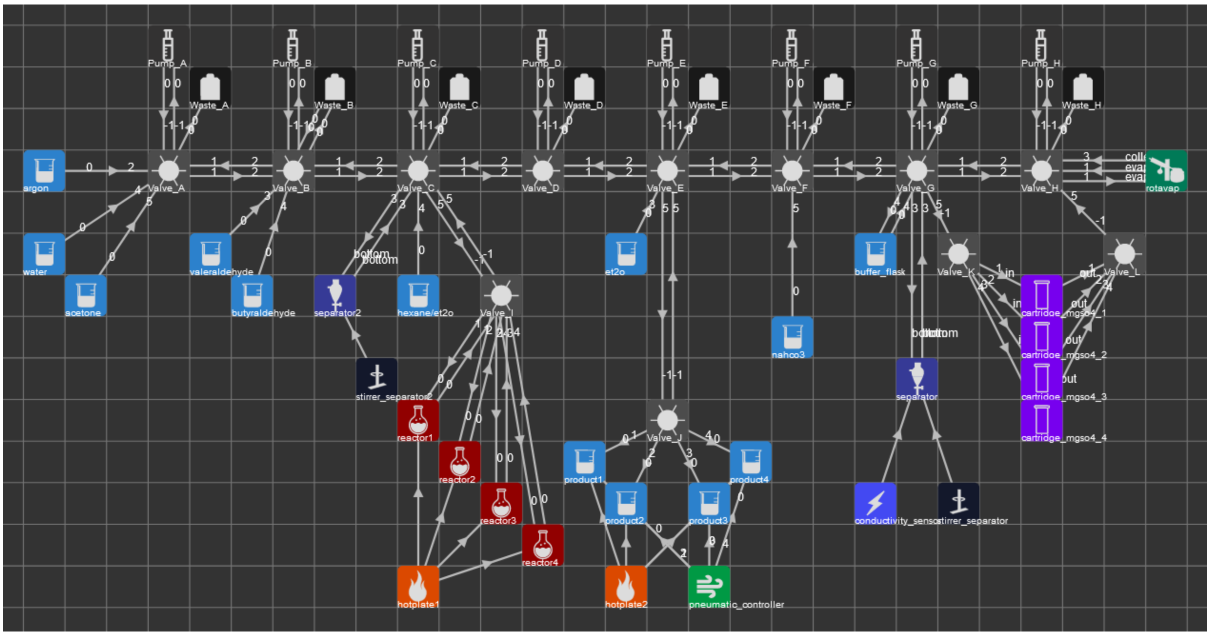

Figure S13: Graphical representation of `9a-d.json`

Catalyst **(*S*)-Cat-3** was weighed and added into the corresponding reactor flask (reactor1).

2 portions of trans-*β*-nitrostyrene were weighed and added to the corresponding reactor flasks (reactor1, reactor2).

2 portions of 2-(nitrovinyl)-furan were weighed and added to the corresponding reactor flasks (reactor3, reactor4).

4 portions of benzoic acid were weighed and added into the corresponding reactor flasks (reactor1, reactor2, reactor3, reactor4).

Automated synthesis

The synthesis was encoded with the help of four blueprints, each corresponding to a different stage of the reaction: reaction setup, catalyst recycling, product isolation, and glassware cleaning. The following is an automated human-readable print out of the encoded steps for the synthesis.

Note: The step list below includes the parameters and properties passed into the blueprints for this synthesis and is not representative of a general Blueprint.

Note: Execution of some steps belonging to different blueprints/iterations is parallelized. For illustration and explanation see main-text Figure 6 and the accompanying discussion.

For general blueprints and full synthesis script refer to synthesis file **`9a-d.xdl`**.

```
Step 1: Add catalyst (0.5 mmol) directly to reactor1 at default speed without
stirring.
Step 2: Add water (15 mL) directly to separator2 at default speed without
stirring.
Step 3:
 Repeat whilst iterating over role = nitroolefin:
  Repeat whilst iterating over role = aldehyde:
    Repeat whilst iterating over type = reactor, type = cartridge, type =
flask, type = time:
    RAMA_reaction
    RAMA_recycling
    RAMA_workup
    Cleaning
Step 4: Shut down the platform.
```

The following is an automated human-readable print out of the encoded steps for each of the blueprints. For brevity, only the first cycle is shown. The following cycles are identical except for the reaction times, the starting materials (aldehyde and nitroolefin), and the vessels (e.g. *reactor1→reactor2*, *product1→product2*) used.

Blueprint *RAMA_reaction:*

```
Step 1: Add trans-nitrostyrene (1 eq.) directly to reactor1 at default speed
without stirring.
Step 2: Add benzoic acid (0.5 eq.) directly to reactor1 at default speed
without stirring.
Step 3: Transfer all from separator2 directly to reactor1 at default speed,
flushing tubing after the transfer.
Step 4: Add butyraldehyde (2 eq.) directly to reactor1 at default speed with
stirring at 250 RPM. Flush liquid handling apparatus with water (1 mL) after
addition.
Step 5: Stir reactor1 for 4 h at 250 RPM stopping stirring afterwards.
```

Blueprint *RAMA_recycling:*

Step 1: Repeat 2 times:
    Transfer all from reactor1 directly to separator2 at default speed, rinsing reactor1 with hexane/et2o (3 x 15 mL), flushing tubing after the transfer.
    Stir separator2 for 2 min at 600 RPM stopping stirring afterwards.
    Wait for 2 min.
    Transfer 2.5 mL from separator2 directly to reactor1 at default speed, without flushing tubing after the transfer.
    Transfer 15 mL from separator2 directly to reactor1 at default speed, flushing tubing after the transfer.
    Transfer all from separator2 directly to separator at default speed, flushing tubing after the transfer.
Step 2: Transfer all from reactor1 directly to separator2 at default speed, flushing tubing after the transfer.

Blueprint *RAMA_workup:*

Step 1: Wait for 10 min.
Step 2: Wash contents of separator with nahco3 (2 x 50 mL). Transfer waste phase (bottom) to waste, and product phase (top) directly to separator.
Step 3: Transfer all from separator directly to rotavap at default speed, rinsing separator with et2o (1 x 30 mL), flushing tubing after the transfer.
Step 4: Reset liquid handling apparatus with et2o (3 x 3 mL).
Step 5: Evaporate contents of rotavap with default pressure control at temperature 40 °C for 30 min.
Step 6: Evaporate contents of rotavap with pressure 1 mbar at temperature 40 °C for 10 min.
Step 7: Repeat 2 times:
    Add et2o (7 mL) directly to rotavap at default speed with stirring at 250 RPM.
    Transfer 15 mL from rotavap directly to product1 at default speed, flushing tubing after the transfer.
Step 8: Reset liquid handling apparatus with et2o (3 x 10 mL).
Step 9: Heat/Chill product1 to 35 °C without stirring. Temperature control is continued after the temperature has been reached.
Step 10: Purge product1 with inert gas for 30 min.
Step 11: Stop heating/chilling product1.

Blueprint *Cleaning:*

Step 1: Clean separator2 with acetone (1 × 30 mL) without temperature control, with drying if possible, stirring for 60 s at 500 RPM.
Step 2: Clean separator2 with water (1 × 30 mL) without temperature control, with drying if possible, stirring for 60 s at 500 RPM.
Step 3: Clean separator with acetone (1 × 30 mL) without temperature control, with drying if possible, stirring for 60 s at 500 RPM.
Step 4: Clean separator with water (1 × 30 mL) without temperature control, with drying if possible, stirring for 60 s at 500 RPM.
Step 5: Reset liquid handling apparatus with acetone (3 x 10 mL).
Step 6: Reset liquid handling apparatus with et2o (3 x 10 mL).
Step 7: Clean rotavap with et2o (1 × 30 mL) without temperature control, without drying, stirring for 60 s at 150 RPM.

Purification

Each of the products collected in product flasks (product1, product2, product3, product4) was purified by column chromatography (0→20% EtOAc/PE) to afford products **9a-d**

9b (Batch 1): pale-yellow oil, which solidified in a -20 °C freezer (2.05 g, 9.25 mmol, 93%, *dr* 94:6, *er* 99:5:0.5)

9a (Batch 2): pale-yellow oil (2.23 g, 9.50 mmol, 95%, *dr* 94:6, *er* 99.5:0.5)

9c (Batch 3): yellow oil, which solidified in a -20 °C freezer (1.96 g, 9.27 mmol, 93%, *dr* 92:8, *er* 99:1)

9d (Batch 4): yellow oil (2.03 g, 9.04 mmol, 90%, *dr* 94:6, *er* 98.5:1.5)

Analytical data

9b:

^{1}H NMR (600 MHz, $CDCl_3$) δ 9.72 (d, *J* = 2.6 Hz, 1H), 7.37 – 7.32 (m, 2H), 7.32 – 7.28 (m, 1H), 7.20 – 7.16 (m, 2H), 4.72 (dd, *J* = 12.7, 4.9 Hz, 1H), 4.63 (dd, *J* = 12.7, 9.7 Hz, 1H), 3.79 (td, *J* = 9.8, 4.9 Hz, 1H), 2.68 (dddd, *J* = 10.2, 7.6, 4.8, 2.6 Hz, 1H), 1.58 – 1.47 (m, 2H), 0.84 (t, *J* = 7.5 Hz, 3H).

^{13}C NMR (151 MHz, $CDCl_3$) δ 203.3, 137.0, 129.3, 129.3, 128.3, 128.2, 78.7, 55.2, 42.9, 20.6, 10.9.

Enantiomeric ratio was determined by chiral HPLC: Daicel Chiralpak IB N-5 column, hexane:isopropanol = 80:20, 1 mL/min flow rate, 215 nm detection wavelength. t_R (major, syn) = 10.0 min, t_R (minor, syn) = 8.3 min, t_R (anti) = 8.8 min, 14.8 min.

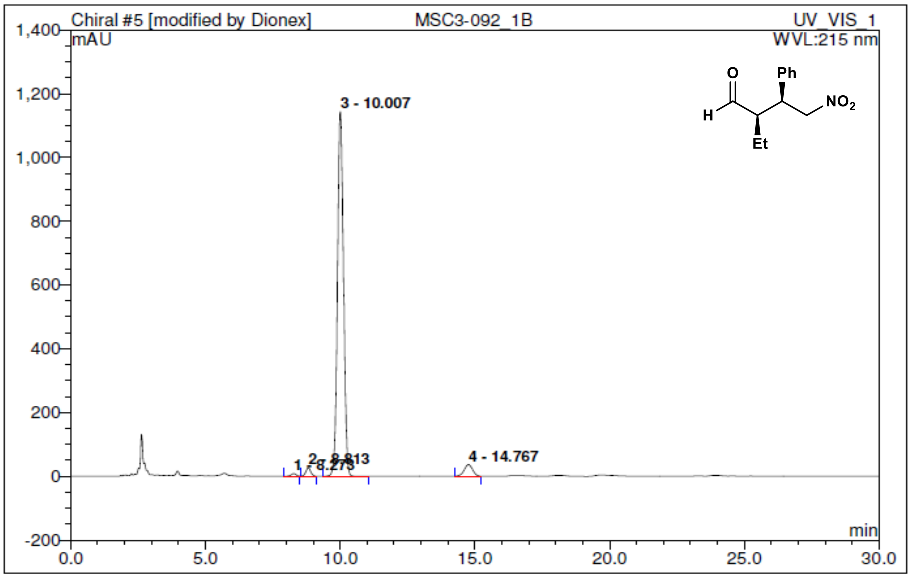

No.	Ret.Time min		Peak Name	Height mAU	Area mAU*min	Rel.Area %	Amount	Type
1	8.27	n.a.		7.676	1.481	0.47	n.a.	BMB*
2	8.81	n.a.		25.742	5.398	1.70	n.a.	BMB*
3	10.01	n.a.		1143.698	298.373	93.76	n.a.	BMB
4	14.77	n.a.		36.235	12.990	4.08	n.a.	BMB*
Total:				1213.351	318.243	100.00	0.000	

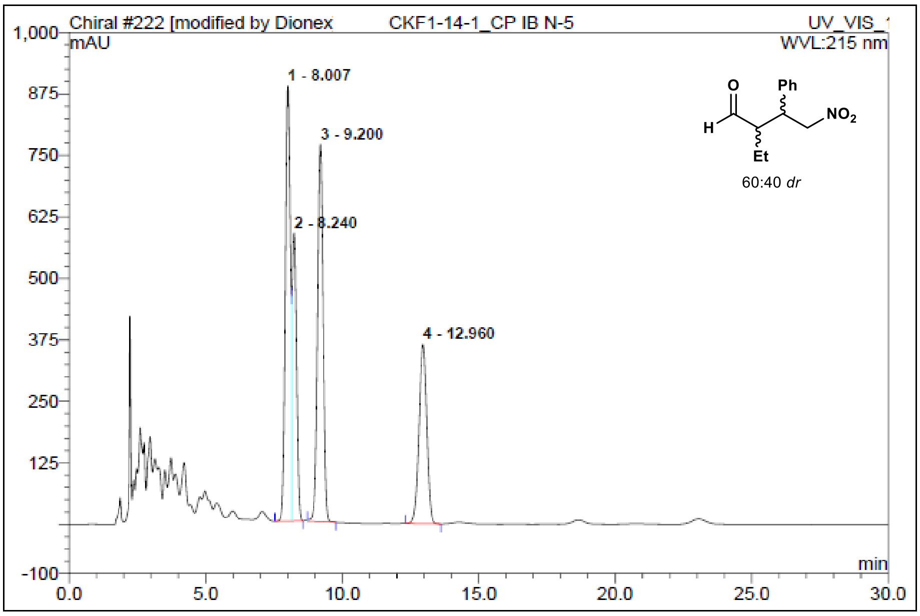

No.	Ret.Time min		Peak Name	Height mAU	Area mAU*min	Rel.Area %	Amount	Type
1	8.01	n.a.		884.993	183.833	30.83	n.a.	BM
2	8.24	n.a.		585.560	110.574	18.55	n.a.	MB
3	9.20	n.a.		766.589	181.280	30.41	n.a.	MB*
4	12.96	n.a.		364.683	120.523	20.21	n.a.	BMB*
Total:				2601.825	596.210	100.00	0.000	

9a:

^{1}H NMR (600 MHz, $CDCl_3$) δ 9.71 (d, *J* = 2.8 Hz, 1H), 7.37 – 7.32 (m, 2H), 7.32 – 7.27 (m, 1H), 7.19 – 7.15 (m, 2H), 4.70 (dd, *J* = 12.8, 5.2 Hz, 1H), 4.65 (dd, *J* = 12.8, 9.6 Hz, 1H), 3.78 (td, *J* = 9.6, 5.2 Hz, 1H), 2.74 – 2.67 (m, 1H), 1.53 – 1.44 (m, 1H), 1.40 – 1.29 (m, 2H), 1.25 – 1.13 (m, 1H), 0.80 (t, *J* = 7.1 Hz, 3H).

^{13}C NMR (151 MHz, $CDCl_3$) δ 203.4, 137.0, 129.3, 129.3, 128.3, 128.2, 78.6, 54.0, 43.4, 29.7, 20.0, 14.1.

Enantiomeric ratio was determined by chiral HPLC: Daicel Chiralpak IB N-5 column, hexane:isopropanol = 80:20, 1 mL/min flow rate, 215 nm detection wavelength. t_R (major, syn) = 9.6 min, t_R (minor, syn) = 7.5 min, t_R (anti) = 8.4 min, 13.4 min.

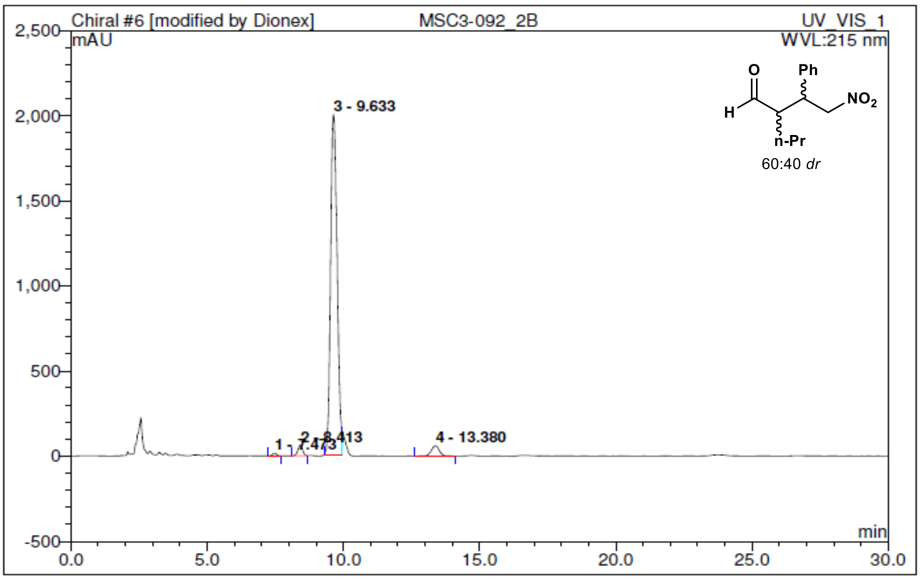

No.	Ret.Time min	Peak Name	Height mAU	Area mAU*min	Rel.Area %	Amount	Type
1	7.47	n.a.	15.589	2.676	0.45	n.a.	BMB*
2	8.41	n.a.	62.087	12.725	2.16	n.a.	BMB
3	9.63	n.a.	2001.131	553.813	93.93	n.a.	M *
4	13.38	n.a.	60.571	20.363	3.45	n.a.	BMB
Total:			2139.377	589.577	100.00	0.000	

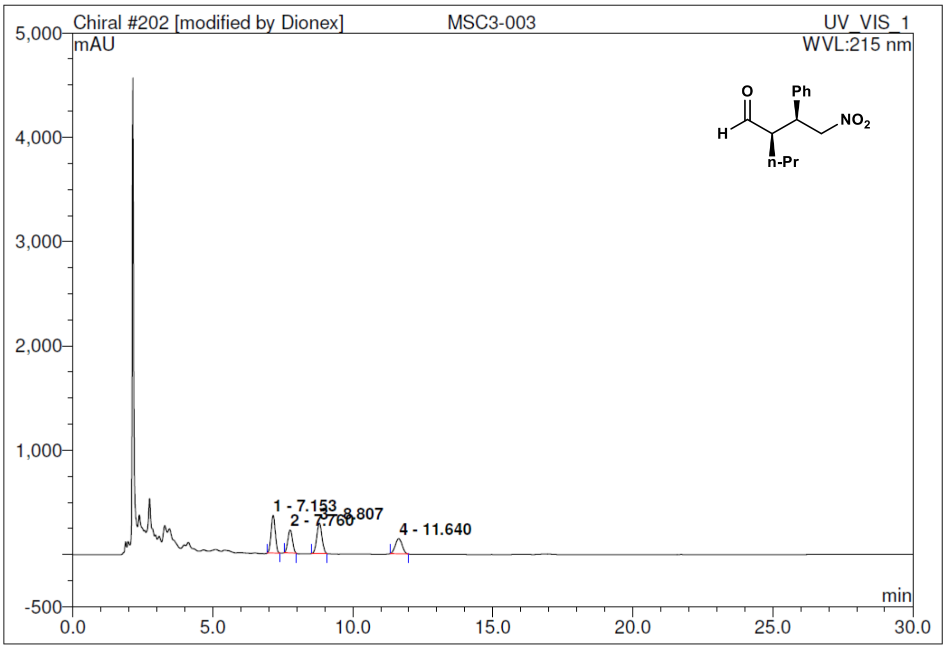

No.	Ret.Time min	Peak Name	Height mAU	Area mAU*min	Rel.Area %	Amount	Type
1	7.15	n.a.	360.112	63.389	30.37	n.a.	BMB*
2	7.76	n.a.	218.929	41.016	19.65	n.a.	BMB*
3	8.81	n.a.	291.155	63.317	30.33	n.a.	BMB*
4	11.64	n.a.	145.455	41.023	19.65	n.a.	BMB*
Total:			1015.652	208.745	100.00	0.000	

9c:

^{1}H NMR (600 MHz, $CDCl_3$) δ 9.72 (d, *J* = 1.8 Hz, 1H), 7.36 (d, *J* = 1.9 Hz, 1H), 6.31 (dd, *J* = 3.3, 1.9 Hz, 1H), 6.20 (d, *J* = 3.2 Hz, 1H), 4.70 (dd, *J* = 12.8, 9.2 Hz, 1H), 4.66 (dd, *J* = 12.8, 5.2 Hz, 1H), 4.01 (td, *J* = 8.8, 5.1 Hz, 1H), 2.81 – 2.70 (m, 1H), 1.60 – 1.53 (m, 2H), 0.90 (t, *J* = 7.5 Hz, 3H).

^{13}C NMR (151 MHz, $CDCl_3$) δ 202.5, 150.3, 142.9, 110.6, 109.0, 76.3, 53.6, 36.8, 20.2, 11.1.

Enantiomeric ratio was determined by chiral HPLC: Daicel Chiralpak IC column, hexane:isopropanol = 95:05, 1 mL/min flow rate, 215 nm detection wavelength. t_R (major, *syn*) = 45.5 min, t_R (minor, *syn*) = 33.2 min, t_R (*anti*) = 38.8 min, 42.4 min

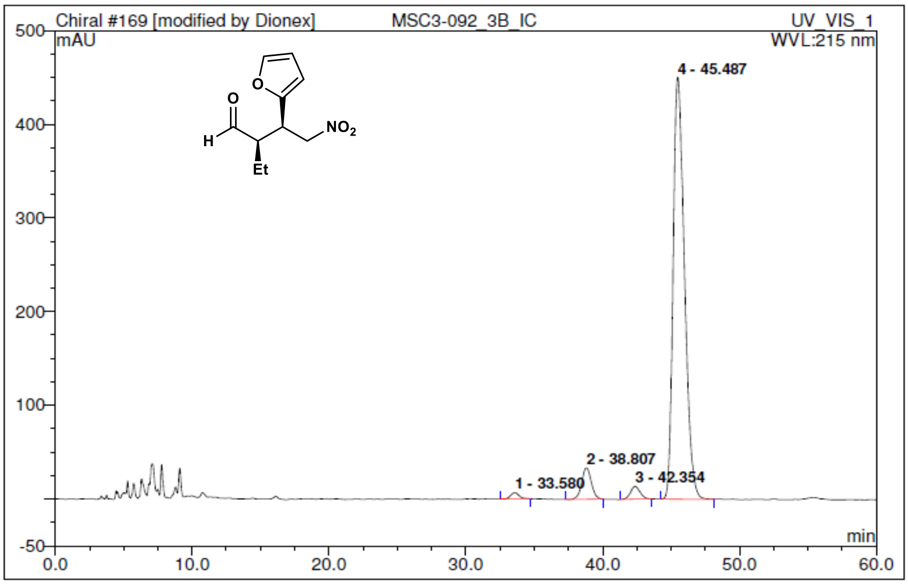

No.	Ret.Time min	Peak Name	Height mAU	Area mAU*min	Rel.Area %	Amount	Type
1	33.58	n.a.	6.734	4.766	1.00	n.a.	BMB*
2	38.81	n.a.	33.512	27.148	5.72	n.a.	BMB*
3	42.35	n.a.	13.699	11.068	2.33	n.a.	BMB*
4	45.49	n.a.	450.568	431.829	90.95	n.a.	BMB
Total:			504.514	474.812	100.00	0.000	

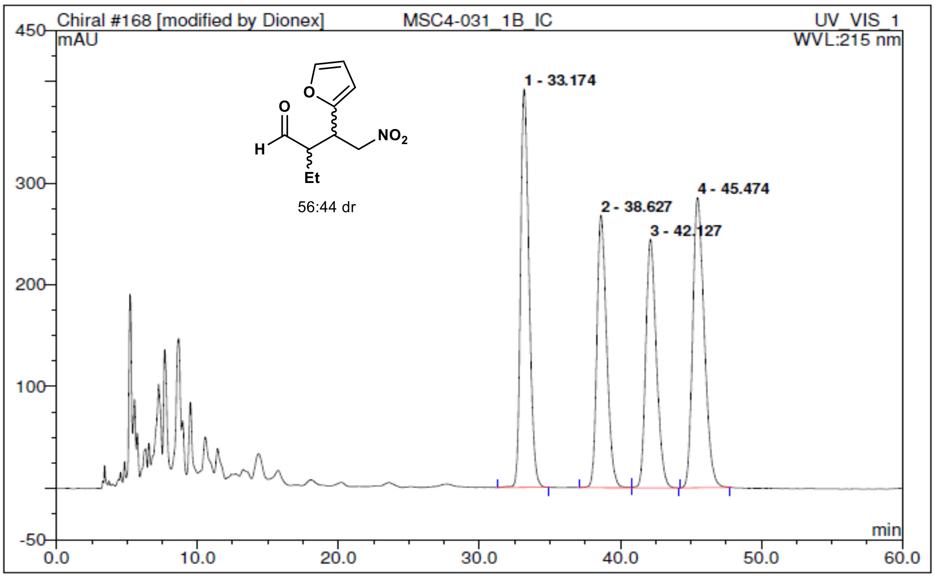

No.	Ret.Time min	Peak Name	Height mAU	Area mAU*min	Rel.Area %	Amount	Type
1	33.17	n.a.	391.480	272.860	28.09	n.a.	BMB
2	38.63	n.a.	267.561	214.709	22.10	n.a.	BM
3	42.13	n.a.	244.083	212.704	21.90	n.a.	MB
4	45.47	n.a.	285.111	271.136	27.91	n.a.	BMB
Total:			1188.235	971.409	100.00	0.000	

9d:

^{1}H NMR (600 MHz, $CDCl_3$) δ 9.71 (d, *J* = 1.9 Hz, 1H), 7.36 (d, *J* = 1.8 Hz, 1H), 6.31 (dd, *J* = 3.2, 1.9 Hz, 1H), 6.19 (d, *J* = 3.3 Hz, 1H), 4.71 (dd, *J* = 12.9, 9.3 Hz, 1H), 4.65 (dd, *J* = 12.8, 5.1 Hz, 1H), 4.00 (ddd, *J* = 9.2, 8.0, 5.1 Hz, 1H), 2.78 (tdd, *J* = 8.3, 4.2, 2.0 Hz, 1H), 1.60 – 1.48 (m, 1H), 1.45 – 1.32 (m, 2H), 1.27 – 1.18 (m, 1H), 0.88 (t, *J* = 7.2 Hz, 3H).

^{13}C NMR (151 MHz, $CDCl_3$) δ 202.5, 150.3, 142.8, 110.7, 108.9, 76.2, 52.3, 37.2, 29.2, 20.2, 14.2.

Enantiomeric ratio was determined by chiral HPLC after reduction to the corresponding alcohol*: Daicel Chiralpak IC column, hexane:isopropanol = 98:02, 1 mL/min flow rate, 215 nm detection wavelength. t_R (major, *syn*) = 73.5 min, t_R (minor, *syn*) = 80.8 min, t_R (*anti*) = 68.7 min, 88.0 min

*The reduction was carried out as follows: aldehyde (0.25 mmol) was dissolved in MeOH (2.5 mL) and excess sodium borohydride (1.00 mmol, 4.0 eq.) was added. The reaction was stirred for 30 mins, then quenched with aq. NH_4Cl (10 mL), and the product was extracted with CH_2Cl_2 (2 x 10 mL). The combined organics were dried, and the solvent was removed *in vacuo*.

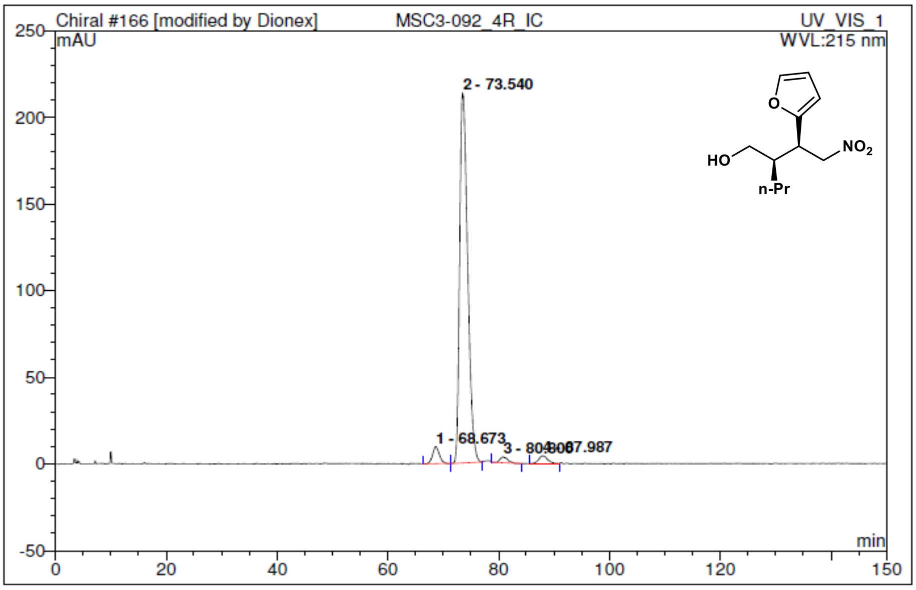

No.	Ret.Time min	Peak Name	Height mAU	Area mAU*min	Rel.Area %	Amount	Type
1	68.67	n.a.	9.913	13.993	3.66	n.a.	BMB*
2	73.54	n.a.	213.679	355.034	92.88	n.a.	BMB
3	80.80	n.a.	3.337	5.271	1.38	n.a.	BMB*
4	87.99	n.a.	4.400	7.934	2.08	n.a.	BMB*
Total:			231.330	382.232	100.00	0.000	

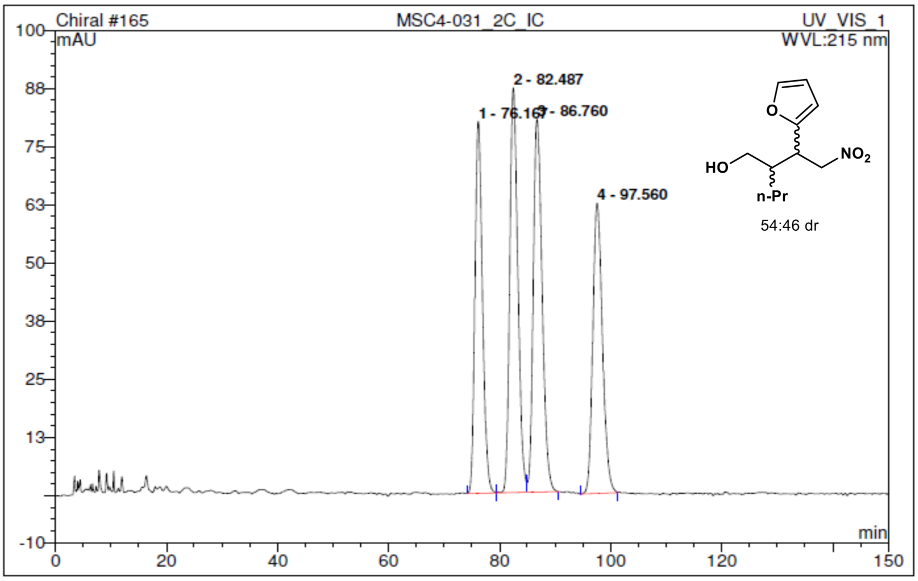

No.	Ret.Time min	Peak Name	Height mAU	Area mAU*min	Rel.Area %	Amount	Type
1	76.17	n.a.	79.966	126.370	23.01	n.a.	BM
2	82.49	n.a.	87.013	148.116	26.97	n.a.	M
3	86.76	n.a.	80.112	148.668	27.07	n.a.	MB
4	97.56	n.a.	62.370	125.979	22.94	n.a.	BMB
Total:			309.462	549.132	100.00	0.000	

Synthesis of racemates

All racemic samples were prepared as follows:

To a solution of the corresponding nitrostyrene (5.0 mmol) in CH_2Cl_2 (5.0 mL) was added the corresponding aldehyde (10.0 mmol, 2.0 eq.), and pyrrolidine (82 µL, 1 mmol, 0.2 eq.), and the mixture was stirred at room temperature for 16 hours. The solvent was removed *in vacuo* and the residue was purified by column chromatography (10→20% EtOAc/PE) to afford ***rac*-9a-d** as a mixture of diastereoisomers.

***Rac*-9a** (60:40 *dr*). The sample also contained a considerable amount of homoaldol condensation product which co-eluted with the product.

***Rac*-9b** (60:40 *dr*). The sample also contained a considerable amount of homoaldol condensation product which co-eluted with the product.

***Rac*-9c** pale-yellow oil (555 mg, 2.63 mmol, 53%, 56:44 *dr).* Major diastereoisomer is the same as in the synthesis of **9c**.

***Rac*-9d** yellow oil (483 mg, 2.15 mmol, 43%, 54:46 *dr).* Major diastereoisomer is the same as in the synthesis of **9d**.

NMR spectra

SI-1

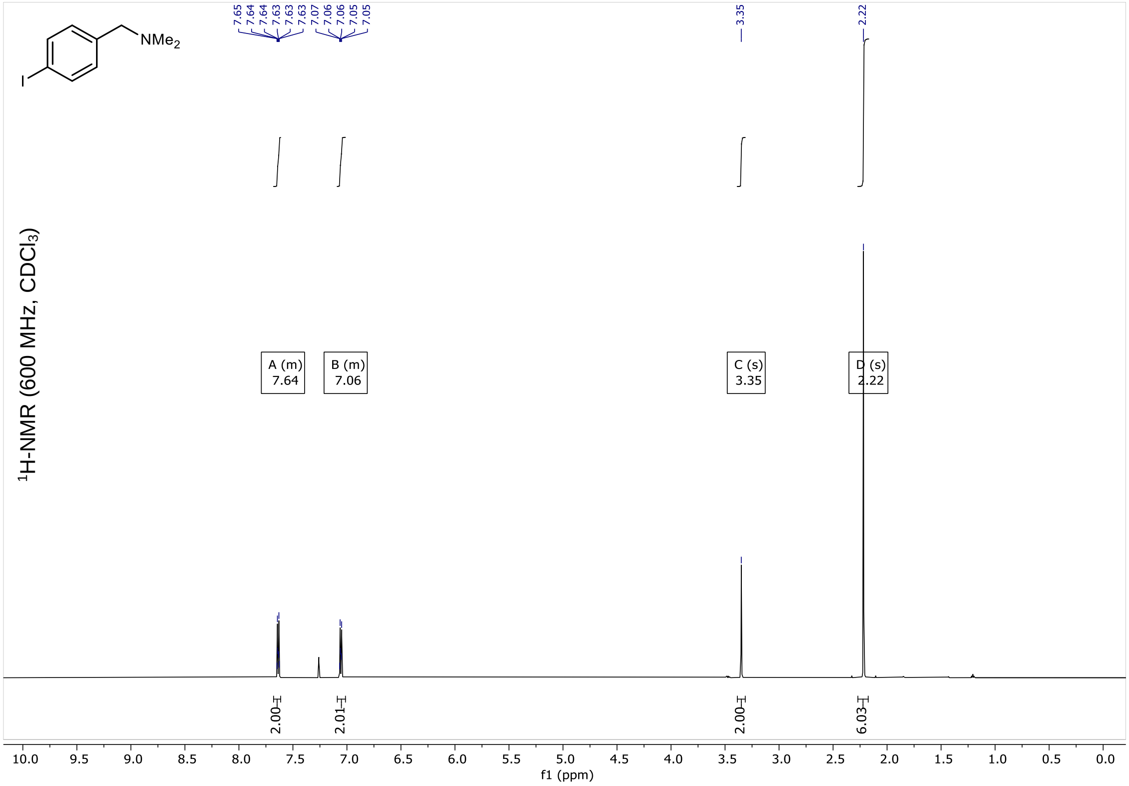

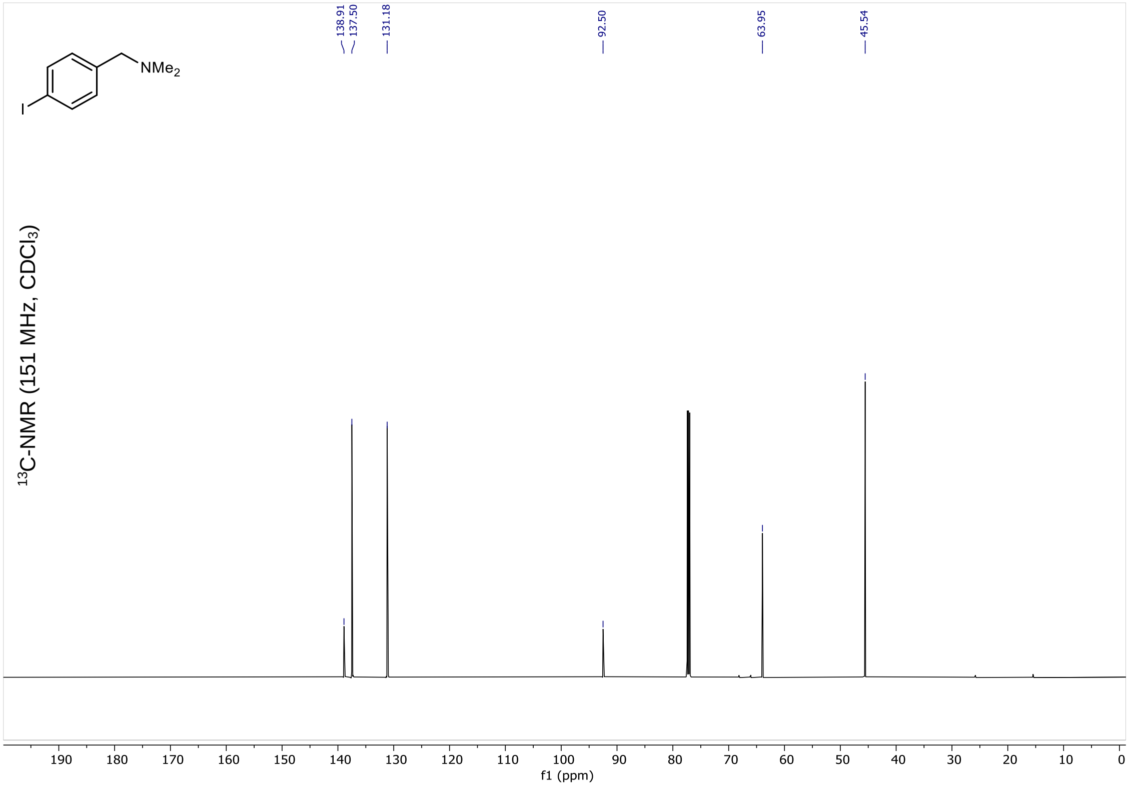

SI-2

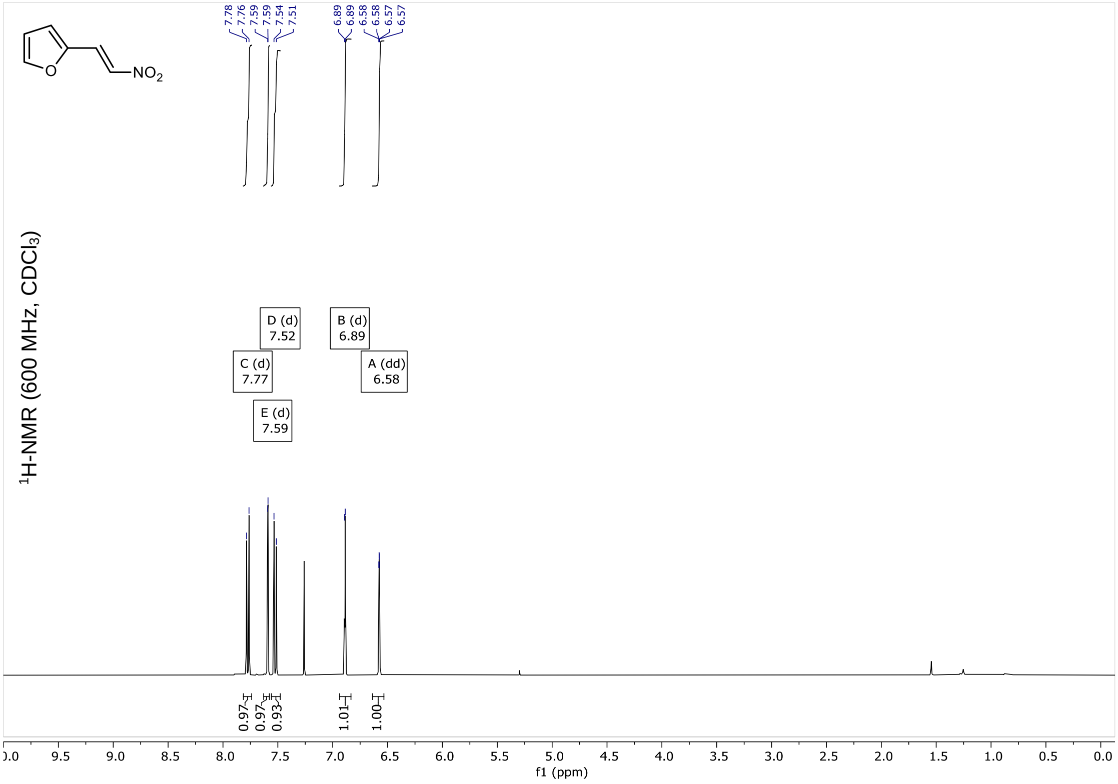
7.78
7.76
7.59
7.59
7.54
7.51
6.89
6.89
6.58
6.58
6.57
6.57
NO2
O
1H-NMR (600 MHz, CDCl3)
D (d) 7.52
B (d) 6.89
C (d) 7.77
A (dd) 6.58
E (d) 7.59
0.97
0.97
0.93
1.01
1.00
f1 (ppm)

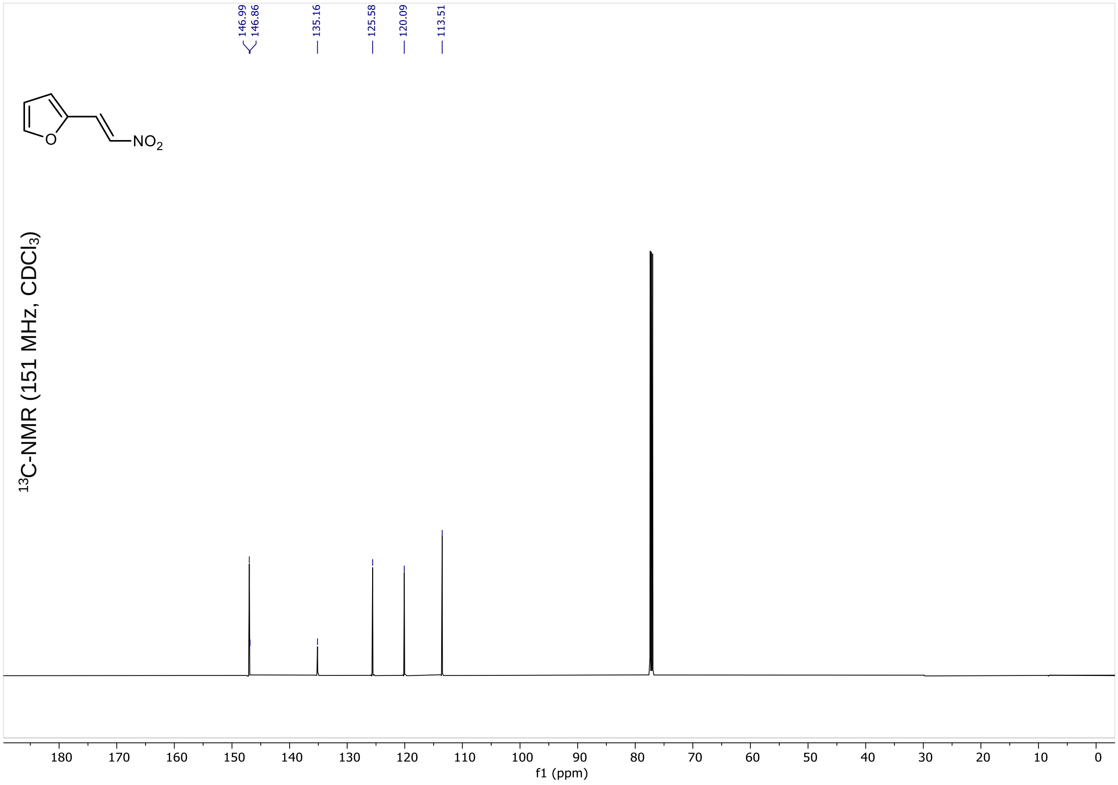
146.99
146.86
135.16
125.58
120.09
113.51
NO2
O
13C-NMR (151 MHz, CDCl3)
f1 (ppm)

(S)-Cat-1

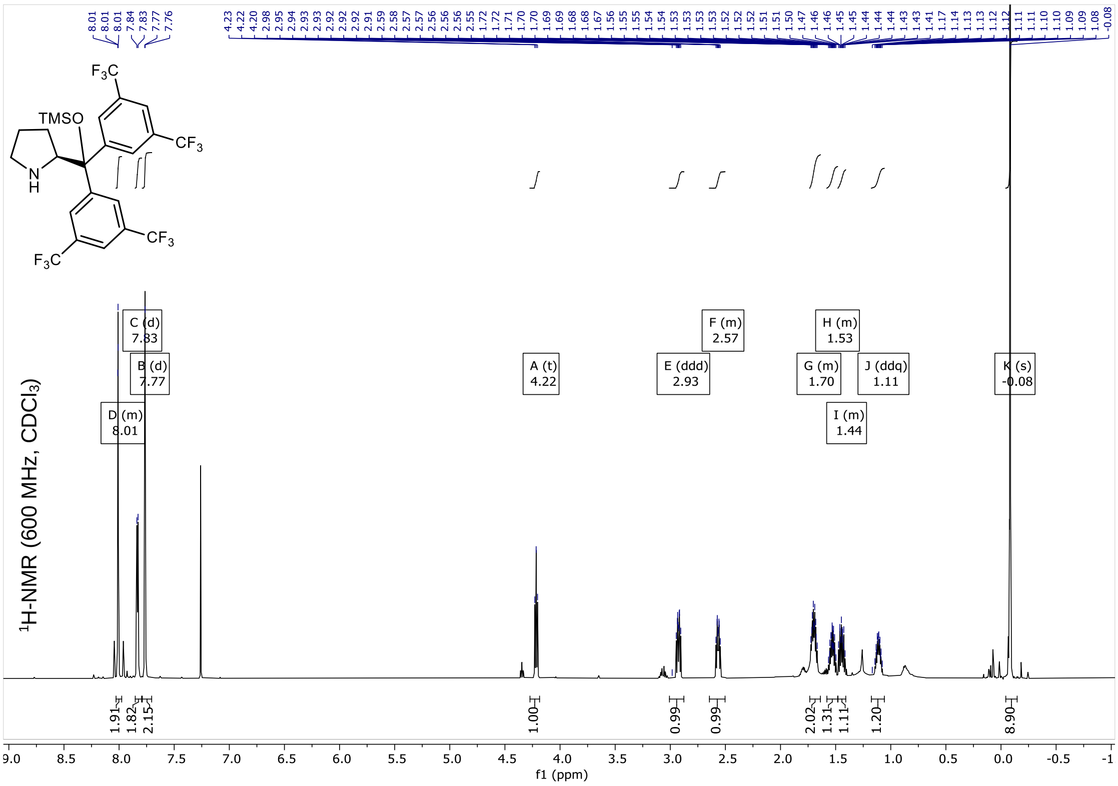

^{1}H-NMR (600 MHz, CDCl$_3$)
TMSO
N
H
F$_3$C
CF$_3$
A (t) 4.22
B (d) 7.77
C (d) 7.83
D (m) 8.01
E (ddd) 2.93
F (m) 2.57
G (m) 1.70
H (m) 1.53
I (m) 1.44
J (ddq) 1.11
K (s) -0.08
1.91
1.82
2.15
1.00
0.99
0.99
2.02
1.31
1.11
1.20
8.90
f1 (ppm)

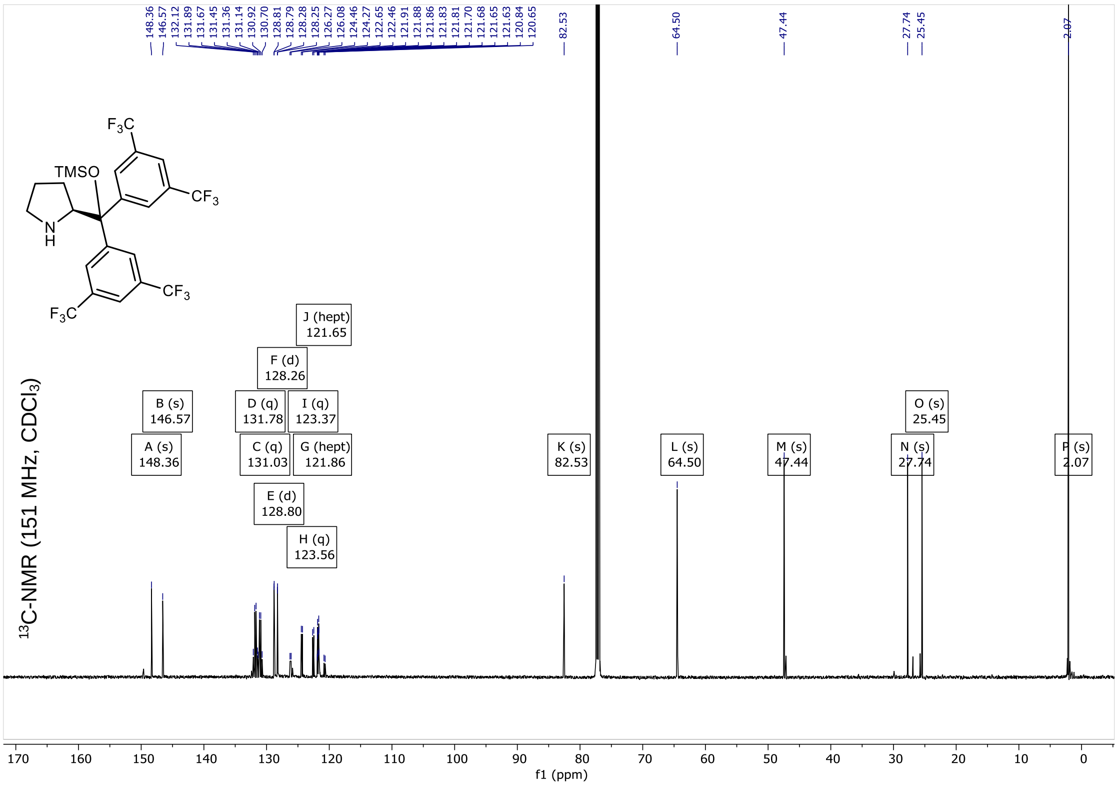

^{13}C-NMR (151 MHz, CDCl$_3$)
TMSO
N
H
F$_3$C
CF$_3$
A (s) 148.36
B (s) 146.57
C (q) 131.03
D (q) 131.78
E (d) 128.80
F (d) 128.26
G (hept) 121.86
H (q) 123.56
I (q) 123.37
J (hept) 121.65
K (s) 82.53
L (s) 64.50
M (s) 47.44
N (s) 27.74
O (s) 25.45
P (s) 2.07
f1 (ppm)

(S)-Cat-2

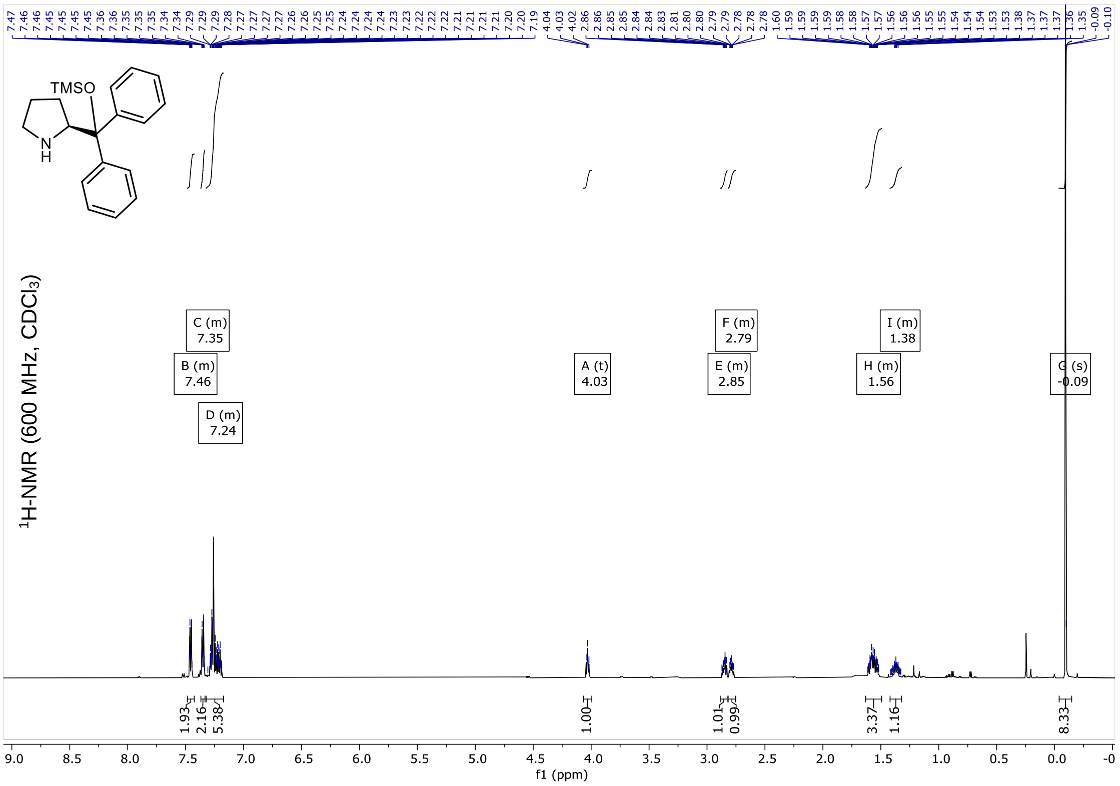

1H-NMR (600 MHz, CDCl3)
TMSO
N
H
C (m) 7.35
B (m) 7.46
D (m) 7.24
A (t) 4.03
F (m) 2.79
E (m) 2.85
I (m) 1.38
H (m) 1.56
G (s) -0.09
1.93
2.16
5.38
1.00
1.01
0.99
3.37
1.16
8.33
f1 (ppm)

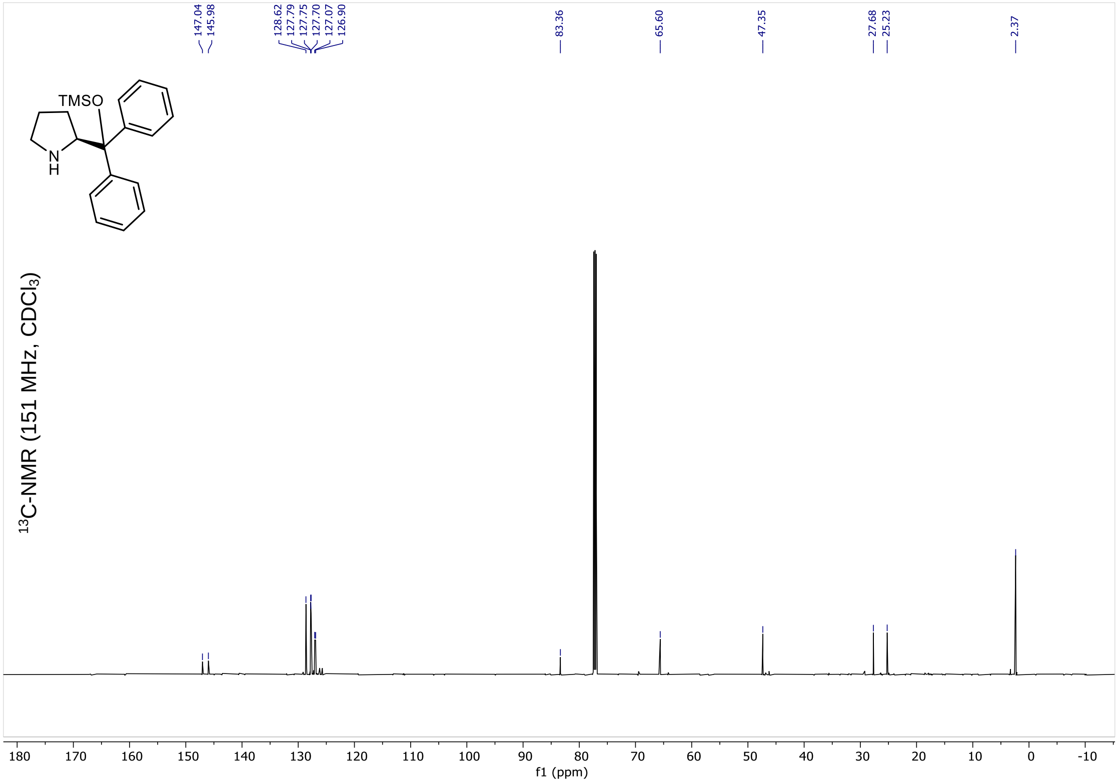

13C-NMR (151 MHz, CDCl3)
147.04
145.98
128.62
127.79
127.75
127.70
127.07
126.90
83.36
65.60
47.35
27.68
25.23
2.37
TMSO
N
H
f1 (ppm)

(S)-Cat-3

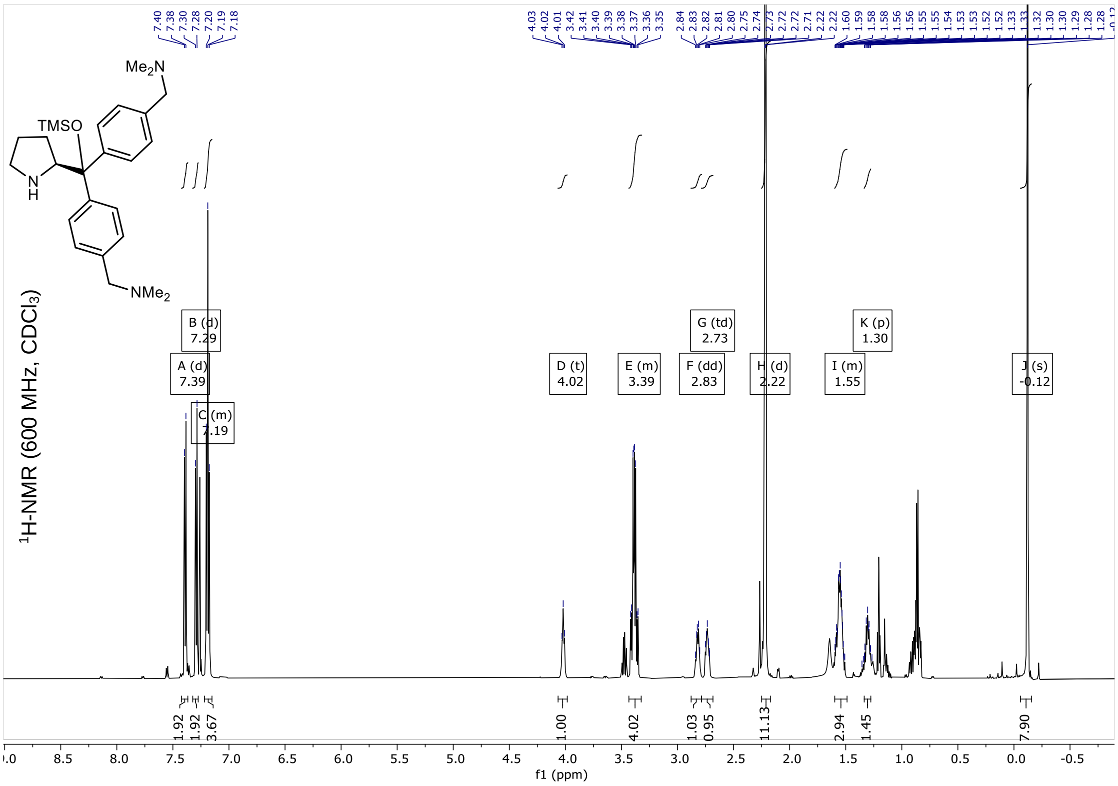
¹H-NMR (600 MHz, CDCl₃)
Me₂N
TMSO
N
H
NMe₂
A (d)
7.39
B (d)
7.29
C (m)
7.19
D (t)
4.02
E (m)
3.39
F (dd)
2.83
G (td)
2.73
H (d)
2.22
I (m)
1.55
K (p)
1.30
J (s)
-0.12
1.92
1.92
3.67
1.00
4.02
1.03
0.95
11.13
2.94
1.45
7.90
f1 (ppm)

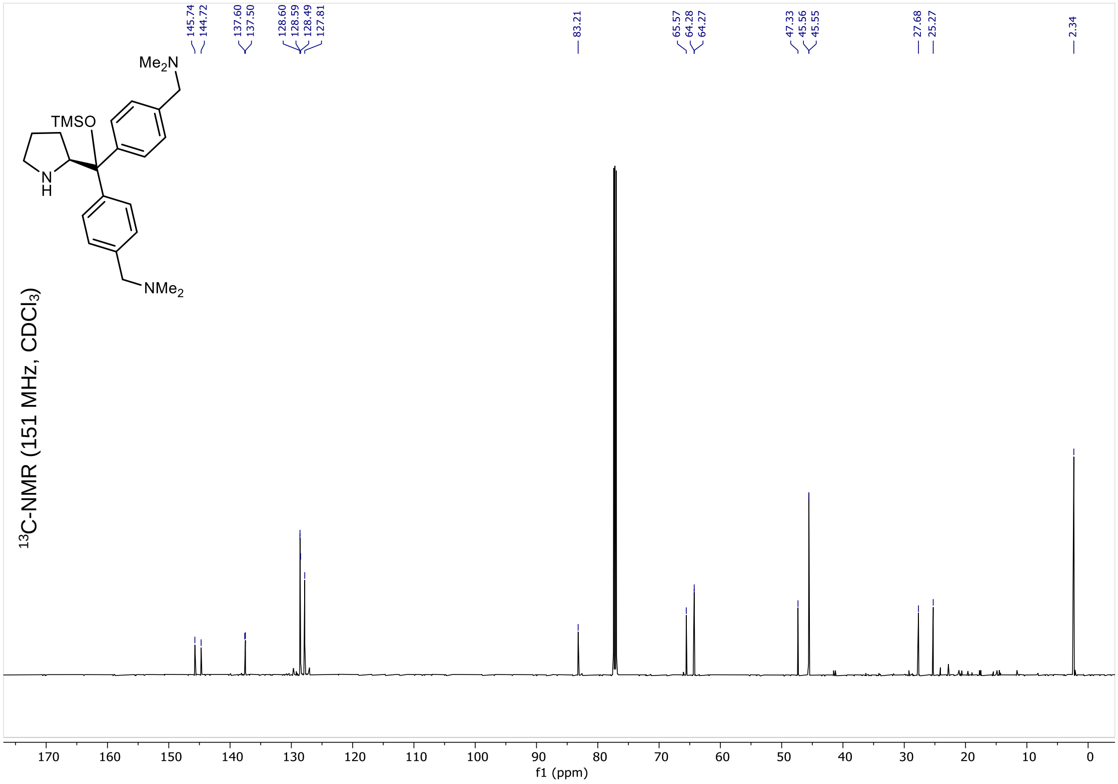
¹³C-NMR (151 MHz, CDCl₃)
Me₂N
TMSO
N
H
NMe₂
145.74
144.72
137.60
137.50
128.60
128.59
128.49
127.81
83.21
65.57
64.28
64.27
47.33
45.56
45.55
27.68
25.27
2.34
f1 (ppm)

1a

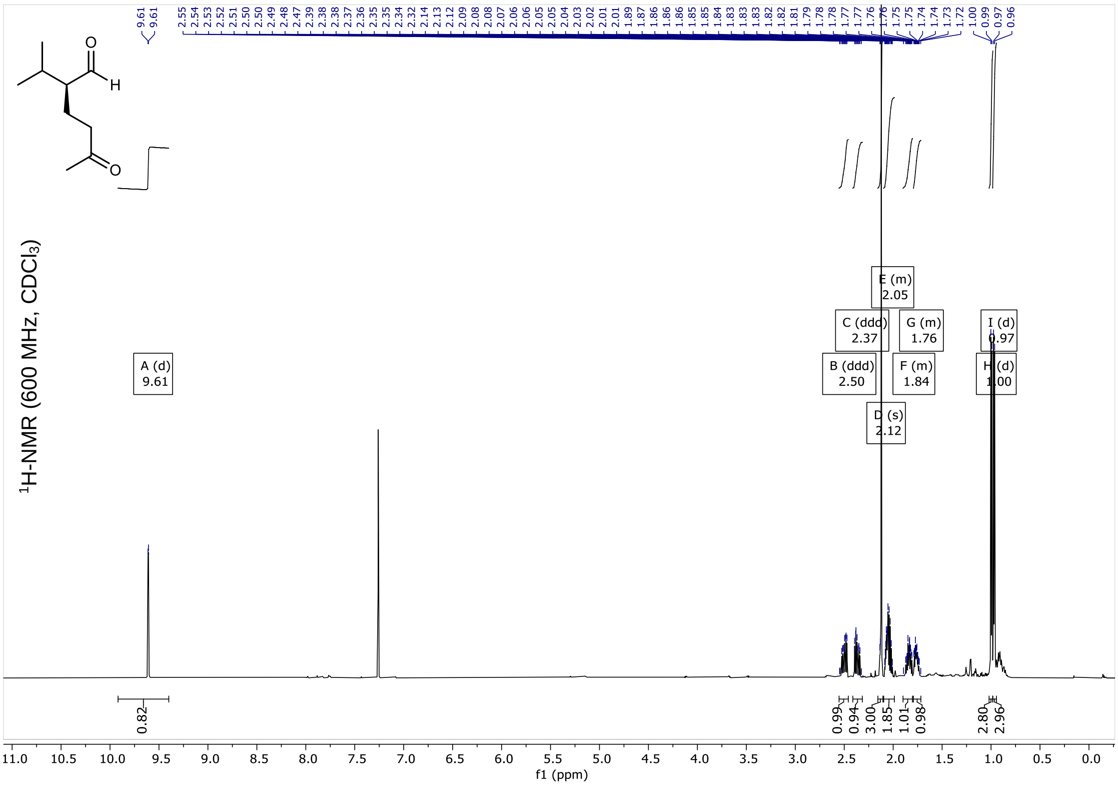

^{1}H-NMR (600 MHz, CDCl$_3$)
A (d) 9.61
B (ddd) 2.50
C (ddd) 2.37
D (s) 2.12
E (m) 2.05
F (m) 1.84
G (m) 1.76
H (d) 1.00
I (d) 0.97
0.82
0.99
0.94
3.00
1.85
1.01
0.98
2.80
2.96
f1 (ppm)

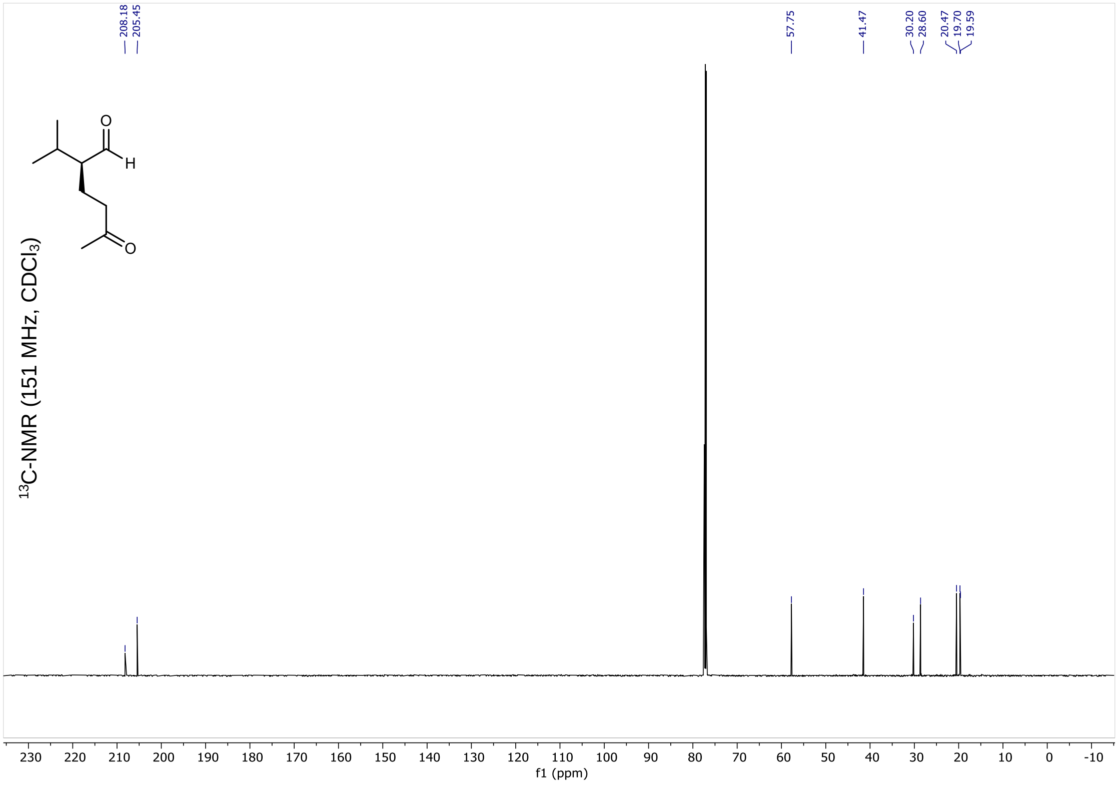

^{13}C-NMR (151 MHz, CDCl$_3$)
208.18
205.45
57.75
41.47
30.20
28.60
20.47
19.70
19.59
f1 (ppm)

1b

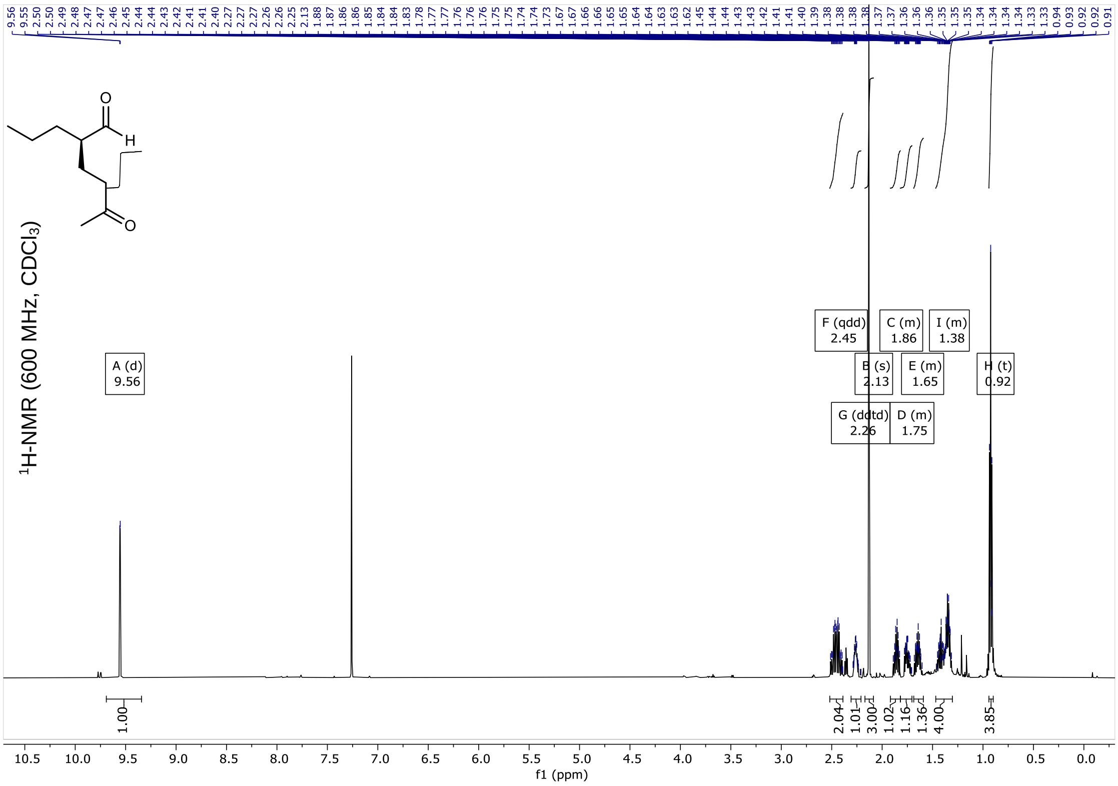
1H-NMR (600 MHz, CDCl3)
A (d) 9.56
F (qdd) 2.45
G (ddtd) 2.26
B (s) 2.13
C (m) 1.86
D (m) 1.75
E (m) 1.65
I (m) 1.38
H (t) 0.92
f1 (ppm)

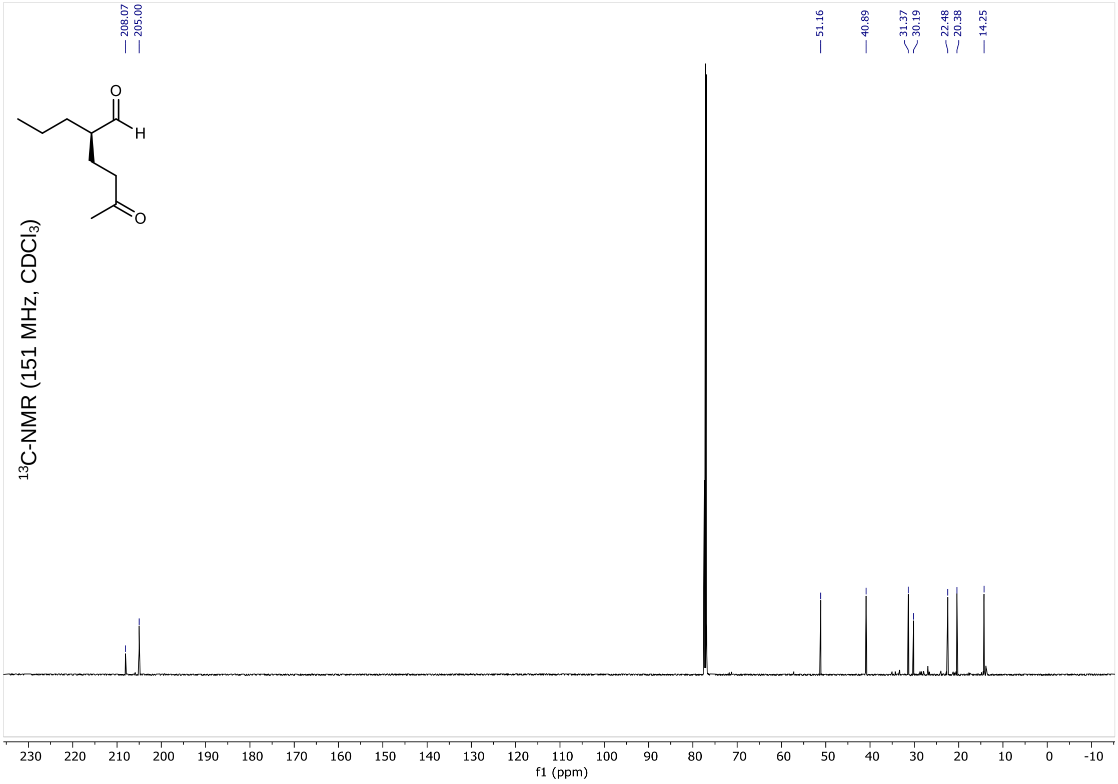
13C-NMR (151 MHz, CDCl3)
208.07
205.00
51.16
40.89
31.37
30.19
22.48
20.38
14.25
f1 (ppm)

2

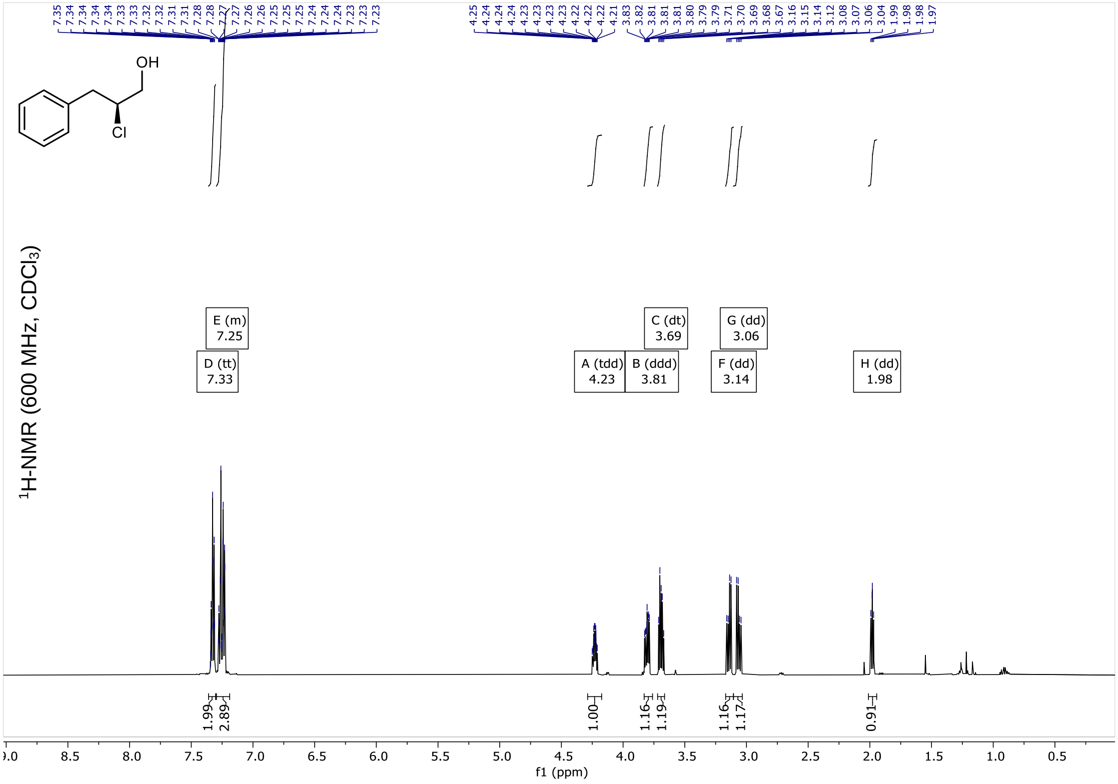

OH
Cl
1H-NMR (600 MHz, CDCl3)
E (m) 7.25
D (tt) 7.33
C (dt) 3.69
G (dd) 3.06
A (tdd) 4.23
B (ddd) 3.81
F (dd) 3.14
H (dd) 1.98
1.99
2.89
1.00
1.16
1.19
1.16
1.17
0.91
f1 (ppm)

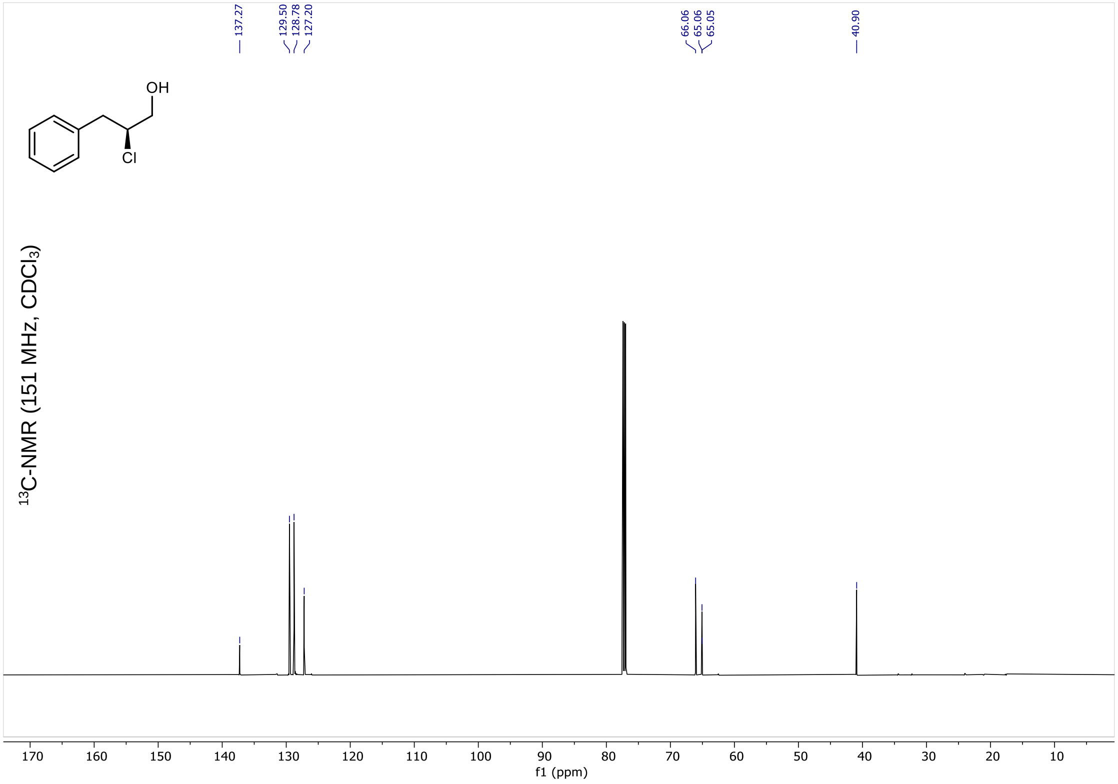

137.27
129.50
128.78
127.20
66.06
65.06
65.05
40.90
OH
Cl
13C-NMR (151 MHz, CDCl3)
f1 (ppm)

4

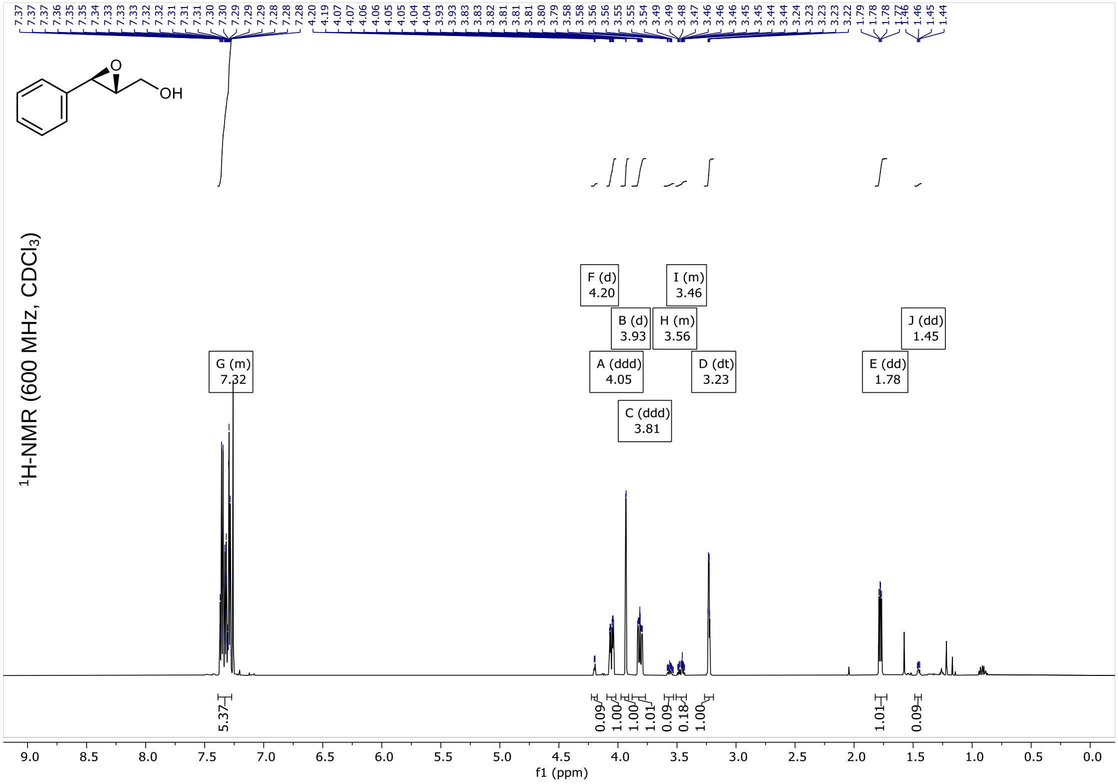
OH
1H-NMR (600 MHz, CDCl3)
G (m) 7.32
F (d) 4.20
B (d) 3.93
A (ddd) 4.05
C (ddd) 3.81
I (m) 3.46
H (m) 3.56
D (dt) 3.23
J (dd) 1.45
E (dd) 1.78
f1 (ppm)

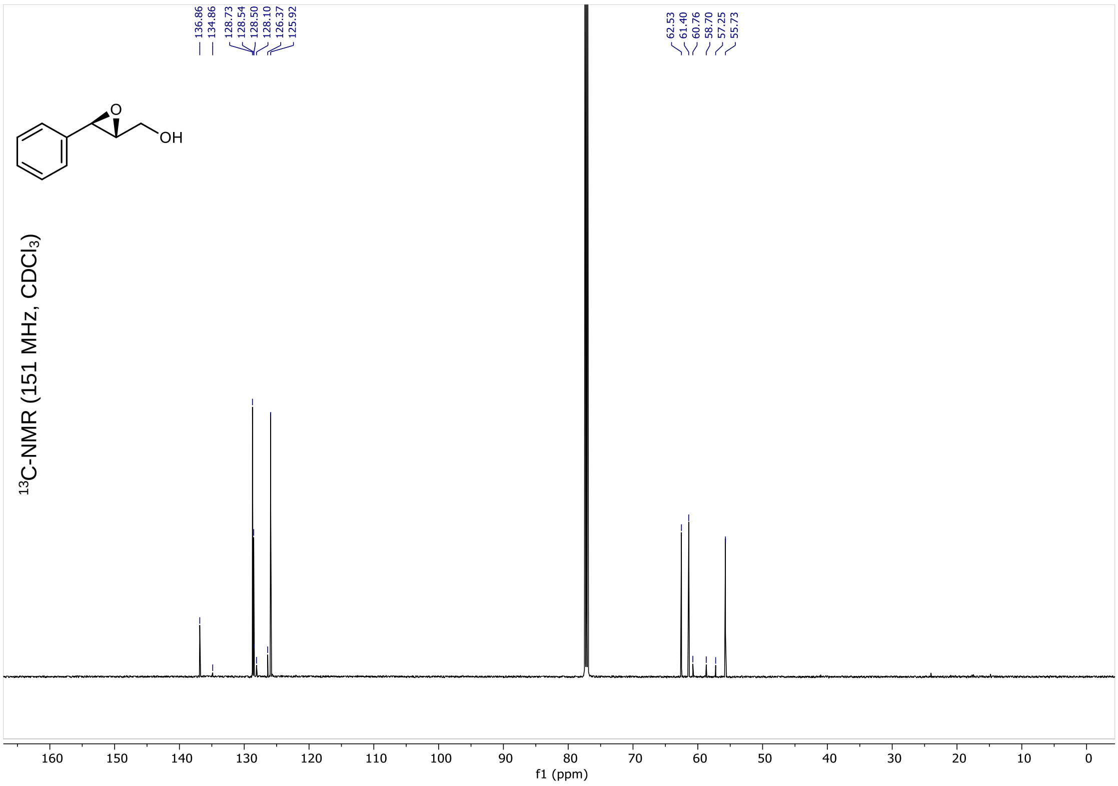
136.86
134.86
128.73
128.54
128.50
128.10
126.37
125.92
62.53
61.40
60.76
58.70
57.25
55.73
OH
13C-NMR (151 MHz, CDCl3)
f1 (ppm)

5

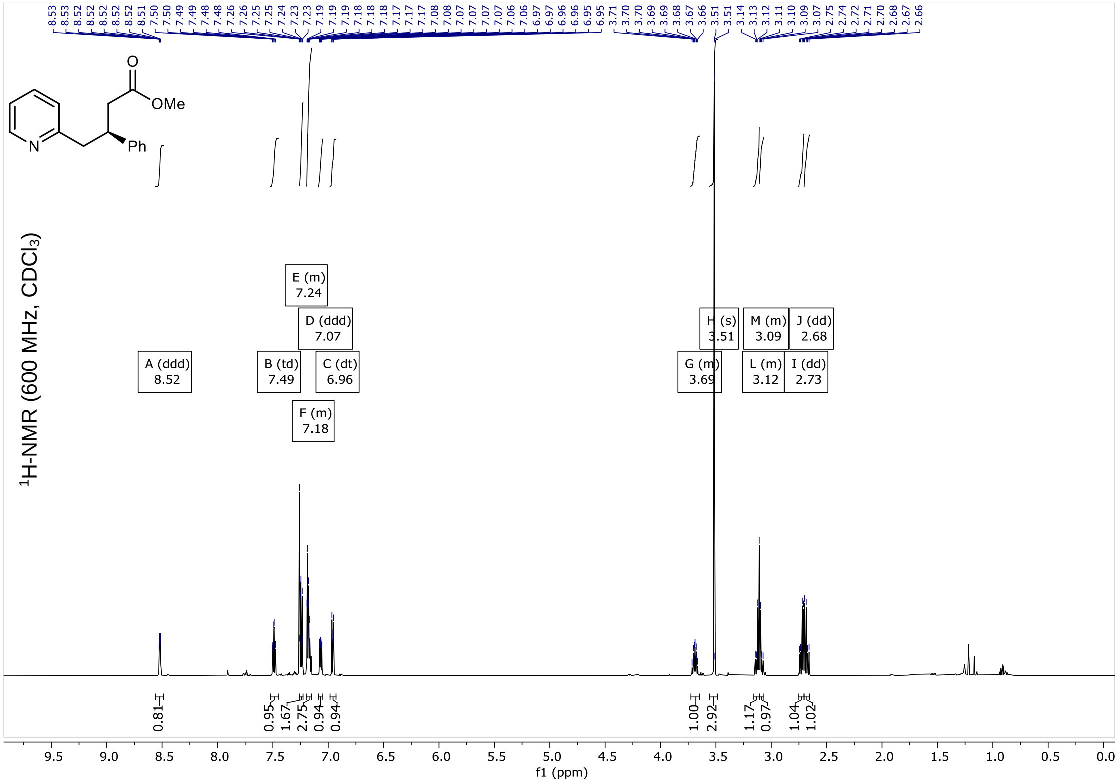

1H-NMR (600 MHz, CDCl3)
O
OMe
N
Ph
A (ddd) 8.52
B (td) 7.49
C (dt) 6.96
D (ddd) 7.07
E (m) 7.24
F (m) 7.18
G (m) 3.69
H (s) 3.51
L (m) 3.12
M (m) 3.09
I (dd) 2.73
J (dd) 2.68
0.81
0.95
1.67
2.75
0.94
0.94
1.00
2.92
1.17
0.97
1.04
1.02
f1 (ppm)

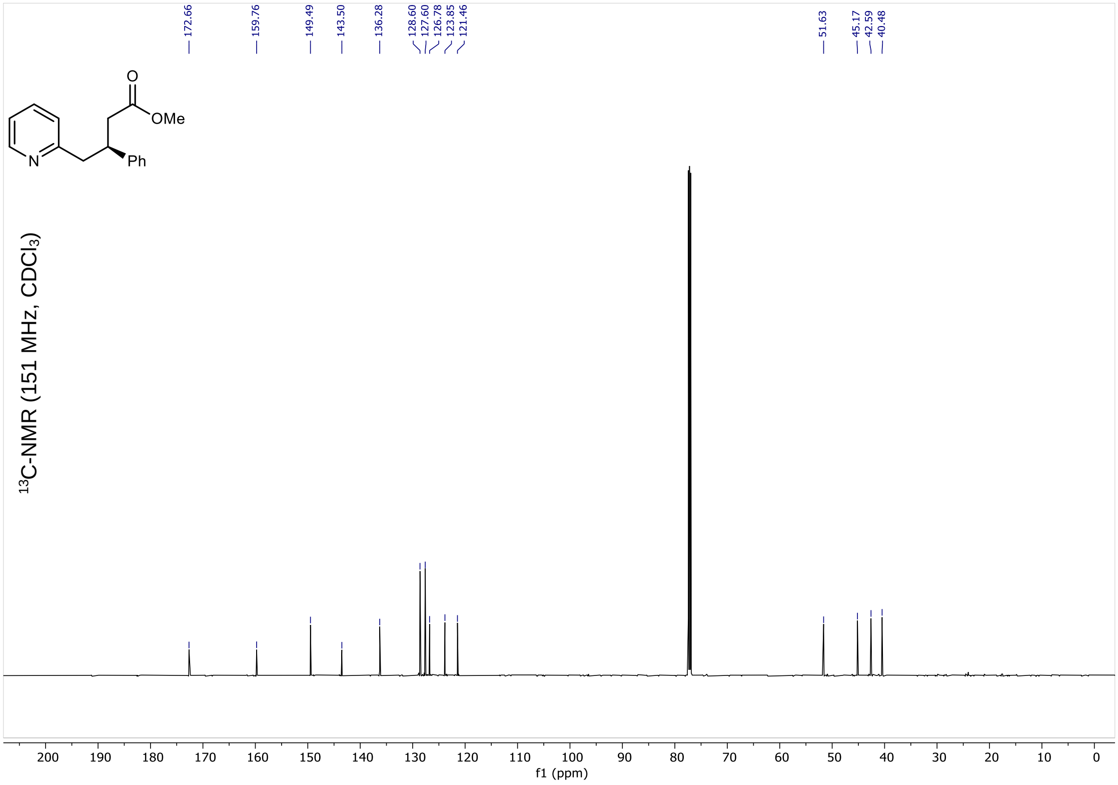

13C-NMR (151 MHz, CDCl3)
O
OMe
N
Ph
172.66
159.76
149.49
143.50
136.28
128.60
127.60
126.78
123.85
121.46
51.63
45.17
42.59
40.48
f1 (ppm)

6

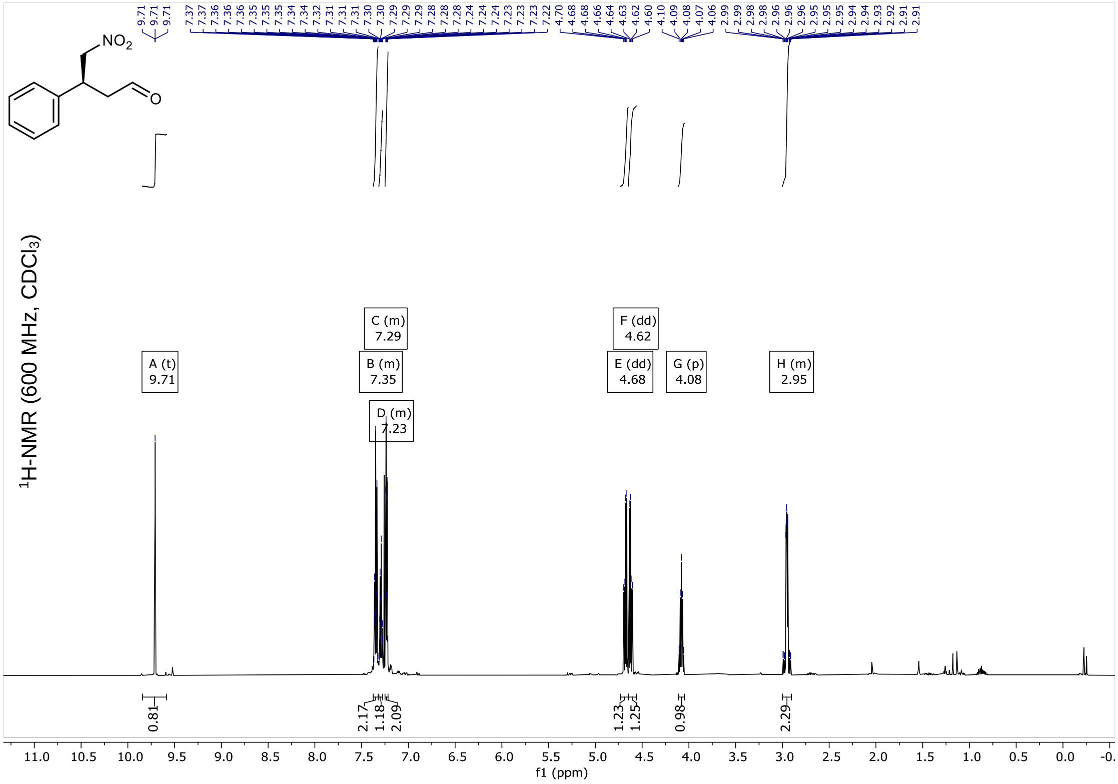

^{1}H-NMR (600 MHz, CDCl$_3$)
NO$_2$
O
9.71 9.71 9.71
7.37 7.37 7.36 7.36 7.35 7.35 7.35 7.34 7.34 7.32 7.31 7.31 7.31 7.30 7.30 7.29 7.29 7.29 7.28 7.28 7.28 7.24 7.24 7.24 7.23 7.23 7.23 7.22
4.70 4.68 4.68 4.66 4.64 4.63 4.62 4.60
4.10 4.09 4.08 4.07 4.06
2.99 2.99 2.98 2.98 2.96 2.96 2.96 2.95 2.95 2.95 2.94 2.94 2.93 2.92 2.91 2.91
A (t) 9.71
B (m) 7.35
C (m) 7.29
D (m) 7.23
E (dd) 4.68
F (dd) 4.62
G (p) 4.08
H (m) 2.95
0.81
2.17
1.18
2.09
1.23
1.25
0.98
2.29
11.0 10.5 10.0 9.5 9.0 8.5 8.0 7.5 7.0 6.5 6.0 5.5 5.0 4.5 4.0 3.5 3.0 2.5 2.0 1.5 1.0 0.5 0.0 -0.
f1 (ppm)

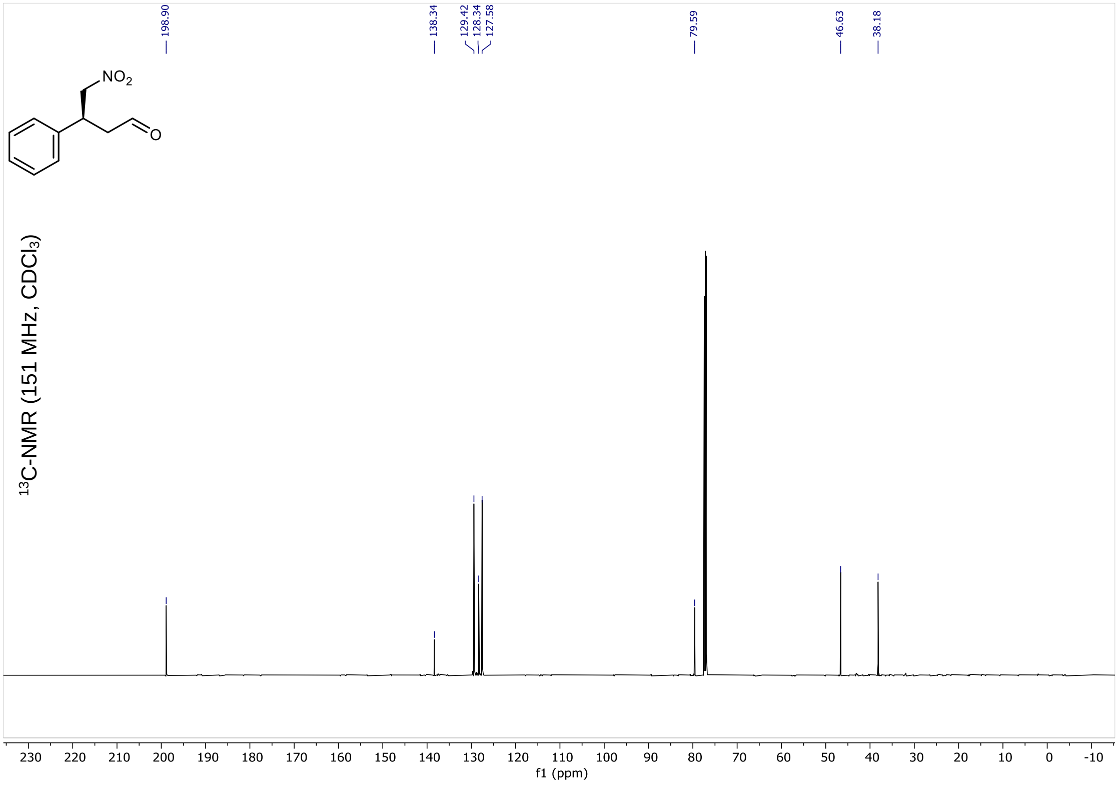

^{13}C-NMR (151 MHz, CDCl$_3$)
NO$_2$
O
198.90
138.34
129.42
128.34
127.58
79.59
46.63
38.18
230 220 210 200 190 180 170 160 150 140 130 120 110 100 90 80 70 60 50 40 30 20 10 0 -10
f1 (ppm)

7a

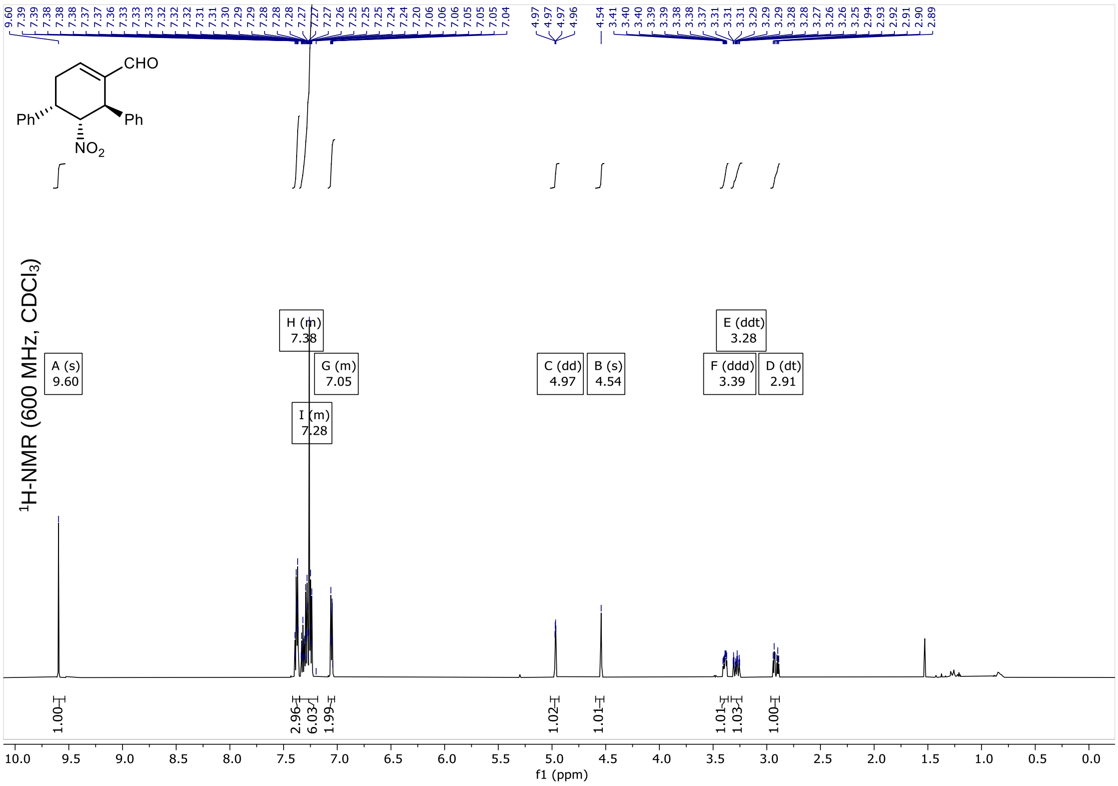
CHO
Ph
Ph
NO2
1H-NMR (600 MHz, CDCl3)
A (s) 9.60
H (m) 7.38
G (m) 7.05
I (m) 7.28
C (dd) 4.97
B (s) 4.54
E (ddt) 3.28
F (ddd) 3.39
D (dt) 2.91
1.00
2.96
6.03
1.99
1.02
1.01
1.01
1.03
1.00
f1 (ppm)

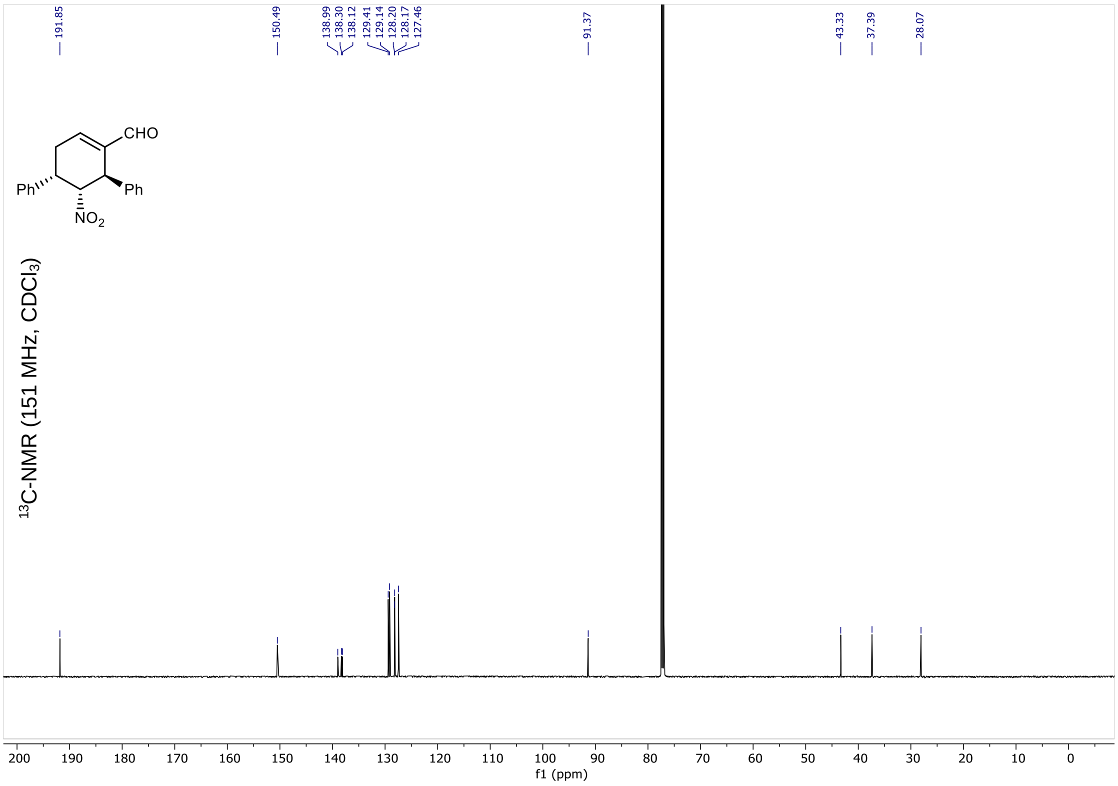
191.85
150.49
138.99
138.30
138.12
129.41
129.14
128.20
128.17
127.46
91.37
43.33
37.39
28.07
CHO
Ph
Ph
NO2
13C-NMR (151 MHz, CDCl3)
f1 (ppm)

7b

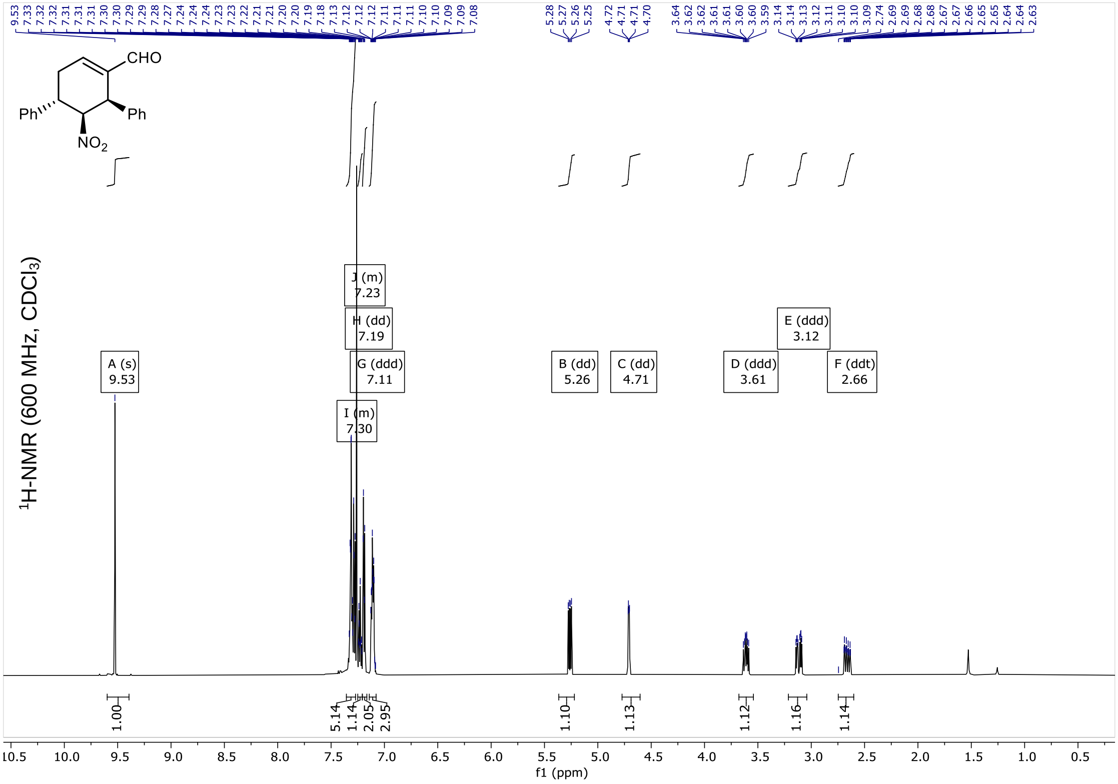

CHO
Ph
Ph
NO2
1H-NMR (600 MHz, CDCl3)
A (s) 9.53
J (m) 7.23
H (dd) 7.19
G (ddd) 7.11
I (m) 7.30
B (dd) 5.26
C (dd) 4.71
D (ddd) 3.61
E (ddd) 3.12
F (ddt) 2.66
1.00
5.14
1.14
2.05
2.95
1.10
1.13
1.12
1.16
1.14
f1 (ppm)

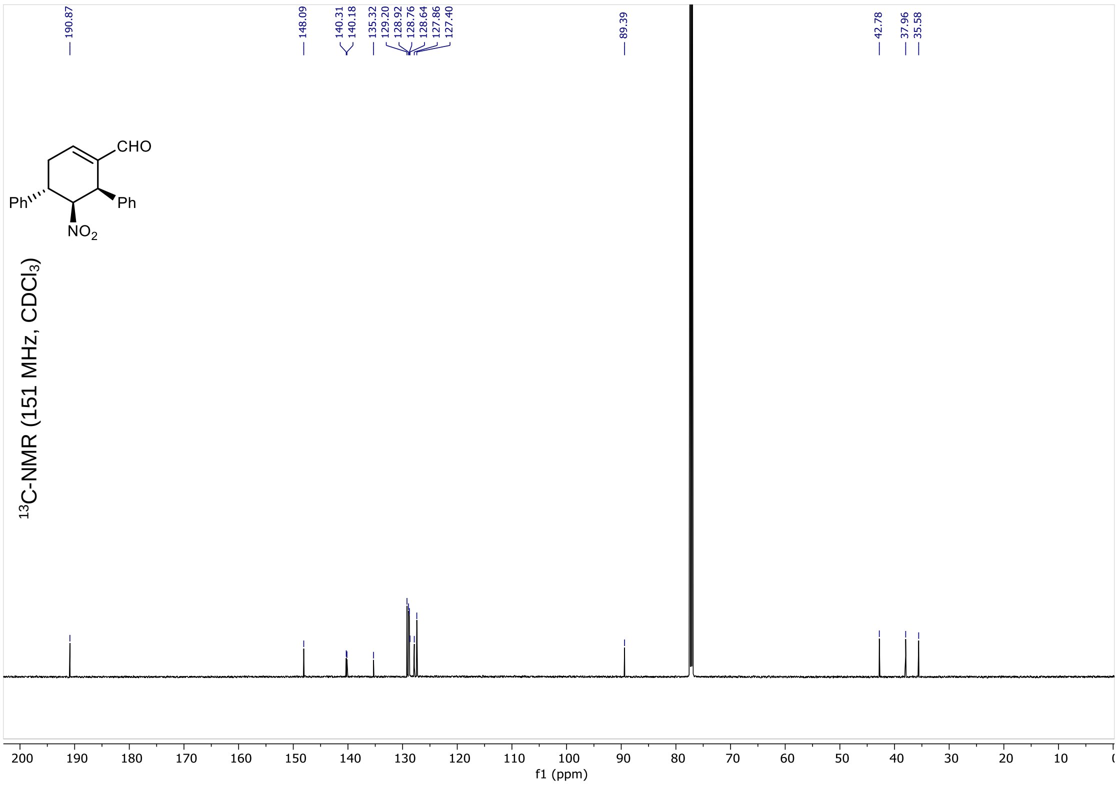

190.87
148.09
140.31
140.18
135.32
129.20
128.92
128.76
128.64
127.86
127.40
89.39
42.78
37.96
35.58
CHO
Ph
Ph
NO2
13C-NMR (151 MHz, CDCl3)
f1 (ppm)

8

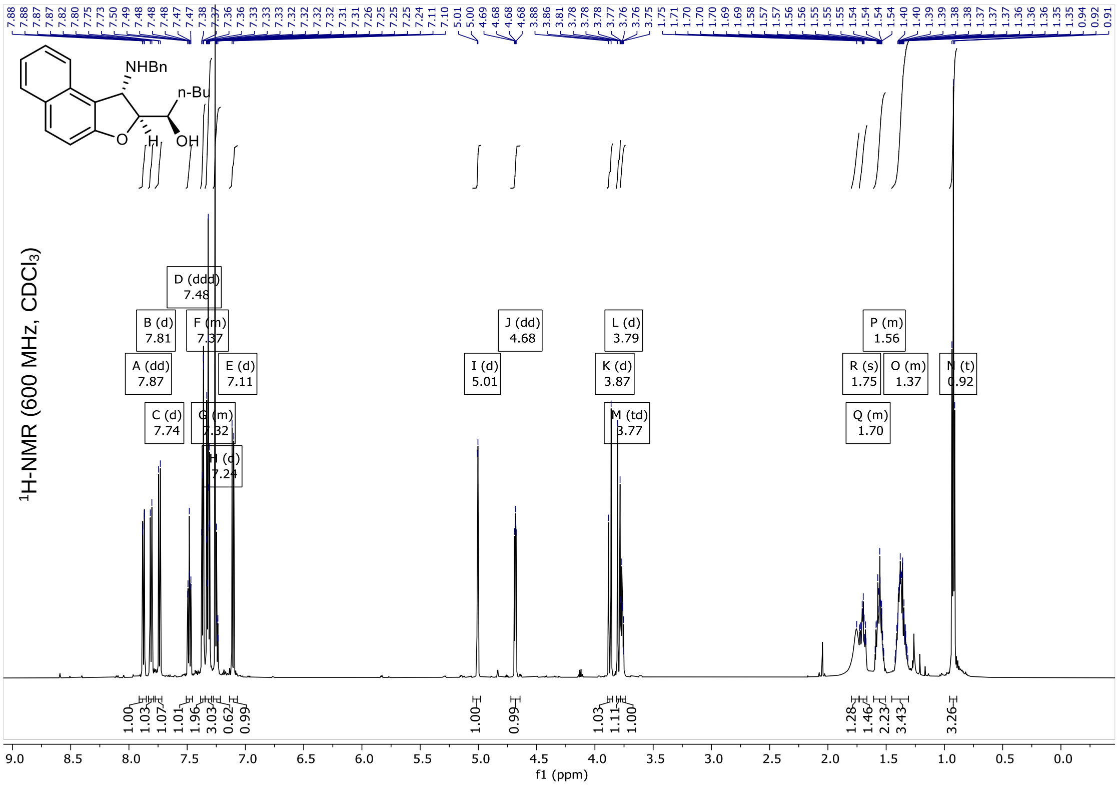

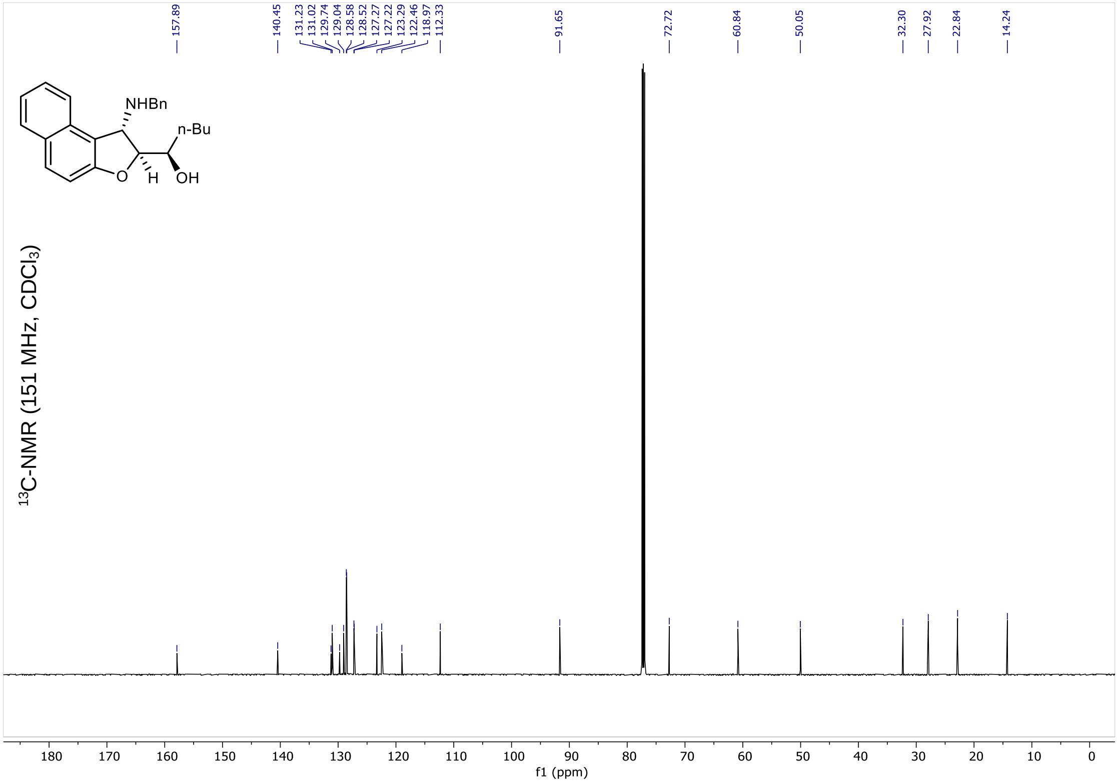

9a

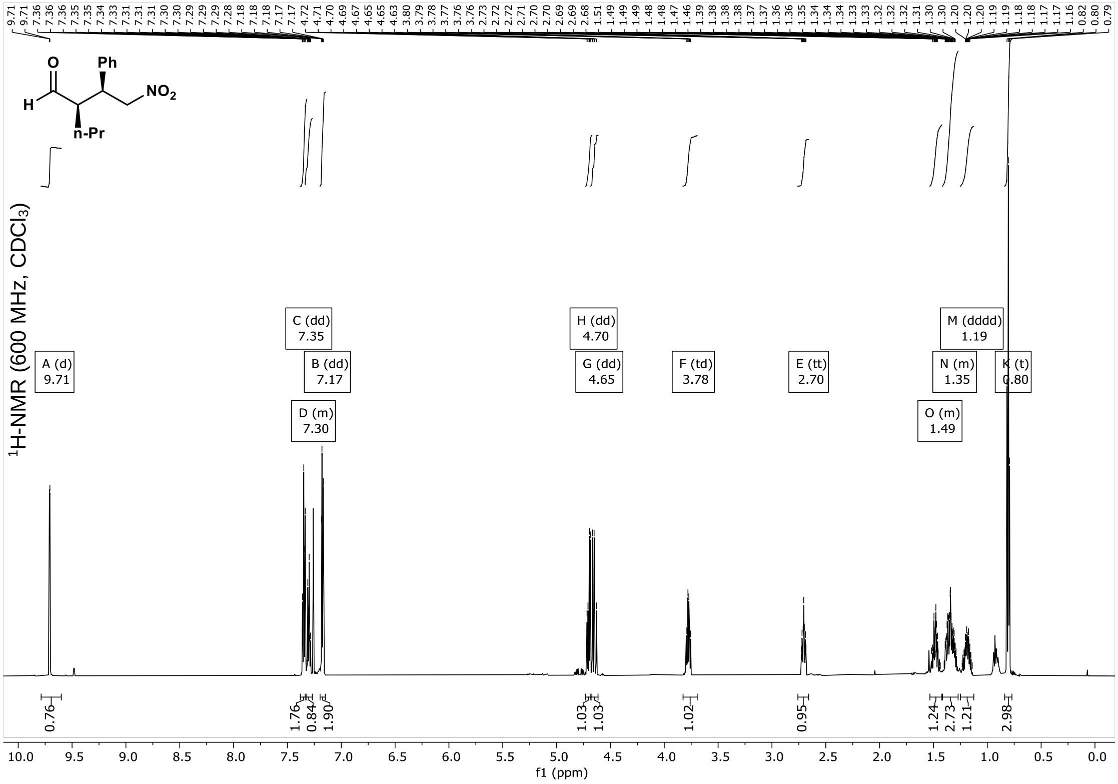

^{1}H-NMR (600 MHz, CDCl$_3$)
n-Pr
Ph
NO_2
A (d) 9.71
C (dd) 7.35
B (dd) 7.17
D (m) 7.30
H (dd) 4.70
G (dd) 4.65
F (td) 3.78
E (tt) 2.70
M (dddd) 1.19
N (m) 1.35
O (m) 1.49
K (t) 0.80
0.76
1.76
0.84
1.90
1.03
1.03
1.02
0.95
1.24
2.73
1.21
2.98
f1 (ppm)

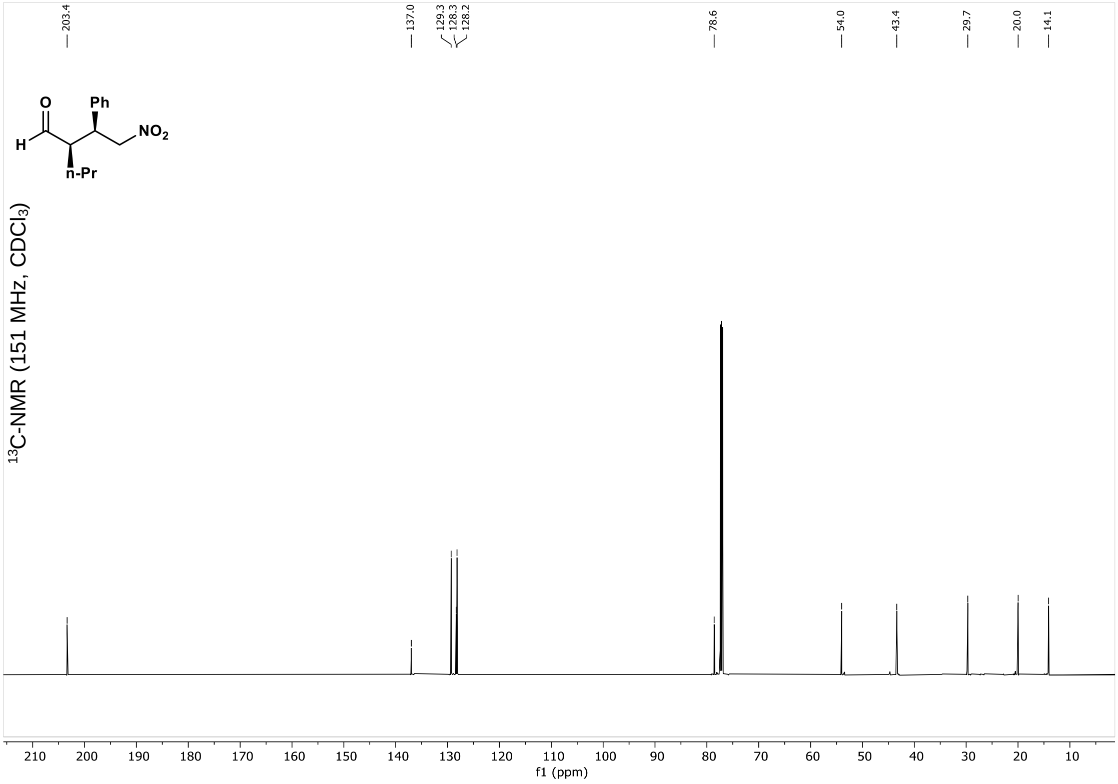

^{13}C-NMR (151 MHz, CDCl$_3$)
n-Pr
Ph
NO_2
203.4
137.0
129.3
128.3
128.2
78.6
54.0
43.4
29.7
20.0
14.1
f1 (ppm)

9b

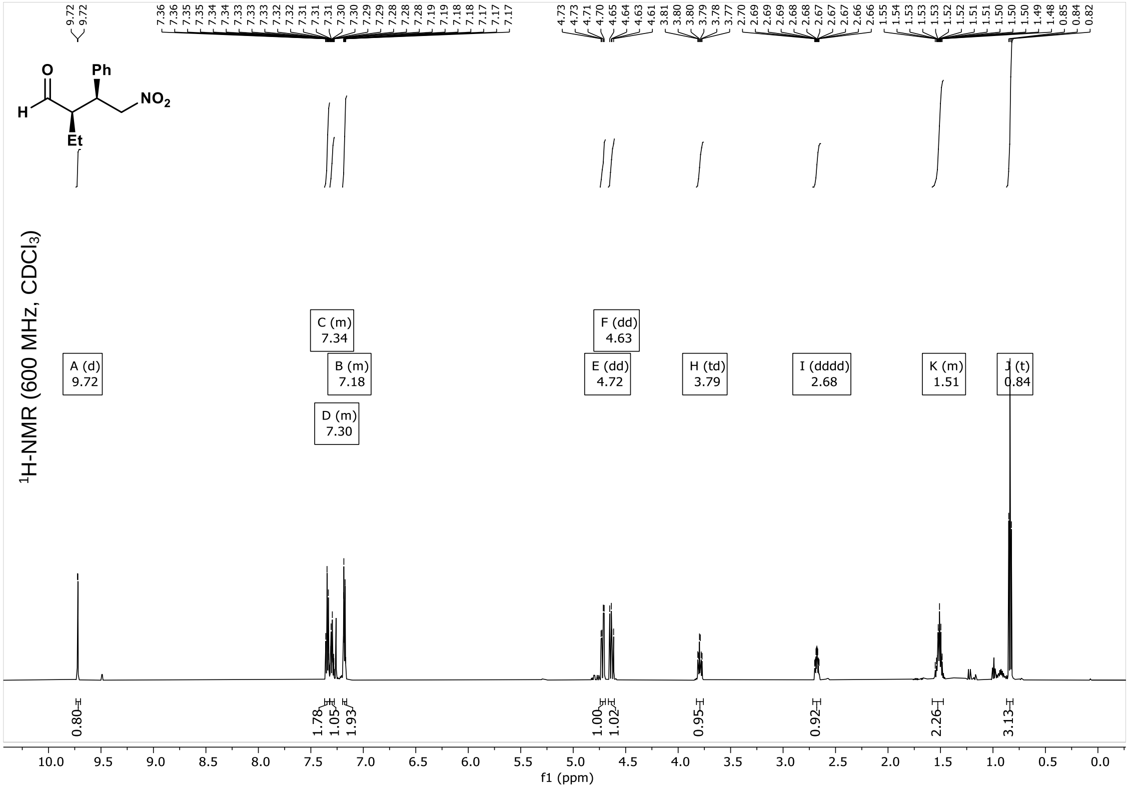

1H-NMR (600 MHz, CDCl3)
A (d) 9.72
C (m) 7.34
B (m) 7.18
D (m) 7.30
F (dd) 4.63
E (dd) 4.72
H (td) 3.79
I (dddd) 2.68
K (m) 1.51
J (t) 0.84
0.80
1.78
1.05
1.93
1.00
1.02
0.95
0.92
2.26
3.13
f1 (ppm)

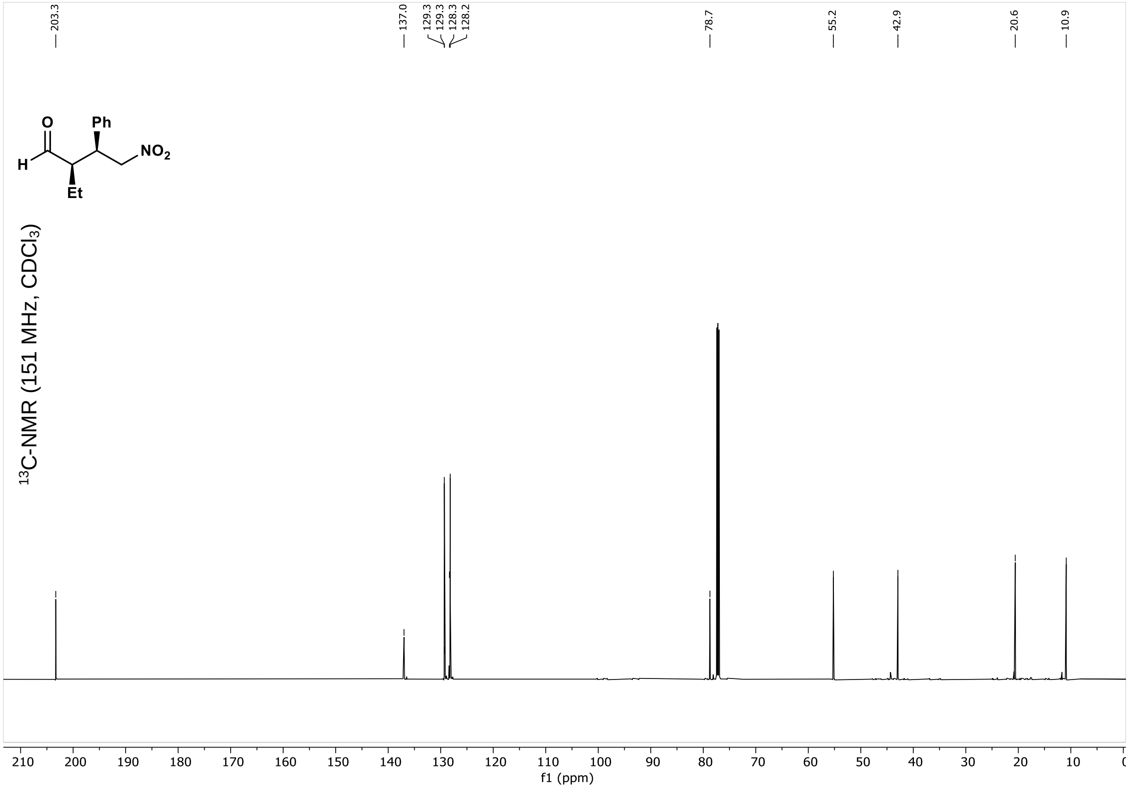

13C-NMR (151 MHz, CDCl3)
203.3
137.0
129.3
129.3
128.3
128.2
78.7
55.2
42.9
20.6
10.9
f1 (ppm)

9c

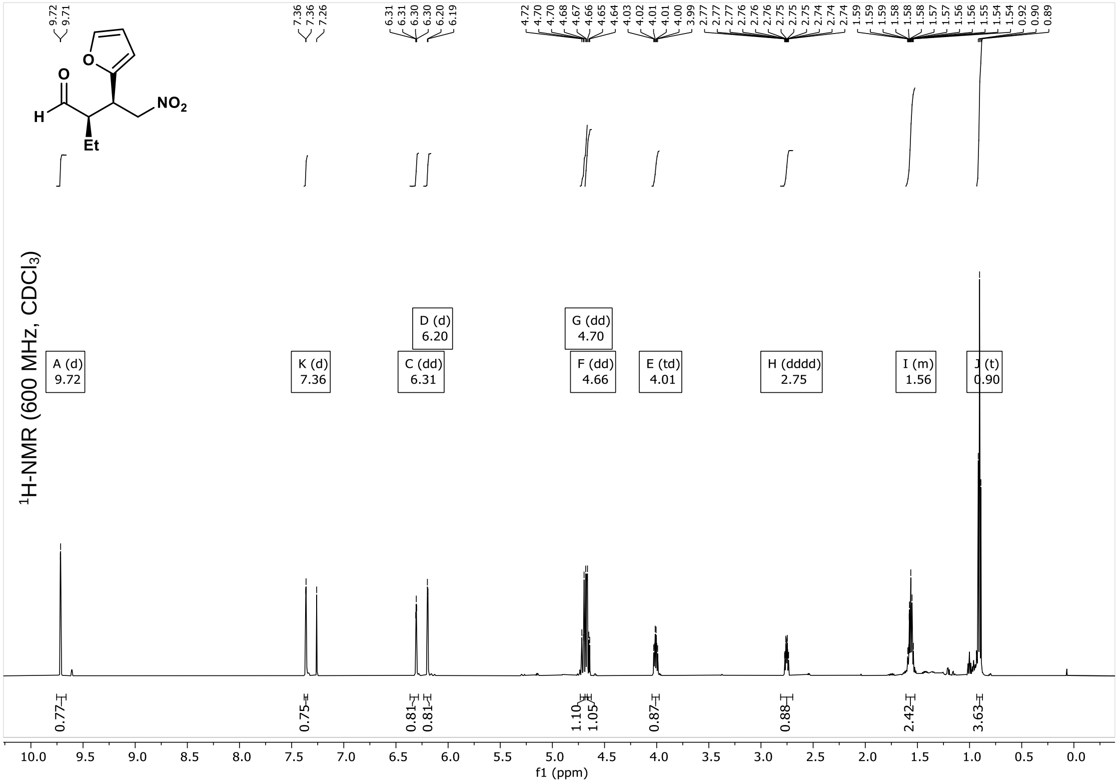
1H-NMR (600 MHz, CDCl3)
A (d) 9.72
K (d) 7.36
D (d) 6.20
C (dd) 6.31
G (dd) 4.70
F (dd) 4.66
E (td) 4.01
H (dddd) 2.75
I (m) 1.56
J (t) 0.90
0.77
0.75
0.81
0.81
1.10
1.05
0.87
0.88
2.42
3.63
f1 (ppm)

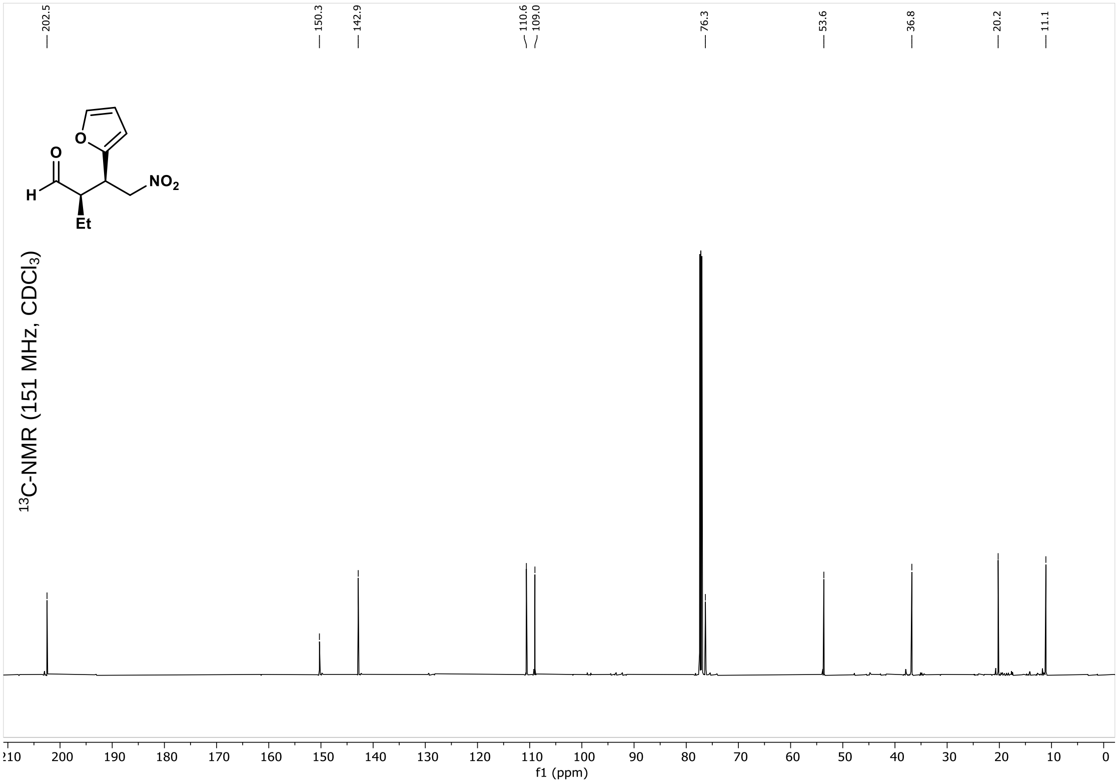
13C-NMR (151 MHz, CDCl3)
202.5
150.3
142.9
110.6
109.0
76.3
53.6
36.8
20.2
11.1
f1 (ppm)

9d

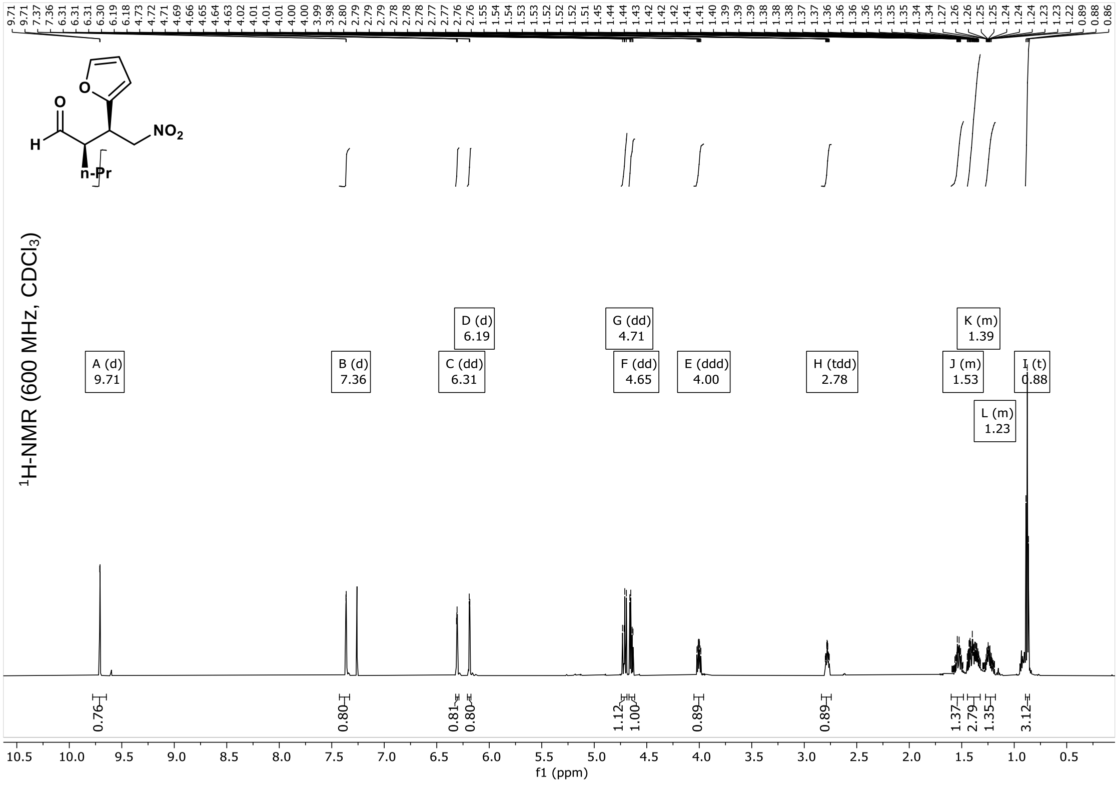
^{1}H-NMR (600 MHz, CDCl$_3$)
n-Pr
A (d) 9.71
B (d) 7.36
C (dd) 6.31
D (d) 6.19
G (dd) 4.71
F (dd) 4.65
E (ddd) 4.00
H (tdd) 2.78
J (m) 1.53
K (m) 1.39
L (m) 1.23
I (t) 0.88
0.76
0.80
0.81
0.80
1.12
1.00
0.89
0.89
1.37
2.79
1.35
3.12
f1 (ppm)

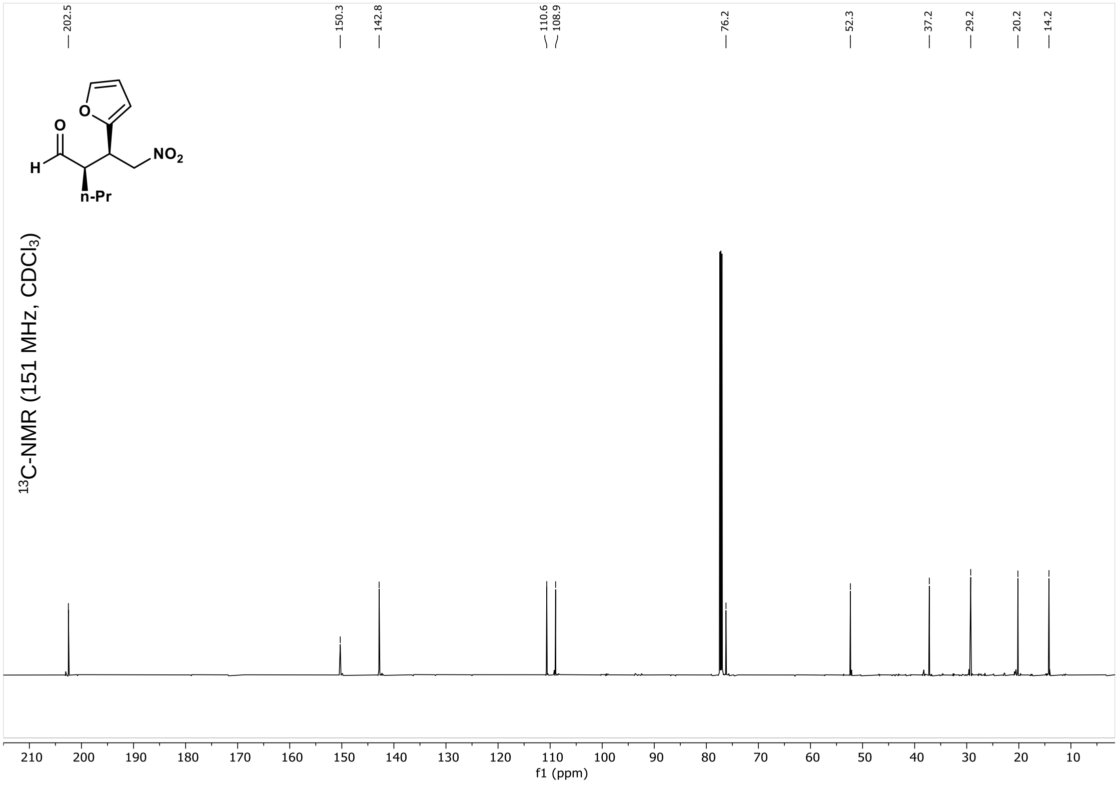
^{13}C-NMR (151 MHz, CDCl$_3$)
n-Pr
202.5
150.3
142.8
110.6
108.9
76.2
52.3
37.2
29.2
20.2
14.2
f1 (ppm)

Supplementary References

1. S. Rohrbach *et al.*, Digitization and validation of a chemical synthesis literature database in the ChemPU. *Science* **377**, 172-180 (2022).
2. https://croningroup.gitlab.io/chemputer/xdlapp/ (2023).
3. Y. Kawagoe, K. Moriyama, H. Togo, Facile preparation of amides from carboxylic acids and amines with ion-supported Ph3P. *Tetrahedron* **69**, 3971-3977 (2013).
4. A. R. Fatkulin *et al.*, Sodium hypophosphite mediated reductive amination of carbonyl compounds with N,N-dialkylformamides. *New Journal of Chemistry* **47**, 6532-6535 (2023).
5. S. R. Shengule, G. Ryder, A. C. Willis, S. G. Pyne, Highly diastereoselective N-acyliminium ion cyclization reactions of a tethered furan. *Tetrahedron* **68**, 10280-10285 (2012).
6. M. Marigo, T. C. Wabnitz, D. Fielenbach, K. A. Jorgensen, Enantioselective organocatalyzed alpha sulfenylation of aldehydes. *Angew Chem Int Ed Engl* **44**, 794-797 (2005).
7. M. D. Price, J. K. Sui, M. J. Kurth, N. E. Schore, Oxazaborolidines as functional monomers: ketone reduction using polymer-supported Corey, Bakshi, and Shibata catalysts. *J Org Chem* **67**, 8086-8089 (2002).
8. C. Y. Ho, Y. C. Chen, M. K. Wong, D. Yang, Fluorinated chiral secondary amines as catalysts for epoxidation of olefins with oxone. *J Org Chem* **70**, 898-906 (2005).
9. R. Boeckman, (S)-1,1-Diphenylprolinol Trimethylsilyl Ether. *Organic Syntheses* **92**, 309-319 (2015).
10. Z. Zheng, B. L. Perkins, B. Ni, Diarylprolinol silyl ether salts as new, efficient, water-soluble, and recyclable organocatalysts for the asymmetric Michael addition on water. *J Am Chem Soc* **132**, 50-51 (2010).
11. Y. Chi, T. J. Peelen, S. H. Gellman, A rapid 1H NMR assay for enantiomeric excess of alpha-substituted aldehydes. *Org Lett* **7**, 3469-3472 (2005).
12. G. Hutchinson, C. Alamillo-Ferrer, J. Bures, Mechanistically Guided Design of an Efficient and Enantioselective Aminocatalytic alpha-Chlorination of Aldehydes. *J Am Chem Soc* **143**, 6805-6809 (2021).
13. M. Ando, W. Tang, H. Minato, M. Ando, Determination of the Stereochemistry of C-2’ and C-3’ Positions of Taxine NA-1 (2’-Hydroxytaxine II) by the Asymmetric Synthesis of the Reductive Degradation Product of Its Side Chain Moiety. *Heterocycles* **85**, (2012).
14. S. Frankowski, J. Kowalska, A. Albrecht, Pyridylacetic acids and related systems as alkylheteroarene surrogates in asymmetric decarboxylative Michael addition. *Chem Commun (Camb)* **57**, 3387-3390 (2021).
15. Y. Wang, P. Li, X. Liang, T. Y. Zhang, J. Ye, An efficient enantioselective method for asymmetric Michael addition of nitroalkanes to alpha,beta-unsaturated aldehydes. *Chem Commun (Camb)*, 1232-1234 (2008).
16. D. Enders, M. Jeanty, J. Bats, Organocatalytic Asymmetric Triple Domino Reactions of Nitromethane with α,β-Unsaturated Aldehydes. *Synlett* **2009**, 3175-3178 (2009).

17. V. Tseliou *et al.*, Enantioselective Biocascade Catalysis with a Single Multifunctional Enzyme. *Angew Chem Int Ed Engl* **61**, e202212176 (2022).
18. L. Albrecht *et al.*, Taming the Friedel-Crafts reaction: organocatalytic approach to optically active 2,3-dihydrobenzofurans. *Angew Chem Int Ed Engl* **50**, 12496-12500 (2011).
