# Supplementary material for: Reaction blueprints and logical control flow for parallelized chiral synthesis in the Chemputer
Source: Nat Commun. 2024 Nov 26;15:10261. doi: 10.1038/s41467-024-54238-6 (PMC11599859; doi:10.1038/s41467-024-54238-6)
Supplement: Supplementary file 2 — Description of Additional Supplementary Files [file 41467_2024_54238_MOESM2_ESM.pdf]

Description of Additional Supplementary Files

Supplementary Data 1. Collection of all XDL (.xdl), graph (.json), and the resulting compiled XDL (.xdlexe) files for all automated syntheses.
